# Multiplex mutagenesis of four clustered *CrRLK1L* with CRISPR/Cas9 exposes their growth regulatory roles in response to metal ions

**DOI:** 10.1038/s41598-018-30711-3

**Published:** 2018-08-15

**Authors:** Julia Richter, James Matthew Watson, Peter Stasnik, Monika Borowska, Jana Neuhold, Matthias Berger, Peggy Stolt-Bergner, Vera Schoft, Marie-Theres Hauser

**Affiliations:** 1 Department of Applied Genetics and Cell Biology, University of Natural Resources and Life Sciences (BOKU), Muthgasse 18, 1190 Vienna, Austria; 2 Vienna Biocenter Core Facilities GmbH (VBCF), Dr. Bohrgasse 3, 1030 Vienna, Austria; 3 Gregor Mendel Institute (GMI), Austrian Academy of Sciences, Vienna BioCenter (VBC), Dr. Bohrgasse 3, 1030 Vienna, Austria

## Abstract

Resolving functions of closely linked genes is challenging or nearly impossible with classical genetic tools. Four members of the *Catharanthus roseus* receptor-like kinase 1-like (*CrRLK1L*) family are clustered on Arabidopsis chromosome five. To resolve the potentially redundant functions of this subclass of *CrRLK1L*s named *MEDOS1* to 4 (*MDS1* to 4), we generated a single CRISPR/Cas9 transformation vector using a Golden Gate based cloning system to target all four genes simultaneously. We introduce single mutations within and deletions between *MDS* genes as well as knock-outs of the whole 11 kb gene cluster. The large *MDS* cluster deletion was inherited in up to 25% of plants lacking the CRISPR/Cas9 construct in the T2 generation. In contrast to described phenotypes of already characterized *CrRLK1L* mutants, quadruple *mds* knock-outs were fully fertile, developed normal root hairs and trichomes and responded to pharmacological inhibition of cellulose biosynthesis similar to wildtype. Recently, we demonstrated the role of four *CrRLK1L* in growth adaptation to metal ion stress. Here we show the involvement of *MDS* genes in response to Ni^2+^ during hypocotyl elongation and to Cd^2+^ and Zn^2+^ during root growth. Our finding supports the model of an organ specific network of positively and negatively acting *CrRLK1L*s.

## Introduction

The ability to easily manipulate an organism’s genome is highly desirable for both basic and applied plant science^1^. Diverse methods to genetically modify plants have revolutionized basic plant research as well as agriculture, e.g. engineering crop plants to increase yield, adapt to climate change, or to improve pathogen resistance.

However, in contrast to many other organisms, targeted genome modification based on homologous recombination has proven very inefficient in higher plants^2^ making precise genome engineering difficult. With the advent of modern genome editing, especially with the development of the RNA-guided clustered regularly interspaced short palindromic repeat (CRISPR)/CRISPR-associated protein 9 (Cas9)-based genome engineering system in 2012, targeted gene editing has now also become a reality in plants^3^. Multiple reports showed efficient and heritable CRISPR/Cas9-mediated genome engineering in a variety of plant species^4–7^.

The CRISPR/Cas9 system is able to target specific genomic regions through a guide RNA (gRNA), which is designed to contain a 20-nucleotide long region complementary to the DNA sequence of interest. Cas9 then introduces a site-specific double stranded DNA break (DSB), which is repaired by endogenous DNA repair mechanisms. Error-prone non-homologous end joining (NHEJ) machinery re-joins the broken DNA ends, often introducing small insertions or deletions (indels) at the cleavage site which can cause frameshift mutations or premature termination codons. A major advantage over other methods is the option to generate multiple simultaneous modifications simply by using more than one gRNA^8^.

We adapted the GreenGate system^9^, a Golden Gate based cloning system for plants, for the modular, customizable assembly of CRISPR/Cas9 constructs. This enables the rapid generation of constructs containing any combination of a variety of promoters, plant-codon optimized Cas9 variants (Cas9, Cas9n, dCas9), protein tags, selection markers, and single or multiple gRNA coding genes.

Here, we used this system to target a genetically uncharacterized cluster of four members of the *Catharanthus roseus* receptor-like kinase 1-like (*CrRLK1L*) gene family^10–13^. The *CrRLK1L* gene family is characterized by a conserved cytoplasmic kinase domain and an extracellular region with one or two potential malectin-like sugar binding domains, which might associate with cell wall components in plants^14^. It has recently been shown that FERONIA (FER) and ANXUR1 (ANX1) and ANXUR2 (ANX2) and BUDDHA’s PAPER SEAL1 and 2 (BUPS1 and BUPS2) are receptors for secreted peptides of the RAPID ALKALINIZATION FACTOR (RALF) family^15–17^. To date, ten of the 17 members of the *CrRLK1L* gene family in *Arabidopsis thaliana* have been genetically investigated. The mutant phenotypes indicate their involvement in cell wall sensing during development and upon environmental stress^18–30^. A subgroup of four highly homologous *CrRLK1L* members is clustered in an approximately 11 kb region on chromosome five. Similar to other members of the family named after Greek (*THESEUS1*, *HERKULES1* and 2) or Roman mythology (*FERONIA*, *ANXUR1* and 2), we named the four members of this *CrRLK1L* cluster *MEDOS1* to 4 (*MDS1* to 4) after the putative half-brother of THESEUS.

The genetic analysis of closely linked genes is difficult or even impossible if double, triple, and quadruple mutant combinations are needed to resolve their putative redundant functions. Our goal was to create both single and multiple gene knock-outs of the *MEDOS* family members through a multiplexing approach using CRISPR/Cas9 editing with a single plant transformation vector. By choosing three guide RNAs (gRNAs) which together target all four genes of the *MEDOS* cluster, we were not only able to create single nonsense mutations in each gene in the cluster, but also chimeras between gene cluster members, creating every possible truncation with a single CRISPR/Cas9 editing construct. We present an efficient screening strategy that enables the identification of edited plants lacking the CRISPR/Cas9 construct in the T2 generation. Phenotypic characterization of the *mds* mutants suggests their involvement in growth responses to increased metal ion concentration. Accordingly *MDS* genes belong to a complex network of *CrRLK1L*s that both positively and negatively regulate growth.

## Results

### The MEDOS gene cluster

**Figure 1:**
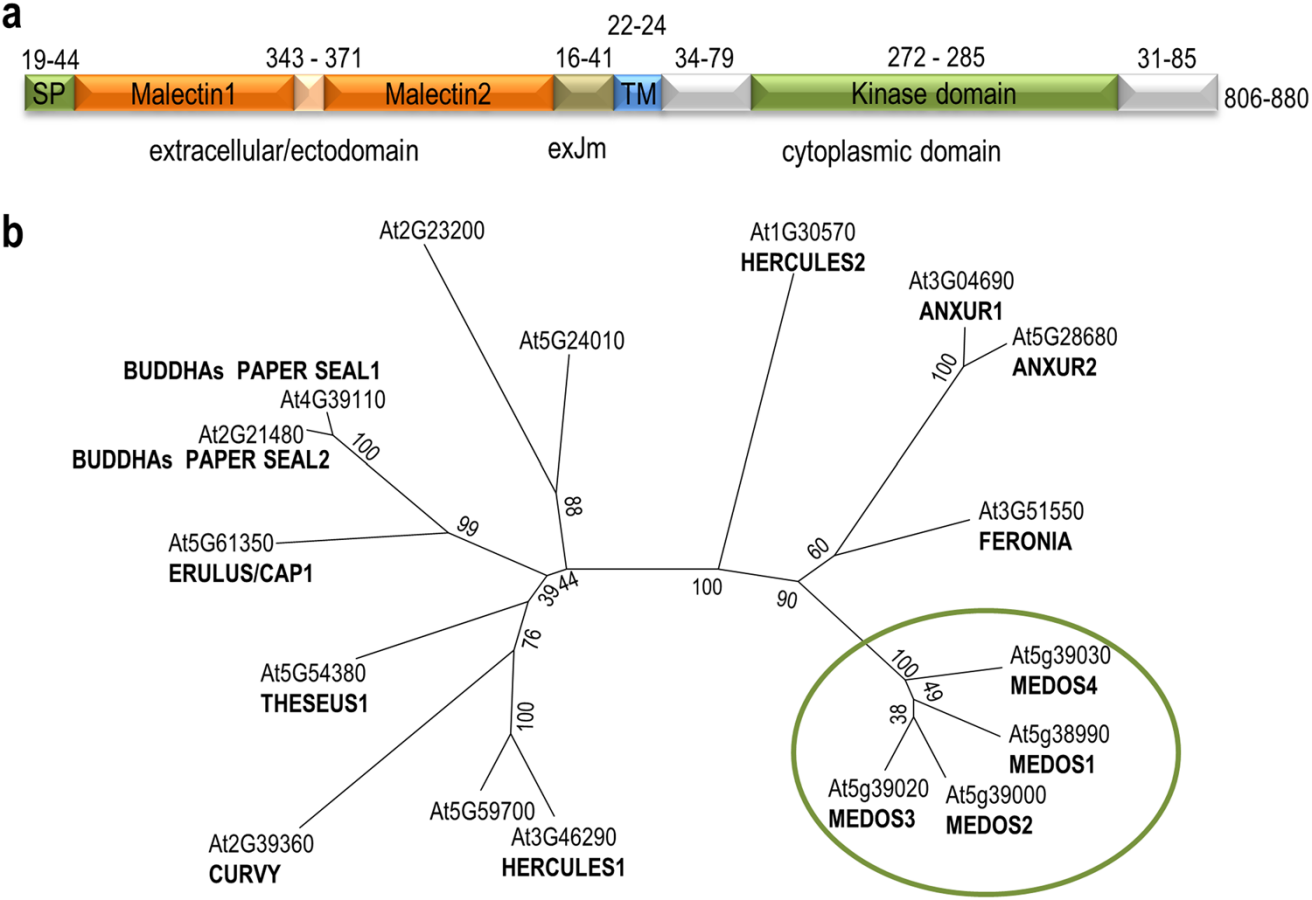
**(a)** Overview of the domain structure of the Arabidopsis *Cr*RLK1L proteins. SP, TM, and exJM correspond to signal peptide, transmembrane domain, and extracellular juxtamembrane region, respectively. Numbers above indicate the number of amino acids of the color coded domains. Numbers at the right indicate the size range of the Arabidopsis *Cr*RLK1L proteins. **(b)** Phylogenetic analysis of the extracellular domain of the Arabidopsis *Cr*RLK1L family members. Multiple alignments were calculated with ClustalW. The tree was calculated using the Maximum Likelihood method with 1000 bootstrap repeats in MEGA6.

The 17 members of the *Cr*RLK1L family share a basic structure, with one or two extracellular malectin-like domains at their N-terminus, a transmembrane domain, and an intracellular serine/threonine kinase domain at their C-terminus (Fig. 1a, Supplemental Fig. S1a,b). Sequence identity of the kinase domain varies between 28.2% and 95.1% and of the malectin-like domains between 16.7% and 87.6% (Supplemental Fig. S1c,d). Phylogenetic tree analysis of full length proteins revealed that the MEDOS proteins form two subclasses with around 44% amino acid identity (Fig. 1b; Supplemental Fig. S1c). MDS1 (At5g38990)/MDS2 (At5g39000) share an overall amino acid identity of 76.9% while MDS3 (At5g39020) and MDS4 (At5g39030) only 66.7% (Supplemental Fig. S1c). The extracellular malectin-like containing domains of MDS1 and MDS2 are 70.5% and those of MDS3/MDS4 69.1% identical (Supplemental Fig. S1d). The kinase domains are 95.1% and 82.2% identical, respectively. Apart from the four *MDS* genes, this chromosomal region contains two small open reading frames. The reading frame of At5g39024 is 96 bp and At5g39010 is 542 bp. At5g39024 encodes a peptide of 31 amino acids with sequence homology to a conserved region in the kinase domain of MDS3 and MDS4 as well as At1g66930, At1g66920, At1g66910, At1g66980, At1g66990, At1g67000, and At5g38260. RNA-Seq data indicate that At5g39024 is expressed in leaves and seedlings (Supplemental Fig. S2). At5g39010 encodes a hypothetical 169 amino acid protein with unknown function and is expressed in seedlings, leaves, and flowers (Supplemental Fig. S2).

### Design of the knock-out construct

**Figure 2:**
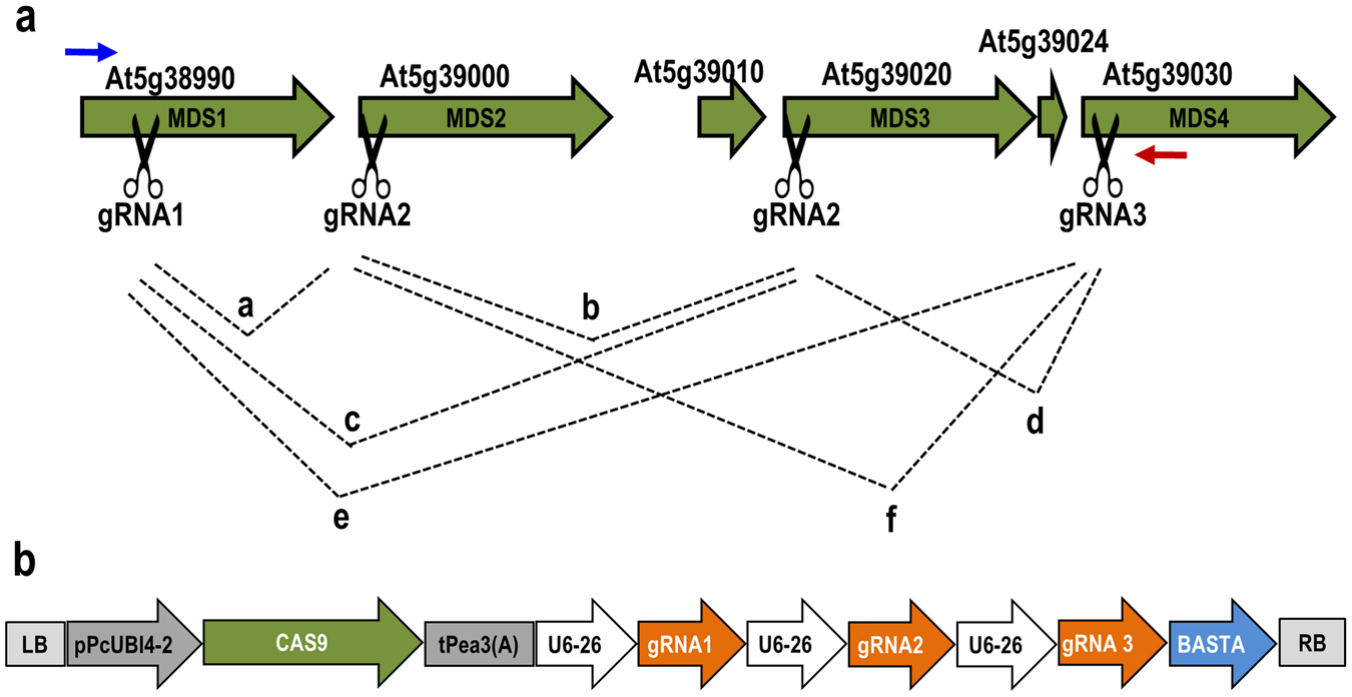
Schematic illustration of the four targeted *MDS* genes and the CRISPR/Cas9 T-DNA construct. **(a)** Open reading frames of the *MDS* gene cluster. Cas9 cutting sites are indicated with scissors. Blue and red arrows indicate primer binding sites for genotyping T1 plants. Black lines indicate all possible deletions. **(b)** The vector contains three U6–26-promoter driven gRNA modules targeting the four members of the *MDS* gene cluster. Cas9 is driven by the ubi4–2 promoter from parsley. LB, T-DNA left border; RB, T-DNA right border; pPcUBI4–2, ubiquitin4–2 promoter from *Petroselinum crispum*; CAS9, Arabidopsis codon optimized Cas9; tPea3(A), pea3A terminator from *Pisum sativum*; U6–26, Arabidopsis U6–26 (Pol-III promoter), gRNA1, gRNA2, gRNA3, gRNA/scaffold RNA modules; BASTA, glufosinate ammonium resistance gene.

To set up a flexible, modular CRISPR/Cas9 system, we cloned the promoter-, Cas9-, terminator-, gRNA scaffold-, and selection marker- sequences into GreenGate entry vectors. GreenGate entry vectors were combined into a single plant transformation vector by the Golden Gate reaction. We aimed to produce frameshift or nonsense mutations in each gene, and therefore chose gRNA sequences as close to the 5′ end as possible. Three gRNAs were chosen that target less homologous regions coding for the malectin-like domain at the 5′ end of the *MDS* genes (Supplemental Fig. S1a). Together, these three gRNAs are predicted to target all four *MDS* genes (Fig. 2a, Supplemental Fig. S1a). The final transformation vector contained the ubiquitin4–2 promoter from *Petroselinum crispum*, Arabidopsis codon optimized Cas9, the pea3A terminator from *Pisum sativum*, a glufosinate ammonium resistance gene (BASTA), and three gRNA modules each consisting of the Arabidopsis U6–26 promoter, a guide, and the gRNA scaffold (Fig. 2b).

### Analysis of the T1 generation

Transformants were selected with BASTA. Genomic DNA was isolated from rosette leaves of 27 T1 plants and analyzed by PCR. As the largest deletion (*MDS1* fused to *MDS*4) is easy to score, we designed primers that bind upstream of the gRNA1 targeting site and downstream of the gRNA3 binding site. 23 out of the 27 plants showed an approximately 11 kb deletion (Supplemental Fig. S3). Sequencing confirmed the identity of the deletion specific PCR product.

### Analyses of the T2 and T3 generation

To determine the number of T-DNA insertions, putative fertility defects, and possible lethal phenotypes, the T2 progeny of the 27 transgenic plants were evaluated for their segregation behavior on BASTA (Supplemental Table S1). Most transgenic lines contained a single T-DNA insertion based on the segregation ratio on BASTA in the T2. The following lines were chosen to select BASTA sensitive, and thus T-DNA negative, individuals: line 2, 3, 4, 9, 11, 12, 13, 18, 22, and 26. From 718 T2 plants tested, 109 were both BASTA sensitive and negative for Cas9 by PCR, confirming the elimination of the transgene (Table 1). From these 109 plants, 17 (15.6%) possessed the large deletion (Table 1). The frequency of the large deletion varied in the progeny of primary transformants from 6.7% to 25% (Table 1). Furthermore, smaller deletions were also detected (Table 1). From these smaller deletions, the most frequent was found between *MDS1* and *MDS*3 followed by deletions between *MDS1* and *MDS2*, suggesting that gRNA1 was the most effective.

**Table 1:** Summary of the screening for BASTA sensitive CRISPR/Cas-9 edited T2 plants with deletions between *MDS* genes and mutations within *MDS* genes.

Line	# plants tested	BASTA sensitive	Δ *mds* 1-4	%	Δ *mds*1-2	Δ *mds*2-3	Δ *mds* 3-4	Δ *mds*1-3	Δ *mds*2-4	*mds*1	*mds*2	*mds*3	*mds*4
2	19	7	0										
3	120	20	5	25.0	1	0	1	5	0	3	6	1	3
4	13	4	1	25.0	2	0	1	0	0				
8	20	3	0										
9	65	5	0							0	3	1	0
11	108	15	1	6.7	1	0	0	2	0	6	7	6	8
12	119	9	2	22.2	1	1	0	0	1	4	1	0	2
13	100	16	4	25.0	0	0	0	0	0	2	7	2	1
18	15	3	0										
22	119	24	4	16.7	1	0	1	1	0	2	9	3	8
26	20	3	0										
Sum	718	109	17	15.6	6	1	3	8	1	17	33	13	22

# plants tested, show the numbers of plants used to examine BASTA sensitivity with the brushing method. % refers to the percentage of BASTA sensitive plants that contain the *mds1-4* deletion. The Δ columns display the mutants with smaller deletions between the numbered *MDS* genes. The columns *mds1*, *mds2*, *mds3*, *mds4* correspond to the number of mutant alleles within individual *MDS* genes.

In addition to deletion of the entire cluster as well as deletions between only two or three *MDS* genes, mutations within the genes themselves were detected (Table 1). We identified mutant lines with mutations in one, two, three, and all four *MDS* genes. In particular, the quadruple *MDS* mutant served as a control to test for putative functions of the two additional short open reading frames in the *MDS* cluster, At5g39010 and At5g39024.

To identify homozygous lines, plants from the T3 generation were genotyped with either deletion PCRs or T7 endonuclease assays and sequencing. All deletion lines segregated in a Mendelian manner for a single locus. Since the mutations in the *MDS* genes were either heterozygous or biallelic, the T3 segregation analyses allowed us to determine whether the alleles in the different *MDS* genes are coupled or in the repulsion phase.

### Mutation spectrum of the CRISPR/Cas9 edited *MDS* gene cluster

**Figure 3:**
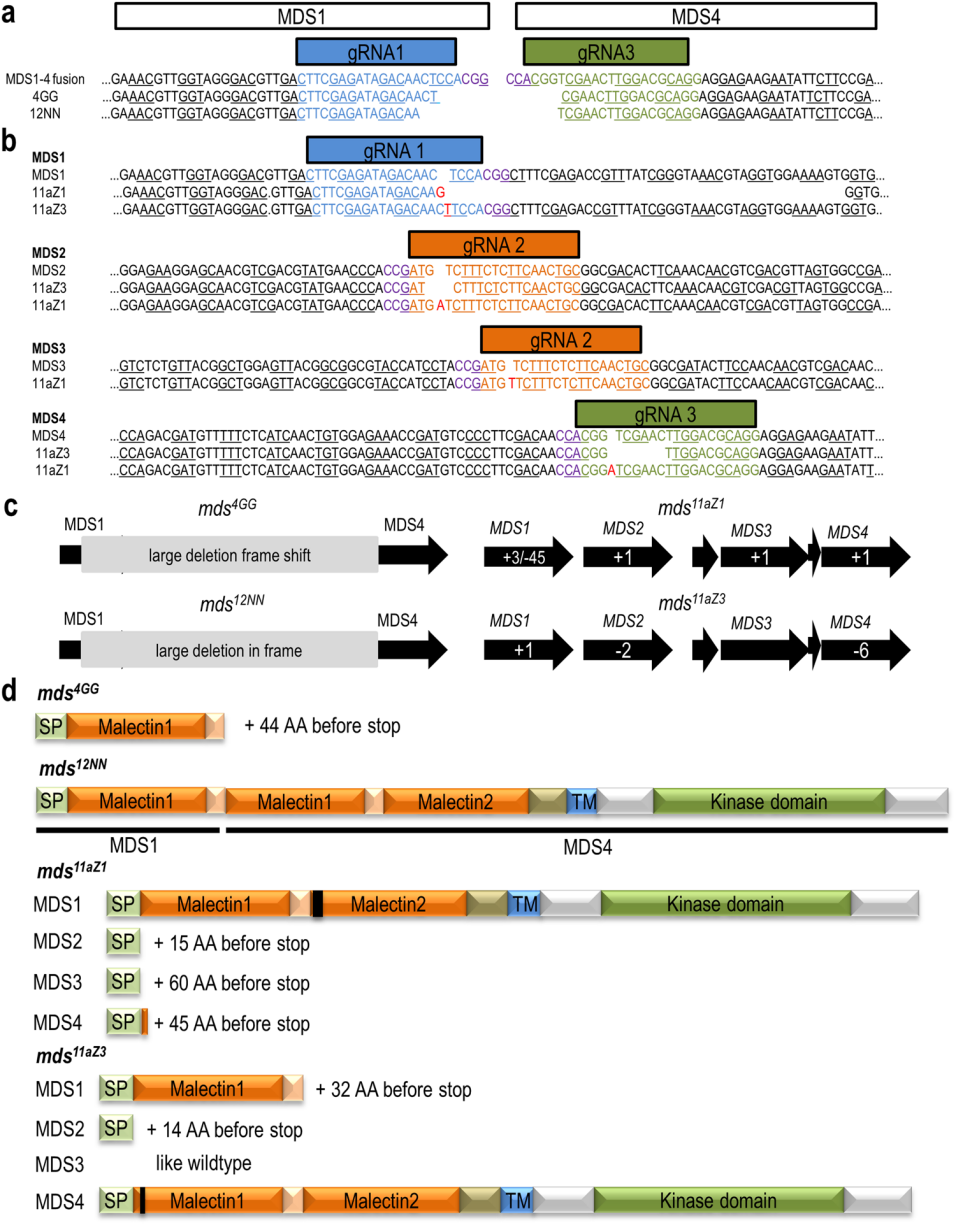
Characterization of the CRISPR/Cas9 edited mutants *mds*^*4GG*^, *mds*^1^^2^^N^^N^, *mds*^*11aZ1*^ and *mds*^*11aZ3*^. Sequences of mutants with **(a)** deletions between *MDS1* and 4 and **(b)** small mutations within individual *MDS* genes. gRNAs are marked by colored boxes and sequences, protospacer adjacent motif (PAM) motifs are indicated in purple. Underlined letters indicate the reading frames. **(c)** Schematic representation of the mutations. Numbers indicate deleted or inserted bases. **(d)** Domain structure of the putative truncated and chimeric proteins. Black bars represent deletions.

Since the gRNAs were positioned in the coding region, the mutations often caused a frameshift, resulting in a non-sense mutation with a premature stop codon (Fig. 3; Supplemental Fig. S4). The activity of gRNA1 and gRNA3 resulted in the large *MDS1* to *4* deletions with the potential to produce a fusion between *MDS1* and *MDS4* with frameshifts from the gRNA target site onwards (Fig. 2a; fragment e; Fig. 3a,c and d *mds*^*4GG*^). Only the large deletion in mutant *mds*^1^^2^^N^^N^ resulted in an in-frame fusion with the potential to express a chimeric protein with one malectin-like domain from MDS1 and two from MDS4 (Fig. 3a,c and d). The activity of gRNA1 and gRNA2 produced deletion mutants between *MDS1* and *MDS2* or *MDS3* (Fig. 2a; fragments a and c; Supplemental Fig. S4b). If only gRNA2 was active deletions between *MDS2* and *MDS3* occurred (Fig. 2a; fragment b). Deletion products of gRNA2 and gRNA3 were between *MDS2* or *MDS3* and *MDS4* (Fig. 2a; fragments d and f). Mutant lines were identified with all of these possible combinations (Table 1, Supplemental Fig. S4a and b). Additionally, some large deletions were at unexpected sites (Supplemental Fig. S3c). We found one event where gRNA1 also edited *MDS4* (*mds*^1^^2^^b^^W^) which has CTG instead of the canonical PAM motif NGG (Supplemental Fig. S4c). Another incident is the mutant *mds*^1^^2^^d^^K^ with a *MDS2–4* fusion event (Supplemental Fig. S4c). Although the *MDS2–4* fusion is positioned around gRNA3, this event is most likely due to failed repair after a gRNA2 guided cut. In mutant *mds*^3^^d^^M^, a 561 bp deletion occurred after the target site of gRNA2 and in mutant *mds*^1^^2^^a^^L^, a single adenine was inserted at the correct editing site of gRNA2 in *MDS2*, but the fusion between *MDS2* and *MDS3* happened roughly 400 bp downstream (Supplemental Fig. S1a; Supplemental Fig. S4c). In mutant *mds*^1^^1^^N^^N^, the *MDS1* target site is fused with At5g39024. Additionally, *mds*^1^^1^^N^^N^ contains a 546 bp deletion in *MDS4* which starts close to target site of gRNA3 and ends close to an off target site of gRNA1 (Supplemental Fig. S4c). This rearrangement probably has no consequences since the resulting gene fusion has a very early stop codon.

By using T7 endonuclease assays and sequencing, mutations within the *MDS* genes were identified where only one (*mds*^9^^a^^T^), two (*mds*^1^^1^^c^^M^ *mds*^9^^a^^T^, *mds*^2^^2^^d^^O^), three (*mds*^*11aZ3*^, *mds*^1^^1^^a^^V^, *mds*^1^^1^^b^^S^), or all four (*mds*^*11aZ1*^, *mds*^3^^d^^N^) *MDS* genes were mutated (Fig. 3b and c; Supplemental Fig. S4d). In the case of *mds*^*11aZ1*^, the mutation in *MDS1* does not result in a frameshift but rather a 15 amino acids deletion at the N-terminal end of the second malectin-like domain, which is highly conserved throughout all *CrRLK1L*s (Fig. 3d). In contrast, in *mds*^*11aZ3*^, the mutation in *MDS4* leads to a two amino acid deletion without a frameshift in a less conserved N-terminal region of the first malectin-like domain. In contrast, in *mds*^*11aZ3*^, the mutation in *MDS4* leads to a two amino acid deletion with a highly conserved arginine (R) of the first malectin-like domain (Supplemental Fig. S1b). A similar case is for *mds*^*3dN15*^ and *mds*^3^^d^^N^^7^. Where in *mds*^3^^d^^N^^7^ all *MDS* genes except *MDS4* are mutated, in *mds*^*3dN15*^ the highly conserve arginine (R) of *MDS4* is deleted and a amino acid substitution threonin 26 to isoleucine (T26I) and a two amino acid deletion happened in *MDS3* (Supplemental Fig S1b and S5a and b). These mutants enable the evaluation of gene specific contributions to the potentially redundant functions of the *MDS* genes and the potential redundant functions of *MDS3* and *MDS4* (Fig. 3d).

### Expression of the *MDS* genes

**Figure 4:**
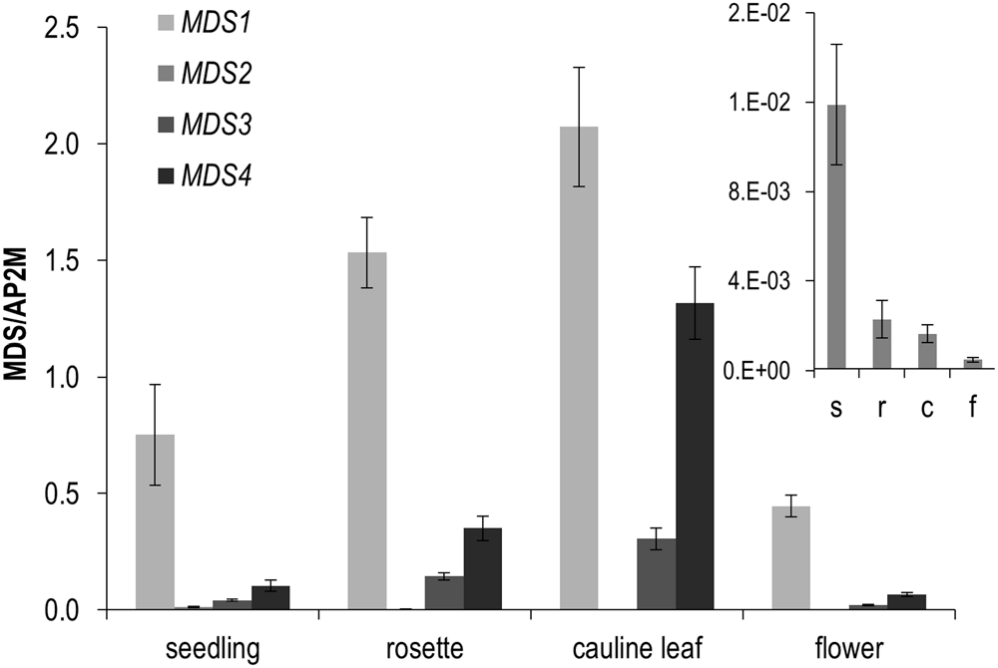
Expression of the four different *MDS* genes in different organs. Shown are the means and standard errors (SEM) of RT-qPCR data of three biological (for cauline leaves two) and three technical replicates, normalized to the reference gene *AP2M*. Abbreviations in the insert are s for seedlings, r for rosette leaves, c for cauline leaves, and f for flowers.

In reverse genetic approaches, expression analysis of uncharacterized genes indicates where and under which conditions these genes might be crucial. Quantitative gene expression evaluations are therefore a prerequisite for subsequent functional analysis. Although many microarray data are available, *MDS1* (At5g38990) and *MDS2* (At5g39000) share an oligo on the Affymetrix Arabidopsis microarray (ID 249480_s_at) and thus it was not possible to determine the individual contribution of *MDS1* and *MDS2* in microarray databases such as the Botany Array Resource (BAR)^31–33^ and Genevestigator^34,35^. Our RT-qPCR revealed that *MDS1* is the most highly expressed gene of the cluster in all organs tested followed by *MDS4* and *MDS3* (Fig. 4, Supplemental Figs S2 and S6). *MDS2* had the lowest expression (Fig. 4 insert, Supplemental Fig S6). In contrast to *MDS1*, *3*, and *4*, which are most highly expressed in rosette and cauline leaves, *MDS2* is most strongly expressed in seedlings and roots (Fig. 4, Supplemental Fig. S6). Our expression data are consistent with that of the 113 RNA-Seq deposited in the ThaleMine/Araport^36,37^ database (Supplemental Fig. S2).

As expected, the expression of *MDS1*, *MDS2*, and *MDS3* in the *mds1-4* deletion mutants was absent while the 3’end of the *MDS4* gene was detectable (Supplemental Fig. S7). As indicated above, the large deletion in the *mds*^*4GG*^ mutant leads to a frameshift and premature stop codon while *mds*^12NN^ has an in-frame fusion and potentially expresses a chimeric protein (Fig. 3d). In the mutants *mds*^*11aZ1*^ and *mds*^*11aZ3*^, all *MDS* genes were expressed. However, due to the frameshifts and premature stop codons, it is likely that the mRNAs are either degraded by nonsense-mediated decay (NMD) or trunctated proteins are synthesized (Supplemental Fig. S7).

### Phenotypic analyses of the *mds1-4* deletion mutants

Mutant phenotypes are essential for the identification of the *in vivo* role of a gene product at the cellular or organismal levels. To date, the function of eight *CrRLK1L* family members have been genetically investigated, revealing a wide range of biological roles all related to growth.

Of these ten, six are involved in fertilization. While *fer* mutant ovules are defective in pollen tube reception^21^, *BUPS1/2* and *ANX1/2* are required to maintain pollen tube integrity^17,19,22,38^. Recently, it was shown that *ERU* is involved in Ca^2+^ dependent pollen tube growth and *eru* pollen are less competitive than wildtype pollen in fertilization^39^. Based on these gametophytic phenotypes, we expected abnormal segregation ratios of the *mds1-4* deletion mutants. However, all *mds1-4* deletion mutants are recessive allele (Table 2).

**Table 2:** Segregation ratio analyses of the large deletion alleles, *mds*^*4GG*^ and *mds*^1^^2^^N^^N^. obs. is the abbreviation of observed and exp. for expected genotypes.

Allele	total	mutant [obs./exp.]	heterozygote [obs./exp.]	wildtype [obs./exp.]	Chi-Square	p-value
*mds*4GG	98	23 [24.5]	48 [49]	27 [24.5]	0.3673	0.83
*mds*12NN	83	21 [18.25]	38 [36.5]	24 [18.25]	2.2877	0.32

**Figure 5:**
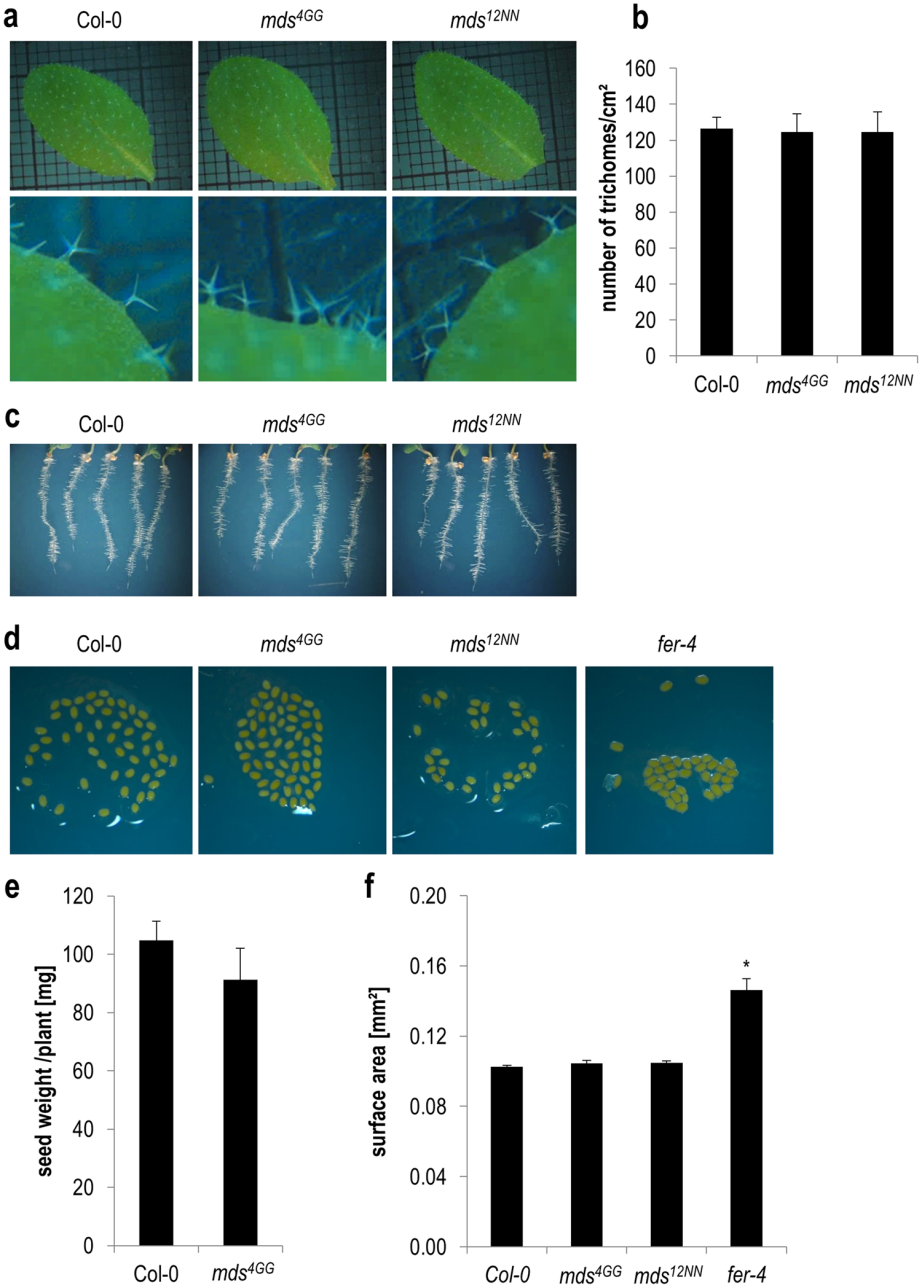
Various developmental phenotypes of wildtype and the *mds1-4* large deletion mutants. **(a**) Rosette leaves and close-ups of their adaxial surfaces, **(b)** average +/− SEM trichome density on the adaxial surface of rosette leaves of ten individuals in one experiment, **(c)** root hairs of 5 days old seedlings cultivated on MS medium supplemented with 2.5% sucrose and 1.5% agar, **(d)** imbibed seeds, **(e)** average total seed weight +/− SEM of 10 individual plants, **(f)** average seed size +/− SEM of at least 33 seeds of two to three seed stocks for wildtype, *mds*^*4GG*^ and *mds*^*12NN*^ and of one seed stock for *fer-4*. Star indicates significant difference according to Student’s *t*-test with *p < 0.05 to wildtype.

A mutant in *CURVY1* was identified due to its defective trichome and pavement cell morphogenesis^18^. Since most of the *MDS* genes are strongly expressed in leaves, we analyzed trichome patterning and morphogenesis. Neither trichome morphology nor density was altered in comparison to wildtype (Fig. 5a and b). Additional *CrRLK1L* mutant phenotypes have been published for instance for larger seeds and nearly no root hairs in *fer* mutants^25^, while *eru* mutants develop short root hairs^40^. Neither root hair phenotypes nor altered seed size or total seed weight were detectable in the *mds* mutants (Fig. 5c–f).

We then searched for phenotypes associated with gain- and loss-of-function mutants of the cell wall integrity sensor *THESEUS1 (THE1)*. *THE1* was identified in a screen for suppressors of the short hypocotyl and ectopic lignification phenotypes of cellulose biosynthesis mutants and interpreted to be a negative regulator of growth upon cellulose biosynthesis defects^41^. The loss-of-function allele *the1–6* is less sensitive to pharmacological inhibition of cellulose synthesis by isoxaben with regards to hypocotyl elongation under skotomorphogenic condition and deposition of ectopic lignin^42,43^ (Supplemental Fig. S8). Neither hypocotyl elongation nor ectopic lignification phenotypes were detectable in *mds* mutants, suggesting that the *MDS* genes are not negative regulators of growth upon cellulose biosynthesis inhibition.

### *MDS* genes function in growth responses to heavy metals and trace elements

**Figure 6:**
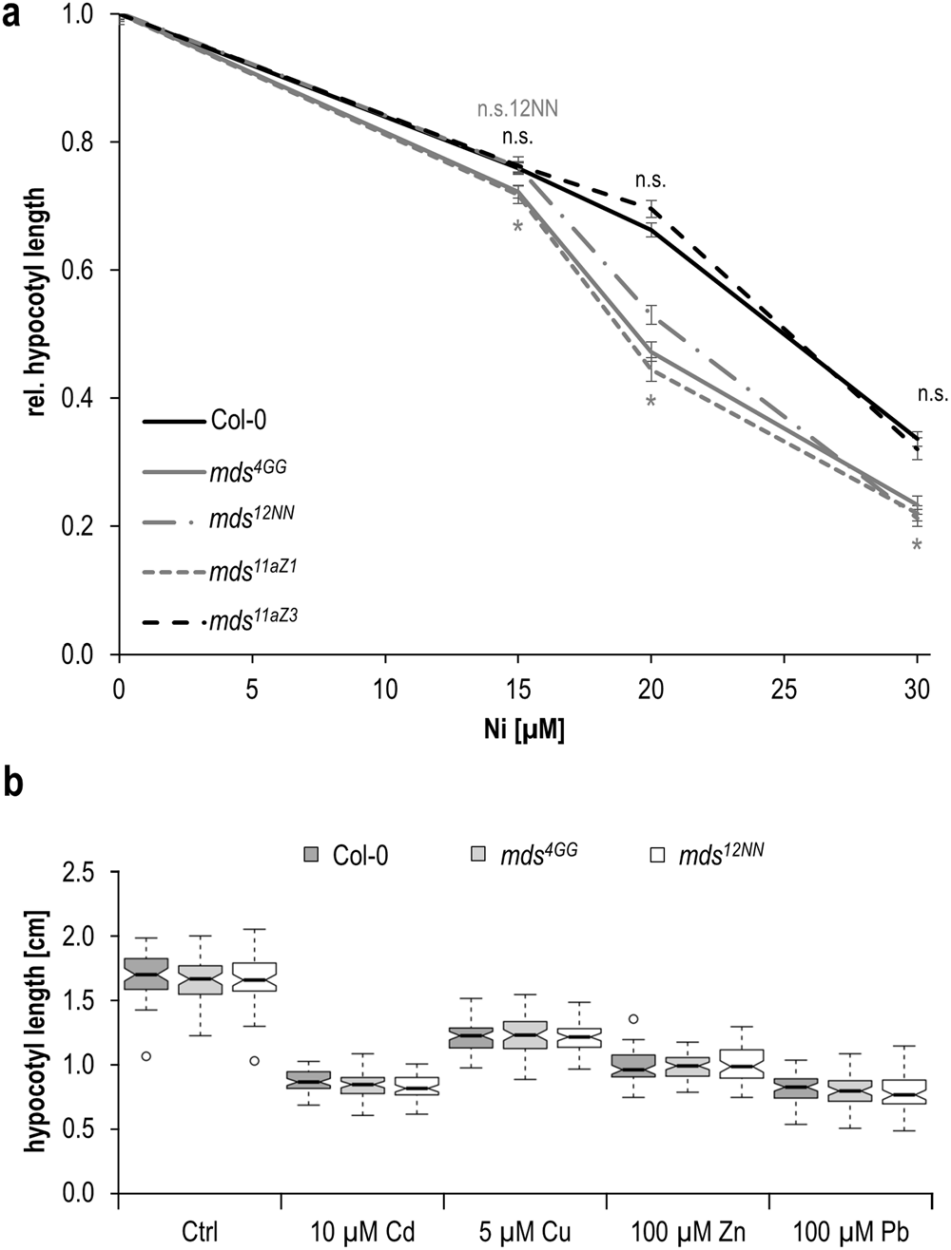
Etiolated hypocotyl length upon heavy metal and trace element exposures. **(a)** On different concentrations of Ni^2+^. Represented are means +/− SEM of up to 60 seedlings from three independent experiments. Stars indicate significant difference according to Student’s *t*-test with *p < 0.05 and (n.s.) p > 0.05 to wildtype, in gray for *mds*^*4GG*^, *mds*^1^^2^^N^^N^ and *mds*^*11aZ1*^, in black for *mds*^*11aZ3*^, **(b)** on media supplemented with Cd^2+^, Cu^2+^, Zn^2+^ and Pb^2+^. Center lines show medians of up to 60 seedlings from three independent experiments; box limits indicate the 25^th^ and 75^th^ percentiles as determined by R software; whiskers extend 1.5 times the interquartile range from the 25^th^ and 75^th^ percentiles, outliers are represented by dots.

We recently recognized four *CrRLK1L* family members to be involved in growth adaptation to heavy metals and trace elements^29^. Based on these findings, root growth and etiolated hypocotyl elongation were evaluated in the *mds1-4* deletion mutants. Similar to^29^, etiolated hypocotyl lengths were quantified on 1/10 Hoaglands medium supplemented with either Ni^2+^, Cd^2+^, Cu^2+^, Zn^2+^, or Pb^2+^ salts. On control media the different *mds* mutants developed the same hypocotyl length. However, *mds* mutants where all genes were either deleted or mutated (*mds*^*4GG*^, *mds*^1^^2^^N^^N^, *mds*^*11aZ1*^, *mds*^*3d15*^) were significantly shorter on 20 µM and 30 µM of Ni^2+^ (Fig. 6a, Supplemental Fig. S5c). In contrast, *mds*^*11AZ3*^, the allele where *MDS3* is wildtype and *MDS4* lacks only two amino acids at the beginning of the first malectin-like domain performed similar to wildtype (Fig. 6a and b). Similarly behaved the *mds*^3^^d^^7^ seedlings where *MDS4* is wildtype and *MDS3* has a nonsense mutation (Supplemental Fig. S5c). These results indicate that at least the *MDS3* and *MDS4* act redundantly since both have to be mutated to cause a growth phenotype on Ni^2+^. This growth behavior is opposite to mutants of *THE1* (*the1*), *HERKULES* 1 (*herk1*) and 2 (*herk*2), and similar to *THE1* gain-of-function alleles and the *fer-4* mutant^29^. Hypocotyl elongation of the *mds* mutants was similar to wildtype on all other metal ions (Fig. 6).

**Figure 7:**
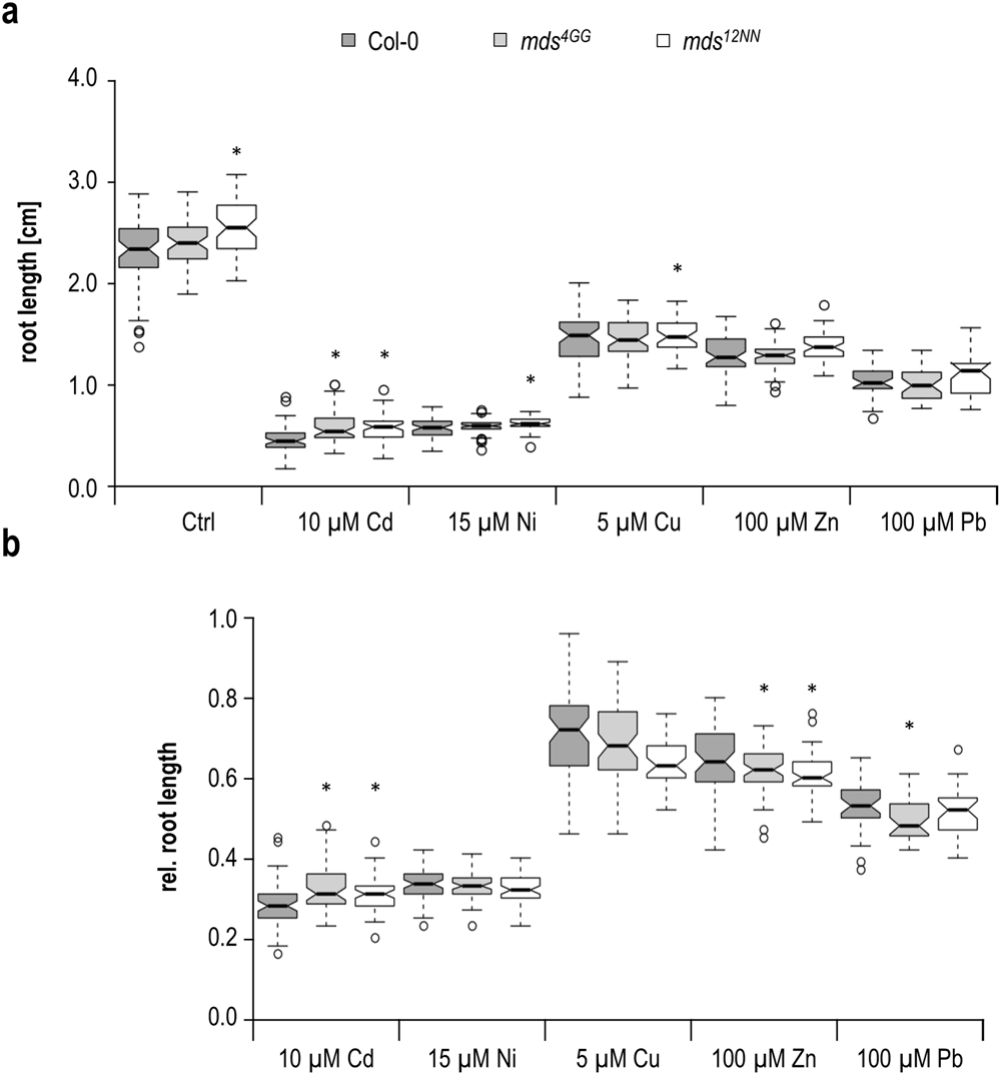
Root growth on different heavy metal and trace element exposures. **(a)** Absolute root lengths of five days old seedlings on medium containing indicated concentrations of heavy metals and trace elements. **(b)** Root lengths of **(a)** normalized to the root lengths of seedlings on control medium. Center lines show medians of up to 60 seedlings from three independent experiments; box limits indicate the 25^th^ and 75^th^ percentiles as determined by R software; whiskers extend 1.5 times the interquartile range from the 25^th^ and 75^th^ percentiles, outliers are represented by dots. Stars indicate significant difference according to Student’s *t*-test with *p < 0.05 to wildtype.

When quantifying root growth on these metal ions a different picture emerged. First, root growth was faster on control media, significantly for the *mds*^1^^2^^N^^N^ large deletion allele (Fig. 7a). We therefore normalized our data to control conditions (Fig. 7b) which revealed that both *mds* deletion alleles were less sensitive to Cd^2+^ and hypersensitive to Zn^2+^ (Fig. 7b). Cd^2+^ tolerance has not yet been observed for *CrRLK1L* mutants as *herk1*, *herk2*, and *the1* roots are shorter on Cd^2+^ containing medium compared to wildtype. The significantly reduced root growth on Zn^2+^ of the *mds* deletion alleles is similar to *herk1*, *the1–6* and *fer-4*. Taken together, our data suggest that the *MDS* genes have functional redundancies and are similar to *THE1*, *HERK1*, *HERK2*, and *FER* involved in growth adaptation upon exposure to metal ions. Therefore, the *MDS* genes contribute to the complex network of *CrRLK1L*s that positively and negatively affect growth.

## Discussion

Members of gene families in plants are often highly redundant and must be simultaneously mutated for functional analysis. Complicated crossing schemes have been the method of choice provided single mutants were available. When the family members are localized in a cluster, their mutation was an extremely difficult task, but indispensable for genetic analysis. Using the CRISPR/Cas9 system, it has become easy to knock-out several genes simultaneously^44,45^ either by using one gRNA targeting homologous regions in all genes or by using several gRNAs targeting each gene individually (or a combination of both approaches). Furthermore, it is now possible to delete whole gene clusters or large chromosomal regions^46,47^. To develop a flexible cloning system, we used GreenGate^9^ as it is a very versatile system. One simply needs to engineer gRNA modules for each genome editing target and combine it with appropriate promoters, Cas9 versions, and terminators for the goal of the specific project. Compared to Gateway-based vector systems, which produce large “scars” due to their recombination sequences^48^, the GreenGate system uses only 4 bp overhangs which can easily be designed to produce “scar-less” constructs.

As with all vector-based CRISPR/Cas9 systems, it is necessary to select plants that have lost the construct to avoid somatic effects due to continuing activity of Cas9. We decided on BASTA-sensitivity assays by brush application to single rosette leaves in the T2 generation to select CRISPR/Cas9 edited plants that lack the original T-DNA insertion. In our opinion, this selection is superior to the use of EGFP and DsRed fluorescence in the seed coat^49^ as no tedious seed selection under a fluorescent microscope is needed. Additionally, false negatives due to poor expressed fluorescing reporters are reduced. Furthermore, brushing with BASTA can be repeated if the phenotype is not indisputable. By brushing only one leaf the rest of the plant will survive and can be used to screen for the mutations generated by CRISPR/Cas9. This method is certainly superior to antibiotic selection which does not allow for negative selection. To account for the possibility that an additional truncated CRIPSR/Cas9 construct without the *BASTA* gene was inserted into the genome we also performed PCR to confirm the absence of the *CAS9* gene. While the larger deletions were easily scored with PCR and agarose gel electrophoresis, care must be taken for the accurate detection of single nucleotide polymorphisms (SNPs) and small indels. Fast prescreening for of these mutations was done with the T7 endonuclease^50^ and CEL1 assays^51,52^. While both mismatch-specific endonuclease cleavage assays provided accurate detection of single extrahelical nucleotides of indels, they were less effective at detecting single base substitutions. These specificities have already been published in other detailed surveys, demonstrating that T7 endonuclease preferentially identified insertions and deletions, whereas CEL1 (commercially available under Surveyor nuclease S) was better for recognizing substitutions^53–55^. A recent, direct comparison showed that the T7 endonuclease assay was extremely sensitive in detecting indels, with it being possible to detect one heterozygous individual out of a pool of eight samples^56^. The CEL1/Surveyor nuclease was again found to be better at detecting single-nucleotide substitutions, but the specificity and efficiency was lower due to nonspecific cleavage products^56^. In conclusion, all mismatch-specific enzyme assays underestimate CRISPR/Cas9 editing events and sequencing the target region after PCR amplification is highly recommended.

From the phenotypic analyses of the CRISPR/Cas9 generated *mds* mutants it is clear that they are not involved in signaling cascades similar to their characterized *CrRLK1L* family members during unchallenged development. Furthermore, the *mds1-4* large deletion mutants did not exhibit phenotypes associated with either loss-of-function or gain-of-function alleles of the cell wall integrity sensor THE1 under cellulose deficient conditions^41,42^. However, based on the growth assays on increased concentrations of diverse metal ions, the *MDS* genes appear to be involved in mediating adaptation to metal ion stress, a function which has been recently attributed to the *CrRLK1L* family members *HERK1* and *2*, *THE1*, and *FER*^29^. Similar to mutants of *FER*, *THE1*, *HERK1* and *2*, hypocotyl elongation was also differently affected compared to root growth in the *mds1-4* deletion mutants. Furthermore, the *MDS* genes act redundantly, since shorter etiolated hypocotyls on Ni^2+^ were only significant if all four *MDS* genes were either deleted or mutated. The *mds1-4* deletion mutants developed longer roots on Cd^2+^, a phenotype which has not been previously observed, while the response to Zn^2+^ was similar to mutants of *THE1*, *HERK1* and *FER*. Thus, the *MDS* mutants add to the complex picture of the *CrRLK1L* family as important players in a complex network of signaling cascades positively and negatively regulating growth and cell elongation. Why different metal ions trigger specific *Cr*RLK1Ls, and how they stimulate opposite growth effects, remains to be solved in the future. It is possible that the extracellular malectin-like domains directly bind metal ions since many carbohydrate binding lectins and carbohydrate-binding modules (CBM) need at least one metal ion for structural stabilization^57^ or even require metal ions such as calcium to oligomerize and bind carbohydrate ligands^58,59^. On the other hand, peptide ligands such as the FER specific RALF1^15^ and RALF23^16^, or the pollen-specific BUPS1/2-ANX1/2 receptor heteromer which bind RALF4, RALF19, and RALF34^17^ might bind metal ions. Mature RALF peptides have four highly conserved cysteines^60^ which might bind metal ions and are often involved in redox sensing and therefore in metal perception and differentiation, albeit indirectly^61,62^. The third possibility relates to the putative carbohydrate binding feature of the extracellular malectin-like domain. For ANX1 and ANX2, Boisson-Dernier *et al*.^63^ proposed possible interactions with homogalacturonans; these pectin derived cell wall compounds or their degradation products are known to bind metal ions^64^. Wolf and Höfte^65^ propose a feedback loop between RALF and pectin modifying enzymes where RALF induced alkalinization of the cell wall would activate pectin-methylesterases (PMEs). PME activity would expose demethylesterified sugar acids, which are able to complex metal ions which changes the flexibility of the cell wall.

The involvement of the *MDS* genes in adaptation to heavy metal and trace element ions might not be their sole function since. The *MDS* genes, and in particular *MDS3*, was strongly induced upon UV-B exposure in the publicly deposited microarray database (Supplemental Fig. S9). Salt stress and high osmolarity oppositely affected expression in roots and shoots and extreme temperatures generally reduced expression (Supplemental Fig. S9). Recently, it was shown that FER, THE1 and ANXUR1/2 are involved in immune responses to biotic infections^16,26,30,66–71^. Looking at the microarray expression data, the *MDS* genes, particularly *MDS3*, were strongly induced upon bacterial infection or bacterial and oomycete elicitor treatments, while fungal infections also induced *MDS4* (Supplemental Fig. S10a). Thus, our genome edited mutants provide an attractive genetic resource to dissect the importance of the *MDS* genes in pathogen infections. Further, the *mds* mutants will support functional genetic analyses of the *CrRLK1L* gene family with the goal of understanding the molecular basis of their specificities, the identification of their ligands and putative interactions and their downstream signaling networks.

## Experimental Procedures

### Plant lines and growth conditions

For transformation, we used wild-type *Arabidopsis thaliana* accession Columbia (Col-0). Plants were grown at 22 °C with a 16-h light/8-h dark cycle.

### Expression analysis by quantitative real-time PCR

Total RNA of snap-frozen seedlings grown on MS2.5 supplemented with 1% (w/v) agar (Duchefa), rosette leaves, cauline leaves, or flowers was isolated using a LiCl/CTAB method. After grinding roughly 100 mg frozen tissue, 1 mL of pre-heated RNA extraction buffer (2% [w/v] hexadecyltrimethylammonium bromide, CTAB; 2% [w/v] polyvinylpyrrolidone, PVP; 100 mM Tris/HCl pH 8.0; 25 mM EDTA; 2 M NaCl; 0.5 g/L spermidine and 2.7% [v/v] 2-mercaptoethanol) was added, mixed and incubated at 65 °C for 5 min. CTAB was removed through two extractions with 1 mL of ice-cold chloroform: isoamylalcohol (24:1) and centrifugation at 4 °C. RNA in the supernatant was precipitated with 250 µL 10 M LiCl at 4 °C for more than 1.5 h. After centrifugation and EtOH washes the pellet was dissolved in 20 µL RNase free water and stored at −80 °C. RNA was quantified with Qubit (Invitrogen) and NanoDrop (Peqlab) systems. cDNA was synthesized from 2.5 µg RNA after RNAse free DNase I digestion (Fermentas) with the M-MuLV H PLUS reverse transcriptase (Peqlab) as described^72^.

RT-qPCR expression analysis was performed using the Hot FirePol EvaGreen qPCR Mastermix (Solis Biodyne) with a Rotorgene 3000 (Qiagen). Primers for *MDS1*, *MDS2*, *MDS3*, and *MDS4* as well as the reference genes *UBIQUTIN EXTENSION PROTEIN 5* (*UBQ5*), *TUBULIN9* (*TUB*9), and *ADAPTOR PROTEIN-2 MU-ADAPTIN* (*AP2M*) are listed in Supplemental Table S2. Absolute and relative expression was calculated with a dilution series of purified PCR fragments of known molar concentration in each RT-qPCR run. Each sample was measured in triplicate from 2–3 independent cDNAs. Amplicon identity was verified by melting curve analysis.

### Sequence and *in silico* transcriptome analysis of *MEDOS* genes and proteins

For multiple sequence analysis, all Arabidopsis *CrRLK1L* gene and protein sequences were aligned with the help of the MegAlign tool of the DNASTAR (Lasergene) sequence analysis software. GeneDoc software was used for editing multiple sequence alignments^73^. Definition and sizing of the *Cr*RLK1L protein domains were done with Interpro 65.0 (http://www.ebi.ac.uk/interpro/)^74^. Multiple alignment for phylogenetic analysis of the extracellular domains was calculated by ClustalW. The tree was constructed using the Maximum Likelihood method with 1000 bootstrap repeats in MEGA6. Public transcriptomics data were consulted using the eFP browser^31,75^, Genevestigator^34,35^ and Araport in the ThaleMine data warehouse^36,37^.

### Construction of vectors

Promoter (ubiquitin4–2 promoter from *Petroselinum crispum*), Cas9 (codon-optimized for *Arabidopsis thaliana*), terminator (pea3A terminator from *Pisum sativum*) and customizable sgRNA (driven by the Arabidopsis U6–26 promoter) sequences from H. Puchta^76^ were transferred to GreenGate entry vectors (Addgene #1000000036)^9^ using Sequence- and Ligation-Independent Cloning (SLIC)^77^. Primers (no. 1–10) are listed in Supplemental Table S2.

Guide RNA (gRNA) sequences targeting the *MEDOS* family members were selected with the tool at the Broad institute (www.broadinstitute.org/rnai/public/analysis-tools/sgrna-design), examined for specificity using CRISPR-P (http://crispr.hzau.edu.cn/CRISPR/) and inserted into the guide RNA scaffold (sgRNA) GreenGate entry vectors. At their 5′ end, the gRNAs additionally contained a reverse complement sequence of the following overhangs: forward primer: attg, reverse primer: aaac (no. 11 to 16; Supplemental Table S2). After annealing, the primers were ligated into the entry vector that had been linearized with BpiI (New England Biolabs, FD1014). We used two entry vectors each with a complete gRNA module (promoter, customized gRNA, and scaffold). To insert additional gRNAs, gRNA modules were amplified by PCR and inserted into the destination vector with the Golden Gate reaction^78^. To this end, PCR primers were designed with following overhangs: E (forward primer) and P (reverse primer) for gRNA2 and P (forward primer) and F (reverse primer) for gRNA3 (primers no. 17–20 Supplemental Table S2). For the Golden Gate reaction, 150 ng of the destination vector (pGGZ003) and 250 ng of each entry vector containing the promoter, Cas9, terminator, gRNA1, and BASTA resistance were mixed with 250 ng each of the gRNA2 and gRNA3 PCR products, 0.2 µL BSA protein (New England Biolabs, B9000S), 2 µL ligase buffer, 1.2 µL T4 DNA ligase (ThermoFisher, #EL0011), 1 µL BsaI (NEB, #R0535S) and distilled water (to 20 µL). The reaction was incubated for 5 min at 37 °C followed by 5 min at 16 °C and repeated 50 times. Finally, the reaction was incubated for 30 min at 30 °C, for 5 min at 50 °C, and for 5 min at 80 °C. To eliminate not fully-ligated intermediate products, we added 0.85 µL ATP (25 mM) and 1 µL Plasmid-Safe ATP-dependent DNase (Epicentre, E3101K) and incubated for 60 min at 37 °C and for 30 min at 70 °C. Half of the reaction was transformed into chemically competent *E*. *coli* cells (NEB10beta strain) and selected on LB plates containing 100 µg/mL spectinomycin.

### Generation of stable transgenic *Arabidopsis* plants

A*grobacterium tumefaciens* GV3101 pSOUP+ cells were transformed with our plasmid using the freeze/thaw protocol^79^. *Agrobacterium*-mediated Arabidopsis transformation was performed using the floral dip method^80^. Transgenic plants were grown on soil and selected with glufosinate ammonium (BASTA) by spraying every three to four days with a solution of 20 mg/L BASTA three or four times.

### Mutant screening and sequence analysis T1 generation

A small leaf of each T1 plant was used for genomic DNA extraction. Fragments surrounding the target sites were amplified by PCR using primers At5g38990-F and At5g39030-R (no. 21 and 22; Supplemental Table S2) and the Phusion Flash High-Fidelity PCR Master Mix (ThermoFisher Scientific, F-548). The presence of the largest truncation was tested via agarose gel electrophoresis. The PCR products were sequenced with the Sanger method using primer At5g38990 seqR (no. 23; Supplemental Table S2) and compared to wildtype sequences using ApE- A plasmid Editor.

### Analysis of the T2 and the T3

To determine the number of T-DNA insertions, putative fertility defects, and possible lethal phenotypes, the T2 progeny of the 27 transgenic lines were plated on MS medium supplemented with 1% sucrose and 5 mg/L glufosinate (BASTA). After one week cultivation at 22 °C and continuous light the segregation ratio of BASTA resistant and sensitive seedlings per line was quantified (Supplemental Table S1). To isolate T2 plants lacking the T-DNA, seedlings were transferred from plates without BASTA to soil and one rosette leave was treated with a 100 mg/L BASTA solution containing 0.03% Silwet L77. Leaves of BASTA sensitive plants start to perish after two to three days. BASTA sensitive plants were additionally tested with PCR for the absence of the CAS9 nuclease gene and the presence of all combinations of *MDS* gene cluster deletions. Large deletions were identified using primers MDS1_180_F/MDS4_R. In wildtype alleles they amplify an 11.8 kb fragment and in deletion alleles a roughly 1080 bp fragment, which was subsequently sequenced. To test for homozygosity, the MDS1 specific primers MDS1_180_F/MDS1_R2 were used. Deletions between *MDS1–2*, *MDS2–3*, *MDS3–4*, *MDS1–3*, and *MDS2–4* were also determined with PCR and sequencing (for primer sequences see Supplemental Table S2). T7 endonuclease assays were used to detect mutations within each *MDS* gene. *MDS* specific PCR amplicons containing the gRNA recognition site were heated and slowly cooled to form heteroduplexes in NEBuffer 2 (New Englands Biolabs). For amplicons of homozygous mutations within genes the PCR products were mixed 1:1 with that of wildtype plants before the heating and cooling cycle. After reaching room temperature, 0.3 µl T7 endonuclease (New England Biolabs) was added and incubated for 30 min at 37 °C. The reaction was stopped by adding 1.5 μL of 0.5 M EDTA and analyzed on 2% agarose gels.

### Growth assays and phenotypic analyses

Seeds were surface-sterilized in 5% (v/v) sodium hypochlorite, 0.5% Tween-20 (v/v) solution, rinsed three times with sterile deionized water, and transferred to plates with nutrient agar containing 1/10 strength Hoagland salts, 1% (w/v) sucrose, and 1% (w/v) agar (Duchefa). For metal ion treatments, sterile salt solutions were added after autoclaving to achieve final concentrations of 10 µM CdCl_2_, 5 µM CuSO_4_, 15–30 µM NiSO_4_, 100 µM Pb(NO_3_)_2_, and 100 µM ZnSO_4_. After two days of imbibition at 4 °C in the dark, plates were vertically incubated in a growth chamber at 22 °C with constant light (80 µmol.m^−2^.s^−1^). For measurements of etiolated hypocotyls, plates were wrapped in aluminum foil after exposure to light for five hours. The plates were scanned on day five after germination (dag) for root growth and hypocotyl measurements. The lengths were evaluated with ImageJ software by freehand tracking. Isoxaben treatments, lignin staining and seed size measurements are presented in supplemental materials and methods. Statistical evaluation and notched boxplots were calculated and drawn with Excel and the R software.

### Seed size measurement with ImageJ

Seed size was measured in ImageJ. Pictures were binarised after setting the threshold in the Lab color space for b values to 129–255. The particles were then identified and the area measured automatically with the ‘Analyse Particles’ function. Two to three different seed stocks were used for *mds* deletion mutants and one for *fer-4* as control.

The datasets generated during and analyzed during the current study are available from the corresponding author on reasonable request.

## Electronic supplementary material

Supplemental Figures and Table
