# Supplementary material for: Multiplex mutagenesis of four clustered CrRLK1L with CRISPR/Cas9 exposes their growth regulatory roles in response to metal ions
Source: Sci Rep. 2018 Aug 15;8:12182. doi: 10.1038/s41598-018-30711-3 (PMC6093868; doi:10.1038/s41598-018-30711-3)
Supplement: Supplementary file 1 — Supplemental Figures and Table [file 41598_2018_30711_MOESM1_ESM.pdf]

SUPPORTING INFORMATION

Multiplex mutagenesis of four clustered *CrRLK1L* with CRISPR/Cas9 exposes their growth regulatory roles in response to metal ions

Julia Richter[1], James Matthew Watson[2,3], Peter Stasnik[1], Monika Borowska[2], Jana Neuhold[2], Matthias Berger[1], Peggy Stolt-Bergner[2], Vera Schoft[2]*, Marie-Theres Hauser[1]*

Supplementary Materials and Methods

Isoxaben treatments and lignin staining

Seeds were placed on MS media without sucrose but supplemented with 2 nM isoxaben. After two days of vernalization, plates were placed in the light for five hours to induce germination, then wrapped in aluminum and incubated vertically. Plates were scanned on day five after germination and hypocotyl length was measured in ImageJ by freehand tracking.

For lignin staining of roots, seedlings were grown for six days on half strength MS with 1% sucrose, then treated in liquid medium with or without 600 nM isoxaben overnight. Plants were fixed in 3:1 methanol/ acetic acid and stained for 15 minutes with 1% phloroglucinol in 48% ethanol/6 N HCl. Pictures were taken immediately after mounting the seedlings in water.

Figure S1. Sequence alignments of the Arabidopsis *CrRLK1L* genes **(a)** and proteins **(b)** and their phylogenetic analyses using the full length protein **(c)** or only the extracellular domain **(d)**. Indicated are the color coded gRNAs, the different protein domains (SP, signal peptide; TM, transmembrane domain), the region on *MDS3* and *MDS4* with homology to *At5g39024* and the region of the ectopic *MDS2-3* deletion in mutant line mds^{12aL}.

Figure S2. RNA-Seq data of 113 anatomical parts and growth condition of Arabidopsis Col-0 from the Araport database via the ThaleMine data warehouse. Pollen, Mature pollen; Carpel from stage 8-13 flowers; Recept., Receptacle from base of stage 15 flower of long-day-grown plants; SAM, Shoot apical meristem/young leaf from 14 days old plant grown in simulated sun condition; Infl., stage-12 floral buds from long-day grown plant; Aerial part of long-day-grown 4-leaf-stage seedlings; Leaf of 3-week-old long-daygrown plants; LGS, 7 days old plate-grown long-day seedlings; DGS, 7 days

old dark2 grown seedlings; Root from 4-leaf-stage seedlings; RAM, Root tip from 3 days old seedlings.

Figure S3. Test PCR of 27 transgenic plants of the T1 generation for a deletion between *MDS1* and *MDS4* with primers At5g38990 F-VS and At5g39030 R-VS. The gel shows that, in 23 out of the 27 plants a 300 bp to 500 bp fragment was amplified which is indicative of the roughly 11 kb deletion from *MDS1* to *MDS4*.

Figure S4. Sequence analyses of CRISPR/Cas9 edited *MDS* cluster. **(a)** Deletions between *MDS1* and *4,* **(b)** smaller gene deletions, **(c)** unexpected sites of deletions and **(d)** mutations within *MDS* genes. gRNAs are marked by colored boxes and sequences, PAM motifs are indicated in purple. Underlined letters indicate the reading frames.

Figure S5. Characterization of the CRISPR/Cas9 edited mutants mds^{3dN7} and mds^{3dN15}. **(a)** Schematic representation of the mutations. Numbers indicate deleted or inserted bases. **(b)** Domain structure of the putative truncated proteins. Black bars represent deletions. **(c)** Etiolated hypocotyl length on media supplemented with different concentrations of Ni^{2+}. Represented are means +/- SEM of up to 60 seedlings from three independent experiments. Stars indicate significant difference according to Student's t-test with (*) $p < 0.05$ to wildtype.

Figure S6. Expression of the four *MDS* genes during development. Shown are the means and standard errors (SEM) of RT-qPCR data of at least two biological replicas and three technical repeats normalized to the reference gene *UBQ5* **(a)** and *TUB9* **(b)**. **(c)** Microarray data of the development series deposited in the BAR database.

Figure S7. Expression analysis of the putative truncated or chimeric proteins. (**a**) RT-PCR results of cDNA from rosette leaves and whole seedlings grown for eight days on MS2.5. cDNA was reverse transcribed from 2 to 3 µg of RNA and diluted 1:10 and 1:5 prior to RT-PCR, respectively. PCR was performed using Phusion Taq (Thermo Fisher Scientific) for 40 cycles.

Figure S8. Phenotypes upon isoxaben treatment**. (a)** Etiolated hypocotyl length, mean of 18-20 seedlings +/- SEM. Stars indicate significant difference according to Student's t-test with (*) $p < 0.05$ to wildtype. **(b)** Hypocotyl length in relation to control medium. **(c)** Lignin staining of seedling roots exposed overnight to 600 nM isoxaben.

Figure S9. Expression of the *MDS* genes upon abiotic stressors. Shown are the microarray data of the abiotic stress series deposited in the BAR database for **(a)** shoot and **(b)** root tissues.

Figure S10. Expression of the *MDS* genes upon biotic stressors and hormone treatments. Shown are the microarray data of the **(a)** biotic stress series and the **(b)** hormone experiments deposited in the BAR database.

Table S1 Segregation analysis in the T2 generation

Line	BASTA resistant / total	not germinated	BASTA sensitive	Percentage of BASTA sensitive seedlings
1	11/16	1	4	26.7%
2	15/19	1	3	16.7%
3	14/20	1	5	26.3%
4	7/20	7	6	46.2%
5	16/19	1	2	11.1%
6	14/19	0	5	26.3%
7	15/20	4	1	6.25%
8	13/21	0	8	38.1%
9	8/20	7	5	38.5%
10	12/22	1	9	42.8%
11	19/26	2	5	20.8%
12	18/20	0	2	10.0%
13	10/22	2	10	50.0%
14	13/20	0	7	35.0%
15	19/24	1	4	17.4%
16	14/21	1	6	30.0%
17	17/21	2	2	10.5%
18	11/19	3	5	31.3%
19	14/19	1	4	22.2%
20	14/22	2	6	30.0%
21	11/17	2	4	26.7%
22	16/22	1	5	23.8%
23	12/21	1	8	40.0%
24	17/26	0	9	35.0%
25	12/21	2	7	36.8%
26	16/22	1	5	23.8%
27	18/20	2	0	poor germination
Col-0	0/20	0	20	100%

Table S2. List of Primers

No.	Name	Sequence (5' to 3')	used for
1	PcUbiA_F	ctatagaagtgaagcttggtctcaacctAAAAATTAC GGATATGAATATAGGCATATC	vector construction
2	PcUbiB_R	ctatagggcgagaattcggtctcatgttGCTGCACAT ACATAACATATCAAGATCAG	vector construction
3	PuCas9B_F	cactatagaagtgaagcttggtctcaaacaATGGATA AGAAGTACTCTATCGGACTCG	vector construction
4	PuCas9C_R	ctatagggcgagaattcggtctcaagccTCAAACCTT CCTCTTCTTCTTAGGATCAG	vector construction
5	PeaTerC_F	cactatagaagtgaagcttggtctcaggctCAGGCCT CCCAGCTTTCGTc	vector construction
6	PeaTerD_R	ctatagggcgagaattcggtctcactgaAAGCCTATA CTGTACTTAACTTGATTGCA	vector construction
7	Chimera1D_F	cactatagaagtgaagcttggtctcatcagCTTTTTTT CTTCTTCTTCGTTCATACAG	vector construction
8	Chimera1E_R	ctatagggcgagaattcggtctcagcagTAATGCCA ACTTTGTACAAGAAAGCTGG	vector construction
9	Chimera2E_F	cactatagaagtgaagcttggtctcactgcCTTTTTTT CTTCTTCTTCGTTCATACag	vector construction
10	Chimera2F_R	ctatagggcgagaattcggtctcatagtTAATGCCAA CTTTGTACAAGAAAGCTGG	vector construction
11	MTH At5g38990 F	ATTGCTTCGAGATAGACAACTCCA	gRNA1
12	MTH At5g38990 R	AAACTGGAGTTGTCTATCTCGAAG	gRNA1
13	MTH At5g39000 + 20F	ATTGGCAGTTGAAGAGAAAGACAT	gRNA2
14	MTH At5g39000 + 20R	AAACATGTCTTTCTCTTCAACTGC	gRNA2
15	MTH At5g39030 F	ATTGCCTGCGTCCAAGTTCGACCG	gRNA3
16	MTH At5g39030 R	AAACCGGTCGAACTTGGACGCAGG	gRNA3
17	Efor	gcttggtctcactgcCTTTTTTTCTTCTTC	vector construction, overhang for gRNA2
18	Prev	attcggtctcacaggTAATGCCAACTTTGT	vector construction, overhang for gRNA2
19	Pfor	gcttggtctcacctgCTTTTTTTCTTCTTC	vector construction, overhang for gRNA3
20	Frev	attcggtctcatagtTAATGCCAACTTTGT	vector construction, overhang for gRNA3

21	At5g38990-F	CAACCGTTTCACTCTCTTGC	Genotyping
22	At5g39030-R	TAGGGTAAAAGTATAAGCGGAGG	Genotyping
23	At5g38990 seqR	TTCATCGGAAAGCCAACGCC	Sequencing
24	MDS1_180_F	TCATCGAATGCAGTTGACGAC	Genotyping and sequencing
25	MDS1_R2	GTTTAGCTCAGGACTGTCTTTG	Genotyping
26	MDS2_F2	CGGTTCGTAACGGTTGAGAATA	Genotyping and sequencing
27	MDS2_5G39000_R	GAACTTTCACTGAGTATGATCTCA	Genotyping
28	MDS3_F2	CATCACACATACAACCACTAGC	Genotyping and sequencing
29	MDS3_5G39020_R	TGGATCCAGAGTTTCCAAAAGAA	Genotyping
30	MDS4_5G39030_F	TTTCTCGTTTCCGTCTCCGCA	Genotyping and sequencing
31	MDS4_F2	CAACCACTCACGCTTCATACC	Genotyping
32	MDS4_5G39030_R	TCCACCAATTAAGAACTCATAATC	Genotyping
33	5g3899_MDS1_RT_F	GGTTTTGTGCTGTAGACCGATC	Expression MDS1 - 162 bp cDNA=gDNA
34	5g3899_MDS1_RT_R	CTCACAAAACTTCTCCATCGAG	Expression MDS1 - 162 bp cDNA=gDNA
35	5g3900_MDS2_RT_F	CGAAAAATCTGACGTGTACTCC	Expression MDS2 - 162 bp cDNA=gDNA
36	5g3900_MDS2_RT_R	CCATACCACGGTCCTGAACG	Expression MDS2 - 162 bp cDNA=gDNA
37	5g3902_MDS3_RT_F	GTTGAAACCACAACTTGCAATGG	Expression MDS3 - 213 bp cDNA=gDNA
38	5g3902_MDS3_RT_R2	CTCTATCATCTCGACAACTTTG	Expression MDS3 - 213 bp cDNA=gDNA
39	5g3903_ MDS4_RT_F	GACATGATTGGAGCAAGGAGC	Expression MDS4 - 311 bp cDNA=gDNA
40	5g3903_MDS4_RT_R	TTCGGTAATAACCTCAGTGGAG	Expression MDS4 - 311 bp cDNA=gDNA
41	TUB9-F	GTACCTTGAAGCTTGCTAATCCTA	reference gene qPCR
42	TUB9-R	GTTCTGGACGTTCATCATCTGTTC	reference gene qPCR
43	3g62250_UBQ5_F	AATCGATGGATCTGGAAAGGT	reference gene qPCR
44	3g62250_UBQ5_R	CCAGAACGAAAGATGTTCAAC	reference gene qPCR
45	5g46630_AP2M_F	CAATCGATTGCTTGGTTTGGA	reference gene qPCR
46	5g46630_AP2M_R	CGAACTCGCAGACCAGATGC	reference gene qPCR
47	Cas9_F	ATGGATAAGAAGTACTCTATCGGAC	presence/absence of CAS9 gene 664 bp
48	Cas9_R	GCCTTCTTGACTTAGAGAGCCTAG	presence/absence of CAS9 gene 664 bp

Figure S1 Sequence alignments of the Arabidopsis *CrRLK1L* genes **(a)** and proteins **(b)** and their phylogenetic analyses using the full length protein **(c)** or only the extracellular domain **(d)**. Indicated are the color coded gRNAs, the different protein domains (SP, signal peptide; TM, transmembrane domain), the region on MDS3 and MDS4 with homology to *At5g39024* and the region of the ectopic MDS2-3 deletion in mutant line *mds*[12aL].

(a)

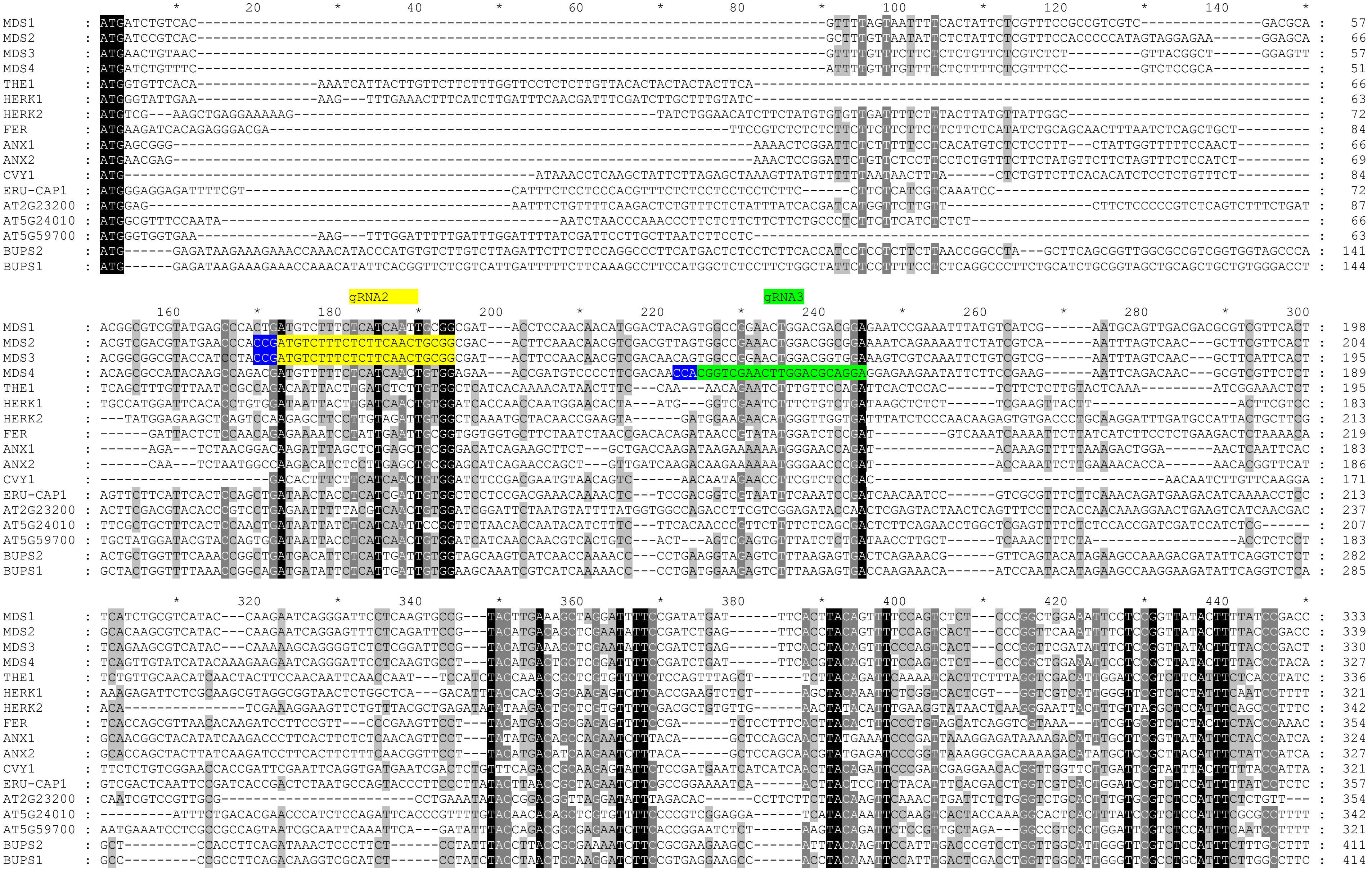

ectopic MDS2-3 deletion in 12aL

```
                 460       *       480       *       500       *       520       *       540       *       560       *       580       *       600
MDS1     : CGTTACGGATCCGATTTCGACGCCGTTAAATCCTTCTTCTCCGTCAACGTCAACCGTTTCACTCTCTTGCATAACTTCAGT------------GTA---------------------------AAAGCTTCCATACCGGAGTCAAGTTCT : 444
MDS2     : CGATACGGTTCCCAATTCAACGCCGTCAAATCCTTCTTCTCCGTCAAAGTCAACGGCTTCACTCTCTTGAACAACTTCAGCGCTGACTTAACGGTA---------------------------AAAGCATCTAAACCGCAAACGGAGTTT : 462
MDS3     : CAGTACAAATCCGGTTTTGACGCGGTTAACTCCTTCTTCTCCGTCAAAGTCAACGGTTTCACTCTCTTGCGAAACTTCAACGCTGACTCAACGGTA---------------------------CAAGCTTCAATACCGTTGTCAAATTCT : 453
MDS4     : AGTTACAAATCCGGTTTTGACGCCGTTAACTCCTTCGTCTCCGTCACCGTCAATGATTTCACTCTCTTGCAAAACTTCAGCGCCGATTTAACAGTA---------------------------AAGGCTTCAATACCAGAATCAAAATCT : 450
THE1     : AACAACTCTACT---TGGAACTTAACCTCTGCTTCAATCACTGTCGTAACAGAAGACTTCGTGCTCTTGAACAACTTCTCCTTC------------------------------------AACAACTTCAACGGT---------TCTTAC : 438
HERK1    : GACTACCAAAAC---TTCAAAATGGGTTCAGCTAAATTCGCGGTTTCTTCACAAAGTCATGTCCTTTTGAGTGATTTCACTGTT---------------------------------------------ACGAGT---------TCAAAA : 414
HERK2    : GCTATTGAAAAC---CACAATGTGAATGAGTCTTCTTTCAGCGTCTTTGCGGATGGTCTGAGACTGATGCTTGACATCAACATCGCGGGAGAAATCGCGCATAAGAATCTCATCTTGGAAAGCACTGGTCACAATGCTACTGCCTCTTCT : 489
FER      : TCGTACGACGGT---CTCAACGCTACCAACTCGTTATTCTCCGTCTCCTTTGGTCCTTACACTCTTCTCAAGAATTTCAGTGCTTCT------CAGACGGCGGAGGCGTTG---------------------------ACTTACGCTTTC : 468
ANX1     : ACATACACCGGT---CTCAACATTTCCAACTCGTATTTCACTGTGGAAGCTAACGATGTTACCTTACTGAGCAATTTCAGCGCCGCG------ATTACGTGTCAAGCTTTG---------------------------ACTCAAGCTTAT : 438
ANX2     : ACATACACAGGA---CTCAACATTCTCGACTCTTATTTCTCCGTGGCTGCTAATGATCTTACCCTTCTCAGCAATTTTAGTGCAGCT------ATCACATGTCAAGCCTTG---------------------------ACTCAAGCTTAC : 441
CVY1     : GTCTCTGCTTCT---CAAGATTTAACCACAGCTAGATTCTCAGTCTCGGCTCAGAATTCACTCTGATCAGAGAATATAAA-------------------------------------------------------CCCTCAACGACTTCT : 414
ERU-CAP1 : AATCATCCTCTC---TACAATCTAACAAACTCCGTCTTCTCCGTCACCACCGACACCACCGTCCTCTTACACGATTTC------------------------------------------------TCCGCCGGAGATACTTCTTCTATC : 456
AT2G23200: ------------------------------------------GTGTTTTCTCGGGCAGATCTTTTAACTGCTCGGTTCACTGTCTCTGCTACCTCTGGTTCTAATCATCACTTGAAAAGTTTCTCGCCTCAGAATCTCACTAATACCCCA : 462
AT5G24010: AAAGCTTCGAGA---TTTAACTTAAGGTCTGCTAAATTTCGAGTTTTGATTAATGGGTTCTCTGTGATTAACAGTTTCAGT---------------------------------------------------------ACTAGCTCCGTC : 432
AT5G59700: CAGTACCAAAAC---TTCCAAATGGTCTCAGCAAAATTCTCTGTTTCTTCAGAAACACATGTTCTTTTGAGTGATTTCACTGTG------------------------------------------------AGT---------TCCAGA : 411
BUPS2    : CCCAATGACAAG---TTTGATCTCCAACAAGCAACTTTCTCTGTTTTAACAGAGAAGTATGTCTTGCTTCATAACTTTAAACTA------------------------------------AGTAACGACAACAATGATAGCCAAGCTACT : 522
BUPS1    : CCTAATGACAAG---TTCGATCTCCAACAAGCAACCTTTTCCGTTTTAACGGAGAAATATGTGCTGCTTCATAACTTCAAAATA------------------------------------AGTAACAACAACAATGATAGTCAAGCTGCT : 525
```

```
                    *       620       *       640       *       660       *       680       *       700       *       720       *       740       *
MDS1     : CTAATCAAAGAGTTTATCGTTCCGGTTAACCAAACT------CTTGATCTCACGTTCACGCCCTCTCCG---------AATTCATTAGCTTTCGTTAACGGAATCGAGATTATCTCCATGCCTGACCGGTTTTACTCAAAGGGAGGATTT : 579
MDS2     : ATAATCAAAGAGTTTATTATTCCGGTTTACCAAACG------TTGAATCTCACTTTCACGCCGTCTTTA---------GATTCCTTAGCTTTCGTTAACGGAATCGAGATTGTCTCCATACCTAACCGGTTTTACTCAAAGGGAGGATTT : 597
MDS3     : CTAATCAAAGAGTTTATCATTCCGGTTCACCAGACT------CTGAATCTCACCTTCACTCCCTCCAAG---------AATTTGTTAGCTTTCGTTAACGGAATCGAAATCGTCTCCATGCCTGACCGGTTTTACTCAAAGGGAGGATTT : 588
MDS4     : CTAATCAAAGAATTCATCGTTCCGGTTTACCTGACT------CTGAATCTCACGTTCAGGCCGTCTAAT---------AATTCGTTAGCTTTTGTTAACGGAATCGAGATTGTCTCCATGCCTGACCGGTTTTACTCAAAGGGAGGATTT : 585
THE1     : ATCTTCAAAGAGTACACAGTCAAT---GTCACTTCAGAGTTCTTGACTTTAAGTTTCATTCCTTCAAACAAT------TCG---GTGGTCTTTGTCAACGCTATTGAAGTTGTCTCTGTTCCGGATAATCTTATCCCTGATCAAGCT--- : 573
HERK1    : GTTGTCAAAGAGTACTCTTTGAAC---GTGACTACTAATGATTTAGTGCTCACCTTTACTCCCTCTAGTGGT------TCG---TTTGCGTTTGTGAATGCTATCGAGGTTATATCGATTCCAGATACTTTGATTACTGGTAGTCCA--- : 549
HERK2    : TTGGTTAAAGAGTTTCTGTTACCT---ACTGGACCAGGAAAACTGGTTTTAAGTTTCATCCCGGAGAAAGGG------TCT---TTCGGGTTTGTCAATGCTATTGAGATAGTCTCTGTTGATGATAAGCTTTTTAAGGAATCAGTT--- : 624
FER      : ATCATCAAGGAGTTTGTTGTCAAC---GTTGAAGGTGGAACGTTGAACATGACGTTTACACCGGAATCAGCTCCGTCTAATGCGTATGCGTTTGTTAATGGGATTGAGGTTACTTCAATGCCTGAT---ATGTATAGTAGTACTGAT--- : 609
ANX1     : CTGGTTAAAGAATACTCTCTTGCACCCACTGACAAAGATGTTTTGAGCATCAAGTTTACCCCTTCAGATAAATACCGAGACGCATTTGCCTTTATAAACGGTATCGAGGTGATTCAGATGCCGGAA---TTGTTT--------------- : 570
ANX2     : CTCGTGAGAGAATATTCTCTTGCACCATCCGAAAAAGATGTTTTGAGCATCATATTTACTCCCTCGGATAAACATCCAAAAGCGTTTGCTTTCATTAATGGTATTGAGGTCATTCCGATGCCAGAA---TTGTTT--------------- : 573
CVY1     : GTTGTTAGAGAATACATTTTAAAC---GTTACCACAGATAGTCTCTTGCTTCAGTTTCTCCCTAGAACTGGT------TCT---GTTTCTTTCATCAATGCTTTGGAAGTTTTGAGACTTCCAGAGACTTTGATACCTGAAGATGCA--- : 549
ERU-CAP1 : GTCTTCAAAGAATATCTAATCTAC---GCCGCAGAG---AAACTCTCTCTTTATTTCAAACCACATAAAGGC------TCC---ACCGCTTTTATCAACGCCGTTGAAATCGTCTCTGTTCCGGACGAGCTTGTTCCGGACTCTGCT--- : 588
AT2G23200: CGAGTTGAAGAGTTTCTCCTGATG---ATGAACTCGCTGGAGTTCGAAATTCGATTTGTGCCCGAT---------CATTCCTCTTTAGCTCTCATCAATGCCATCGAAGTGTTCTCTGCTCCTGATGACCTC------------------ : 582
AT5G24010: GTTGTTAAAGAATTCATACTCAAA---ATCGATGACCCTGTTTTAGAGATTTCGTTTTTGCCCTTTAAAGCT------TCTGGATTTGGGTTTGTTAATGCGGTTGAAGTGTTTTCAGCACCTAAGGATTATATAATGGATCAAGGA--- : 570
AT5G59700: GTTATGAAGGAATACTCTCTGAAT---GTAGCTACGGATCATCTGGAGCTCACGTTTACTCCCTCGGGTGAT------TCA---TTTGCGTTTCTGAACGCGCTCGAGGTTGTTTCGGTTCCTGATACGTTGTTCAGTGGTGATCCT--- : 546
BUPS2    : GTTCAGAAAGAGTACCTCCTCAAC---ATGACCGATGCACAATTCGCCCTAAGGTTCAAGCCTATGAAAGGC------TCT---GCGGCTTTCATCAACGGTATAGAACTTGTGTCAGCTCCAGACGAACTTATCTCTGATGCAGGC--- : 657
BUPS1    : GTTCAGAAGGAGTATCTCGTCAAC---ATGACAGATGCTCAATTCGCCCTCAGGTTCAGACCTATGAAAAGC------TCT---GCAGCGTTCATCAACGCTATCGAAGTCGTGTCAGCTCCAGACGAACTGATCTCTGATTCTGGC--- : 660
```

```
                                                       gRNA1
                 760       *       780       *       800       *       820       *       840       *       860       *       880       *       900
MDS1     : GACGACGTTGTAAGAAACGTTGGTAGGGACGTTGACTTCGAGATAGACAACTCCACGGCTTTCGAGACCGTTTATCGGGTAAACGTAGGTGGAAAAGTGGTGGGCGACGTCGGAGATTCG---GGAATGTTCCGGCGTTGGCTTTCCGAT : 726
MDS2     : GACGACGTTATAACTAACGTTGGTAGTTCCGTTGACTTTCACATAGAAAATTCAACGGCTTTTGAGACAGTTTACCGGTTAAACGTAGGGGGAAAAACGGTTGGAGAT---------TCG---GGAATGTTCCGACGATGGGTCTCTGAT : 735
MDS3     : GACAACGTTTTAAGAAACGTTAGTAGCGACGTTGACTTCCAGATAGACAACTCGACGGCTTTCGAGTCCGTTCACCGGTTAAACGTCGGCGGACAAATAGTCAACGAGGTCGACGATTCA---GGAATGTTCCGGCGGTGGCTTTCTGAT : 735
MDS4     : GACGACCTTATAACTAACGTCGGTAGTTTAATTGACTTCGAGATAGACAACTCCACTGCTTCCGAGACCGTTCACAGGTTAAACGTAGGTGGACATATGGTGGACGAGGTCAACGATTCA---GGGATGTTCCGACGGTGGCTTTCTGAT : 732
THE1     : ---TTGGCGTTAAACCCTTCAACACCA---------TTTAGTGGTCTCTCTCTGCTTGCATTTGAAACAGTCTACAGATTAAATATGGGAGGACCATTGTTGACTTCTCAAAACGAT---ACA---TTGGGGAGACAATGGGATAATGAT : 705
HERK1    : ---AGGTTTGTAGGCAACCCTGCGCAG---------TTTCCGGATATGTCAATGCAAGGTCTTGAAACCATTCATAGAGTCAACATGGGTGGTCCGCTTGTTGCGTCTAACAACGAT---ACG---TTAACGAGAACTTGGGTGCCTGAC : 681
HERK2    : ---ACTAAAGTT---------GGTGGAAGTGAAGTGGAGCTTGGTTTGGGTGGACGAGGGATTGAAACTATGTATAGGCTAAACGTTGGTGGTCCCAAGCTAGGTCCAAGCAAAGATCTTAAG---CTTTATAGAACATGGGAAACAGAT : 759
FER      : ---GGGACTTTGACTATGGTTGGATCATCTGGCTCTGTTACTATTGATAACAGTACTGCTCTTGAGAATGTGTATAGGCTCAATGTTGGAGGGAATGATATCTCGCCTTCCGCGGATACGGGT---TTGTATAGGTCGTGGTATGATGAT : 753
ANX1     : ---GACACGGCGGCTCTTGTTGGTTTCACAGACCAAACAATGGATGCCAAGACTGCGAATCTTCAGTCAATGTTTAGACTTAATGTTGGTGGTCAGGACATCCCTGGAAGCCAAGACTCCGGTGGGTTGACAAGAACTTGGTATAATGAT : 717
ANX2     : ---GATACAGCTTCTCTTGTTGGATTCTCAGACCAGACTTCAGATACTAAGACCGCGAATCTTCAAACGATGTTTAGGCTCAATGTCGGTGGGCAGGATATTCCCGGAAGTCAAGACTCTGGTGGATTAACAAGAACTTGGTACAATGAC : 720
CVY1     : ---AAACTCATTGGTACACAGAAGGATCTTAAG------------CTCAGTAGTCATGCGATGGAGACTGTTTCTCGTGTAAACATGGGGAATCTTAGTGTGAGTCGTGATCAAGAT---AAG---CTGTGGAGGCAATGGGATTCTGAT : 678
ERU-CAP1 : ---TCCTCTGTTCCTCAAGCTCCTGAT---------TTCAAAGGCTTAAGTAGCTTCTCTCTTGAGATCCTTCATCGGATAAATATCGGAGGAGATTTGATTTCTCCGAAGATAGAT---CCT---CTCTCACGCACTTGGCTCTCTGAT : 720
AT2G23200: ---------------------------------GAAATCCCATCAGCTTCCGATAAGAATCTGCATACGATTTACAGATTAAACGTAGGAGGCGAGAAAATCACTCCGGATAATGATACC------TTGGGACGAACTTGGCTGCCTGAC : 693
AT5G24010: ---ACGAAGCTTGTGATTCCTAACTCTGCTCAAATCTTTAGTAACTTGTCATCTCAAGTTCTTGAGACTGTTCATAGAATCAACGTTGGTGGTTCGAAATTGACGCCGTTTAATGAT---ACG---TTGTGGAGGACTTGGGTTGTTGAT : 711
AT5G59700: ---TCCTTTGCAGGCAGTCCTGGGAAG---------TTTCAGGGCTTGTCATGGCAAGCTCTTGAGACGGTTTATAGAGTGAACATGGGCGGTCCGCGTGTTACGCCTAGTAACGAT---ACT---CTTTCGAGAATTTGGGAGCCTGAT : 678
BUPS2    : ---ACTTCTCTTTTCCCTGTCAACGGT---------TTTTCGGGTTTGTCTGATTACGCATACCAGTCCGTTTACAGGGTTAATGTTGGTGGCCCCTTGATCACGCCACAGAATGAC---ACA---CTAGGAAGAACTTGGACACCTGAC : 789
BUPS1    : ---ACTGCCCTTTTCCCTGTCATTGGG---------TTCTCGGGTCTGTCTGACTACGCTTACCAGTCTGTTTATAGAGTCAATGTCGGCGGTCCCTTGATCATGCCTCAGAATGAT---ACA---TTAGGAAGAACCTGGATACCCGAT : 792
```

```
                      *        920         *        940         *        960         *        980         *       1000         *       1020         *       1040         *
MDS1       : GAAGGTTTCTTACTCGGTATTAATTCGGGAGCCATTCCGAATATAACAGGTGTAAAG------ATCAACTACACGGATAAA---------ACTCCCGCGTACGTTGGGCCGGAAGATGTATACACGACGTGTCGCCTGATGGGGAACAAA :  861
MDS2       : GATGAGATCATACTCAGTGAAAGTTCAGGAATCTCTCCGATCGTACCAGATATAAAG------ATTAACTACACGGAGAAA---------ACTCCGTCGTACGTTGGGCCGGATGACGTGTACGCCACGTCTCGCTCAATGGGAAACGCG :  870
MDS3       : GAT---TCTTTTGGA------AACTCTGGATCCATTGTGAATGTTCCGGGTGTAAAG------ATCAACTACACGGAGAAG---------ACTCCGGCGTATGTTGGGCCGTATGATGTGTATGCTACTAGTCGCTTAATGGGGAACTCC :  861
MDS4       : GATTATGAGTTCTTA------ATTGGTGGAGTCAGTCCATATATGCAGATGTAAAT------ATCAGCTACACTGAGAAA---------ACTCCGCGTATGTTGGGCCAGCTTACGTGTACTCCACGTGTCGCATGATGGGGAACGCC :  861
THE1       : GCAGAG---TATCTTCATGTGAACAGCTCTGTTCTTGTTGTAACGGCGAATCCTTCT---TCGATTAAGTACTCTCCTTCT---------GTGACTCAAGAAACAGCTCCTAACATGGTTTATGCAACTGCTGATACAATGGGT---GAT :  837
HERK1      : TCGGAG---TTTCTG---CTTGAGAAGAATTAGCTAAGAGTATGTCTAAGTTTTCA---ACTGTTAACTTTGTTCCAGGT------TATGCAACAGAGGACTCTGCTCCAAGAACTGTCTATGGTAGTTGTACTGAGATGAAT---TCC :  813
HERK2      : TTAAGC---TACATGGTGATTGAGAACGCTGGTGTAGAAGTCAAG---AACAGCTCA---AATATCACATATGCTTTGGCT------GATGATTCTCCT---GTGGCTCCTCTTCTTGTTTATGAAACTGCTAGGATGATGTCA---AAC :  888
FER        : CAGCCT---TATATATTTGGTGCAGGACTTGGTATTCCAGAGACTGCTGATCCCAACATGACGATTAAGTATCCTACGGGG---------ACTCCTACTTATGTTGCTCCTGTGGATGTTTATTCAACCGCGAGGTCTATGGGTCCAACA :  891
ANX1       : GCACCT---TACATCTTTAGTGCGGGTCTTGGTGTTACACTTCAAGCAAGCAACAATTTTAGGATCAACTACCAGAAC------------ATGCCGGTGTCTATAGCTCCAGCCGATATATACAAAACCGCAAGATCTCAAGGCCCAAAC :  852
ANX2       : GCGCCT---TATATATTCAGCGCAGGTCTTGGTGTTACTTTACAAGCAAGCAACAATTTTAGAATTGATTATCAGAAA------------ATGCCGGTTTCTACCGCTCCAGCGGATGTTTACAAAACAGCTCGATCACAAGGACCAAAC :  855
CVY1       : TCTGCT---TAT---------AAAGCTCACTTCGGTACACCGGTTATGAACCTCAAA---GCGGTTAACTTCAGTGCTGGT------GGAATAACTGATGACATTGCACCAGTTTACGTCTATGGAACCGCCACGAGGTTAAACTCTGAT :  807
ERU-CAP1   : AAACCT---TACAATACGTTCCCTGAAGGTTCGAGAAATGTCACTGTTGATCCTAGT---ACGATTACTTATCCAGACGGT------GGAGCTACGGCGTTGATCGCTCCTAATCCGGTTTACGCCACCGCGGAGGAAATGGCC---GAT :  855
AT2G23200  : GATGATGACTTTCTCTACCGAAAAGATTCAGCGAGGAACATTAATTCAACACAA------ACGCCTAACTACGTGGAGGGTTAAGTTCAGCCACGGACTCCACTGCTCCTGATTTCGTCTACAAGACAGCTAAAGCAATGAACCGTAGC :  837
AT5G24010  : GACAAC---TATCTTCTCTTGAGAGCTGCTGCTAGACGTGCTTGGACTACTCATTCT---CCG---AATTATCAAAACGGT------GGTGCCACGAGAGAGATTGCTCCGGATAATGTTTATATGACTGCTCAGGAGATGGATCGAGAT :  846
AT5G59700  : TCTGAG---TTTCTA---GTTGAGAAGAATCTAGTTAAGAGTGTGTCTAAGATTGCA---TCTGTTGATTATGTTCCTGGT------TTTGCTACAGAGGAGACTGCTCCTAGAACTGTCTATGGTACTTGCACCGAGATGAAT---TCT :  810
BUPS2      : AAAGAA---TACTTAAAAGATGAGAATCTAGCTAAGGATGTTAAAACAAACCCTACA---GCGATCATCTATCCTCCTGGA---------GTTACACCGCTGATAGGCCCACAAACGGTTTACGCAACAGGAGCAGAGATGGCT---GAT :  921
BUPS1      : AAGGAA---TTCTTGAAAGATGAGAACTTGGCAAAGGACGTTAAAACAACCCCTTCG---GCAATCAAGTACCCACCGGAA---------GTTACACCTTTGATTGCTCCACAGACGGTTTATGCAACAGCTGTAGAAATGGCT---AAT :  924

                   1060         *       1080         *       1100         *       1120         *       1140         *       1160         *       1180         *       1200
MDS1       : GACAGTCCTGAGCTAAACCTGAATTTCAACCTGACGTGGCTCTTT---GAAGTCGATGCCGGGTTTGCCTATATAGTGAGGCTTCATTTCTGTGAGACGCAACCGGAAGTCAACAAAACGGGTGACCGCGTC---TTCTCCATCTTCTTC : 1005
MDS2       : GATCACCCTGAGCAAAACCTAAATTTTAACCTGACTTGGTTATTC---ACCGTTGATGCTGGGTTTAGCTACCTTGTGAGGCTTCATTTCTGTGAGACTTTGTCAGAAGTGAACAAAGAGGGTCAACGCGTC---TTCTCTATCTTTATT : 1014
MDS3       : AGCAAC------------TTGATGTTCAATCTAACGGGTATGTTCCTCACAGTTGATGCTGGGTATAACTACCTTGTGAGGCTACATTTCTGTGAGACTCTTCCACAAGTTACCAAAGCAGGCCAACGTGTC---TTCTCCATATTCGTC :  996
MDS4       : CAAGACACTTATCTAAACCTGAATTTCAACCTGACCTGGCTCTTT---ACAGTCGATGCCGGGTTTAGTTACCTTGTGAGGCTTCATTTCTTTGAGAAGTAC------CTGAACAAAGCCAATCAACGCGTC---TTCTCCATCTTCCTT :  999
THE1       : GCTAATGTT---GCGAGTCCAAGTTTTAATGTTAGTTGGTTCTTCCTGTTGATCCAGAC---TTCAGGTACTTGTTCGTGTTCATTTCTGTGATATTGTGAGTCAAGCTTTGAAC---------ACGCTTGTTTTCAATCTTTATGTG :  972
HERK1      : GCTGATAAC---CCGAATAGCATTTTCAATGTTGACTTGGGAGTTCGATGTTGACCCGGGT---TTCAGTACTATTTCCGCTTCATTTCTGCGATATCGTTAGCTTGTCGTTAAAC---------CAGCTATATTTCAATCTTTATGTT :  948
HERK2      : ACTGAAGTC---TTGGAGAAACGGTTCAACATTTCTTGGAAGTTTGAAGTTGATCCTAAT---TTCGACTACTTGGTTAGGCTTCATTTCTGTGAGCTTCTTGTTGATAAGCAAAC---------CAGAGGATTTTCAGGATATACATA : 1023
FER        : GCTCAG------ATCAATCTCAACTACAATCTTACTTGGATTTTCAGCATTGACTCTGGT---TTCACTTACCTTGTTAGACTTCATTTCTGTGAGGTTTCTTCGAATATCACTAAGATCAACCAACGGGTG---TTTACAATCTACCTC : 1029
ANX1       : GGAGAT------ATCAACCTCAAATCCAATCTTACATGGATGTTTCCAAATCGATAAGAAC---TTCACCTACATTTGAGACTCCATTTCTGCGAATTC------CAGCTTTCCAAGATCAACCAGAAGGTT---TTCAACATTTACATC :  984
ANX2       : GGAGAT------ATCAACCATGAAGTCAAATCTCACATGGATGTTTCAAGTTGACACTAAT---TTCACCTATATCATGAGGCTCCACTTCTGTGAATTC------CAGCTTGCTAAAATCAACCAGAAAGTA---TTCAACATTTCATC :  987
CVY1       : ---CTTGAT---CCTAATACTAACGCCAATCTTACTTGGACATTCAAAGTCCAGCCTGGT---TTCGATTACTTTGTTCGGTTTCACTTTTGTAACATAATAGTGGATCCTTTTGGATTCGAGCGTCAGATACGTTTGACATTTTTGTG :  948
ERU-CAP1   : GCACAAACC---TCGCAACCTAATTTCAATCTCTCATGGAGAATGAGTGTTGATTTTGGT---CATGATTACTTCATTAGGTTACATTTTTGTGACATTGTAAGCAAATCGCTTAAC---------GACCTTATCTTCAATGTTTTCATC :  990
AT2G23200  : TCGAATGAGCAGGTGGGGATGTTGATGAATGTTTACATGGTCGTTCAAGGTCAAAAGTAACCAT---AGACACTTCATCAGGATTCATTTTTCTGATATTTGAGCAACTTATCAAACTCCGACTCCGAT------TTCTATCTCTTTGTA :  978
AT5G24010  : AATCAGGAG---TTGCAGGCAAGGTTTAACATTAGTTGGGGTTTCAAGGTTGATGAGAAACGAGTTCTTCATTTGGTTCGTTTGCATTTCTGCGATATCGTGAGTTCGTCGCTTAAT---------CAGCTCTATTTCAATGTCTTTATC :  984
AT5G59700  : GCGGATAAC---CCGTCTAGCAACTTCAATGTTGACTTGGGATTTCGATGTTGACCCGGGT---TTCAGTACTTTCTACGGTTTCATTTCTGCGATATCGTGAGTAAAGCTCTAAAC---------CAGCTTTACTTCAATCTTTATGTC :  945
BUPS2      : TCTCAAACC---ATAGATCCTAACTTCAATGTTACATGGAATTTCCCATCAAACCCATCA---TTTCACTACTTCATTCGTCTTCATTTCTGTGACATCATCAGCAAGTCTCTTAAT---------GACCTCTACTTCAACGTTTACATC : 1056
BUPS1      : TCTCTCACC---ATAGACCCTAATTTTAACGTCTCCTGGAATTTCCCCTCCAACCCATCG---TTTAACTACCTTATCCGGCTTCATTTCTGTGATATCGTTAGCAAGTCTCTCAAT---------GACCTGTACTTTAATGTGTACATT : 1059

                      *       1220         *       1240         *       1260         *       1280         *       1300         *       1320         *       1340         *
MDS1       : GGATATCAACTGGCCATGCGTGAAATGGACGTGTTTCGGCTG---------AGTGGTGGTTTTCGGCTACCGATGTATCTAGATTTCAAGGTACTTGTCGACGCCGACGGAACTAGCCAGAGACCTAGTCTTCGAGTTGACTTGACACCT : 1146
MDS2       : GAAAATCAAACGGCTACGCTTGAGATGGACGTGTTTCGGATG---------AGTGGTGGTCTTGGATTCCGATGTATCTGGATTATACTGTG---ATTGCGGGTTCGGGAAGTGGTCGGAGACATGATCTAAGGCTTGATTTGCATCCT : 1152
MDS3       : GAAGATAAGATGGCTAAGAAAGAGACGGACGTGATTCGGTTG---------AGCGGTGGTCCTCGGATTCCAATGTATTTAGATTTCAGTGTGTATGTTGGTTTCGAAAGTGGGATGATACAACCTGAGCTACGACTTGACTTGGTTCCT : 1137
MDS4       : GGAAATCAGATGGCTAGGGAAGAGATGGACGTGATTCGGTTG---------AGTGGTGGTCCTCGGATTCCGATTTATCTAGATTTCAGAATATATGTTGGTTCCGAAAGTGGGCCC---AGACCTGATCTACGACTTGACTTGCATCCT : 1137
THE1       : AATGATGATCTTGCTCTTGGAAGTCTTGATCTCTCTACG---TTGACT------AATGGTCTTAAAGTTCCTTACTTTAAGGATTTTATCTCCAATGGTTCTGTTGAATCTTCCGGT---------GTTTTAACCGTTAGCGTTGGACCT : 1104
HERK1      : GACTCAATGGTTGCTGCTACGGATATTGATCTTAGCACTCTTGTGGAT------AACACTTTGGCTGGTGCATATTCGATGGACTTTGTCACGCAGACGCCAAAGGGTAGTAATAAA---------GTC---CGTGTGAGCATCGGTCCG : 1080
HERK2      : AACAACCAGACGGCTGCTGGTAATTTCGATATATTTGCTCACGCGGGC------GGGAAGAACAAAGGTATATAT---CAAGATTACTTG---GATCCGGTCTCCTCTAAGAACGAC---------GTTCTCTGGATTCAACTTGGACCT : 1152
FER        : AACAATCAAACTGCTGAGCCTGAA---------GCTGATGTATTGCTTGGACTAGTTCAAACGGGGTTCCGTTTCACAAGGATTACGTGGTG------AATCCTCCAGAGGGAAATGGACAGCAAGATTTGTGGCTTGCTCTTCATCCT : 1164
ANX1       : AATAACAGAACAGCGCAGGCAGATACTACTCCTGCAGATATAATAGGGTGGACAGGAGAGAAAGGAATCCCGATGTACAAAGACTACGCGATATATGTTGATGCCAACAACGGAGGC------GAAGAGATTACACTTCAGATGACTCCA : 1128
ANX2       : AACAACAGAACCGCGCAAGGGGATACAAACCCCGCAGATATACTCGGGTGGACAGGTGGGAAAGGTATCCCTACTTACAAAGATTACGCGATATATGTTGATGCTAATACCGGAGGAGGAGGGGAAGAGATATCGCTTCAAATGACGCCT : 1137
CVY1       : AACTCAGAGAAA---GTTAGAACCATTGATATGACAGAGGTTTAAAT------GGAACTTTTGGGGCTCCTTTTTCGTTGATGCAGTGATGCGTAAAGCGAAAAGCCGTGAAGGG---------TTCTTGAATTTATCTATTGGTTTA : 1080
ERU-CAP1   : AACAAGCTCTCTGCAATCTCTGCACTTGACCTCTCCTCA---CTAACC------AGCGCTTTAGGTACAGCGTATTACGCGGATGTTCGTGCTTAATGCGTCTACGATCACCAATGGT---------TCGATCTTGGTTCAGGTTGGTCCG : 1122
AT2G23200  : AATGGGTATTGGCGAGTA---GATGTAAAGCCTTCAGAGCAG------------CCTAGGTTGGCGAGTCCGTTTTTTAAGGATGTCGTG---------------AATGTCTCTGATGGTTCTGGACTCTTGAATATCAGTATAGGTACC : 1098
AT5G24010  : AATGAGTATCTTGCGTTTAAGGATGTTGATCTCTCCACGCTTACTTTC------CATGTGCTTGCGTCTCCGTTGTACATTGATTTTGTTGCGGAGTCTGATCGT------TCTGGA---------ATGTTGAGGATTAGTGTTGGACCG : 1113
AT5G59700  : GACTCTATGGATGTTGTTGAGAATCTTGATCTGAGCTCTTATTTATCC------AACACTTTGAGTGGTGCATACGCGATGGATTTTGTCACAGGATCAGCAAAACTGACTAAAAGA---------ATC---CGTGTGAGCATCGGTCGA : 1077
BUPS2      : AATGGAAAGACCGCTATTTCCGGGCTAGATTTGTCCACT---GTAGCT------GGAGATCTTTCTGCTCCTTACTACAAAGACATTGTGGTGAACTCG---ACACTTATGACATCT---------GAGCTTCAGGTCCAGATTGGTCCT : 1185
BUPS1      : AACGGGAAAACTGCCATTTCTGGACTGGATTTGTCCACT---GTCGCT------GGGAATCTAGCTGCTCCTTACTACAAAGACATCGTTGTGAACGCA---ACACTTATGGGCCCT---------GAGCTCCAAGTCCAGATTGGTCCC : 1188
```

```
                1360         *      1380         *      1400         *      1420         *      1440         *      1460         *      1480         *      1500
MDS1       : TACAAAGAGGACTATCCAACCTATTACGACGCTATTTGAGTGGTGTAGAGATTCTCAAG---CTGAGTAATTCTGATGGTAATCTTGCG----------------GGGCTTAATCCAATTCCT------CAACTAAGTCCACCA------ : 1266
MDS2       : TTGGTGAGCATTAATCCGAAATATTATGATGCTATTCTGAATGGTGTGGAGATTCTCAAG---ATGAATGATCCCGATGGTAATCTTGCA----------------GGACCTAATCCAGATCCT------CTAGTATCACCAGACTTAATA : 1278
MDS3       : CTCAAGGACACTAATCAAACGTATTACGACGCTATTCTGAGTGGTGTGGAGATTCTCAAG---CTGAATGATTCTGATGGTAATCTTGCA----------------------AGACCAAATCCAGAACTTCTAGTATCTACGGACTCAACA : 1263
MDS4       : CTGGTGAAGGATAATCCAGAGTATTATGAAGCTATTCTGAATGGTGTAGAGATTCTCAAG---CTAAACAACTCA---GGTAATCTCGCTATCATCCAAGACAATGAACTAAAACCAAATCCTCCACTA------TCATCGAACTTGACA : 1275
THE1       : ---GATTCA---CAAGCTGATATCACTAATGCGACTATGAATGGGTTAGAGGTTTTGAAG---ATTAGTAACGAAGCTAAGAGCTTA---------------AGTGGTGTTTCTTCGGTTAAGTCGTTACTTCCGGGAGGATCAGGTTCT : 1230
HERK1      : ---TCGACTGTTCACACCGATTATCCAAACGCGATTGTGAATGGATTGGAGATTATGAAG---ATGAATAACTCTAAGGGTCAGTTA---------------AGC------------ACTGGGACATTTGTGCCT---GGTAGTAGTTCA : 1194
HERK2      : GACTCATCTGTTGGTGCTTCT---GGAGACGCTCTTCTGAGTGGTCTTGAGATTTTCAAG---CTCAGCAAAAAT---GGGAATCTT---------------GCTCATCTCATCAGGTTTGATTCGACTGGTCACTCGGTAAGTGACTCG : 1278
FER        : AACCCAGTTAACAAGCCGGAGTATTATGATTCTCTTCTTAATGGAGTGGAGATATTCAAG---ATGAATACTTCTGATGGTAATCTG---------------GCTGGTACCAATCCTATACCTGGTCCACAGGTGACTGCTGATCCATCT : 1296
ANX1       : TCAACTTTTGGTCAACCGGAATATTATGATTCATCTCTTAACGGGTTAGAGATTTTCAAG---ATGGACACCATGAAA---AATCTT---------------GCGGGTCCAAACCCCGAGCCATCGCCTATG---------CAAGCTGAG : 1248
ANX2       : TCGACATTTGGTCAACCGGAATATTACGATTCACAGCTTAATGGGCTAGAGATTTTCAAG---ATTGACACAATGAAG---AACCTT---------------GCCGGTCCAAACCCTAAGCCATCACCTATG---------CAAGCTAAT : 1257
CVY1       : GTTATGGATGTT---TCTTCGTACCCTGTTTCTTTTATTAACGGATTTGAGATTTCGAAG---CTAAGCAATGATAAGCGGAGTCTT---------------GATGCTTTTGATGCCATT------TTACCTGATGGTTCTTCGAGTAAC : 1203
ERU-CAP1   : ACTCCGAATCTTCAGTCGGGTAAACCGAATGCGATACTTAACGGTTTAGAGATCATGAAG---CTGAACAATGCTGCTGGGAGTTTA---------------GATGGACTTTTTGGAGTTGATGGGAAATACAAAGGACCGATCGGTGGT : 1254
AT2G23200  : AAG---------GAGGCCAATAAGGATGCTGGTTTTCTGAATGGTCTGGAGATGATGGAGGTTTTGAGTAAATCTGGTTCTGATTAT---------------TCAAATAGAAGCAGTTCCCGGGTTCACATCATCACTGGTTGT------ : 1218
AT5G24010  : TCTGATCTTAGTAATCCGGCGAGAGTTAATGCTCTATTGAATGGAGTTGAGATCATGAGG---ATT-------------------------------------TTGAGCCCTGTGAGTTCTGAAGTGGTATCTGGAAAGAGGAAT : 1218
AT5G59700  : ---TCGAGTGTTCACACGGATTACCCTACTGCGATTCTGAACGGATTAGAGATCATGAAG---ATGAATAACTCCAAAAGTCAGCTC---------------AGT------------ATTGGAACATTTTTGCCCAGTGGTTCAAGTTCA : 1194
BUPS2      : ATGGGAGAA---GACACTGGGAAAAAGAACGCGATCTTGAACGGTGTCGAGGTCTTGAAG---ATGAGCAACTCTGTGAACAGTCTT---------------GATGGAGAATTTGGAGTGGATGGTCAA------------AGAGCGAGT : 1302
BUPS1      : ATGGGAGAA---GATACAGGGACCAAAAACGCGATCTTGAACGGTGTTGAGGTTCTGAAG---ATGAGCAATTCCGTGAACAGCCTT---------------GATGGAGAGTTTGGAGTGGATGGTAGA------------ACCACTGGC : 1305

                   *      1520         *      1540         *      1560         *      1580         *      1600         *      1620         *      1640         *
MDS1       : CCACAATCTATAACG---CCACTAAAAGGAAAGGCAAGTCATCACATGTTTTGCCAATAATAATTGCAGTGGTTGGTTCTGCAGTTGCGCTAGCGTTTTTTGTTCTTGTTGTTGTCCTCGTTGTT------ATGAAGAGAAAGAAGAAG : 1407
MDS2       : CCAAACCGTGCAACG---CCG---CGGATTCGAAAAAATAAGTCACATATTTTGCCGATCACACTTGCAGTGGTTGGTTCTTTAGTTGTGCTAGCAATGTTTGTT---GTTGGTGTTCTTGTTATC------ATGAAGAAGAAGAAGAAG : 1413
MDS3       : CCAGATGATTCTAATGTTACACCACCAATAAAAGGTAAGCCA---CATGTTTTGGTGATAATCCTCATAGTCGTTGGTTCTGTAATTGGGCTAGCGACTTTCATTGTTATCATCATGTTGTTGATCCGTCAGATGAAGAGGAAGAAGAAT : 1410
MDS4       : CCAAAC------CATGTAACGCAGCAGATCAAAGGTAAGTCTTCACATCTTCTGGTGAAAATCTTCATAGCCGTTGGTCCCGGAACTGGGCTGGCGACTTTCGTTGTTGTTCTCATGTTGTGGATGCGTCAGATGAAGAGGAAG---AAT : 1416
THE1       : AAG---------------------AGCAAGAAGAAG------GCAGTGATCATTGGTTCTTTGGTTGGTGCGGTTACATTGATTCTGCTGATTGCTGTTTGTTGCTATTGCTGTTTGGTTGCTTCAAGGAAGCAGAGGTCGACGAGTCCT : 1353
HERK1      : AGC---------------------AGTAAGAGTAAT---CTCGGGTTGATTGTAGGTTCAGCCATTGGTTCGTTG---CTCGCGGTAGTCTTCTTGGGAAGTTGCTTT------GTGTTGTATAAGAAGCGGAAACGTGGC--------- : 1302
HERK2      : AAG---------------------ATGCGGATTATTTGGATCAGTGTTGGTGCTGGTATAGCAATTATCATTTTTTTCGTGTTCTTGGGAATCTTGGTAGTATGTTTATGC---------------AAGAAAAGGCGAAGCAAATCAGAT : 1392
FER        : AAAGTCCTACGCCCGACTACTAGGAAATCGAAAAGCAATACGGCTATTATTGCAGGCGCAGCCAGTGGTGCAGTTGTTCTGGCCCTTATCATTGGGTTTTGTGTGTTT------GGTGCTTACCGCAGACGTAAGCGTGGTGATTACCAG : 1440
ANX1       : GAAGAGGTTAAAAAAGAATTCAAGAACGAAAAGAGA---CACGCTTTCATCATTGGTTCGGCC---GGAGGAGTTTTAGCGGTTTTAATCGGTGCCTTGTGT---TTC------ACGGCCTACAAGAAGAAACAA------GGATATCAA : 1377
ANX2       : GAAGATGTTAAAAAGGACTTTCAAGGTGACAAAAGAATCACAGCTTTTGTCATTGGTTCTGCT---GGAGGAGTAGCAGCTGTTTTATTTTGTGCATTGTGT---TTC------ACAATGTACCAGAGAAAAAGA------AAGTTTTCG : 1389
CVY1       : AAG---------------------AGTTCGAATACGAGTGTCGGATTGATAGCTGGTTTGTCTGCAGCTTTGTGTGTTGCTTTAGTGTTTGGTGTGGTTGTGTCTTGGTGGTGCATT---------AGGAAAAGAAGAAGAAGAAATAGA : 1323
ERU-CAP1   : ATG---------------------TCATCTAAGAAG---CTTGCTATTGCGGGTATTGGGTTTGTGATGGCGCTAACCGCTTTCTTAGGGGTTGTCGTG---------------TTGCTTGTTAGATGGCAGAGACGTCCTAAAGACTGG : 1365
AT2G23200  : ------------------------------------------GCTGTTGCTGCTGCTGCTGCATCAGCTTTGGTCTTCAGTTTGCTGTTT-------------------------ATGGTATTCTTGAAGCGGAGGAGATCGAAGAAGACT : 1302
AT5G24010  : GTT---------------------GTCTGGATCGTG---GTTGGCTCGGTTTTGGGAGGTTTTGTGTTCTTGTCGCTGTTCTTCTTGAGCGTTTTG------TGCTTGTGC---------------------AGGCGCAAGAACAACAAG : 1317
AT5G59700  : ACC---------------------ACTAAGAAGAAT---GTTGGTATGATCATTGGTTTAACGATCGGCTCATTG---CTTGCTTTAGTGGTTTTAGGAGGTTTCTTT------GTGCTGTACAAGAAACGAGGACGAGAC--------- : 1302
BUPS2      : ATG---------------------GGGAAGCAGGGG---ATG---GTTGCAACCGCGGGTTTTGTGATGATGTTTGGAGCCTTCGTAGGTCTTGGAGCA---------------ATGGTTTACAAGTGGAAGAAGAGACCACAAGATTGG : 1410
BUPS1      : ATG---------------------GGGAAGCATGGT---ATG---GTAGCAACCGCGGGGTTTGTGATGATGTTTGGAGCTTTCATTGGTCTGGGAGCC---------------ATGGTTTACAAGTGGAAGAAGAGGCCACAAGATTGG : 1413

                  1660         *      1680         *      1700         *      1720         *      1740         *      1760         *      1780         *      1800
MDS1       : AGCAACGAGTCTAGTGTAGATACCACGAACAAGCCTTCTACGAACTCGTCATGGGGTCCTCTTCTGCACGGGACAGGCTCTACAAATACAAAATCTGCCTCATCTCTT------------------------------------------ : 1515
MDS2       : AGC--------------------------AAGCCATCTACAAACTCGTCATGGTGTCCTCTTCCGCACGGGACAGATTCTACGAATACAAAACCAGCGAAATCTCTG------------------------------------------ : 1494
MDS3       : AAAAAGGAGAATAGTGTCATTATGTTCAAGTTA-------------------------------------------------------------------------------------------------------------------------- : 1443
MDS4       : AGGAAGGAAGAAAGGGTCGTCATGTTCAAGAAA-------------------------------------------------------------------------------------------------------------------------- : 1449
THE1       : CAAGAAGGCGGTAATGGACATCCGTGGTTGCCATTACCTTTATATGGACTCTCTCAGACTCTTACTAAATCAACCGCTTCTCACAAGAGTGCCACAGCTAGTTGCATTTCATTAGCTTCTACTCAT------------------------ : 1479
HERK1      : ---CAAGACGGTCATTCAAAGACTTGGATGCCGTTTTCGATAAATGGA---------------------ACTTCGATGGGAAGCAAATACTCCAATGGAACTACGCTTACAAGTATAACTACCAAT------------------------ : 1404
HERK2      : GAGTCGAAAAACAATCCTCCCGGGTGGCGTCCGTTGTTTTTGCATGTCAATAACAGTACT---GCAAACGCCAAAGCAACGGGAGGCTCGCTGAGACTGAACACTCTTGCAGCATCTACA------------------------------ : 1509
FER        : CCTGCTAGTGATGCAACATCAGGGTGGCTTCCACTATCTCTGTATGGAAACTCACATTCTGCTGGCTCGGCGAAG----------------------------------ACA------------AACACAACAGGAAGTTATGCCTCGTCC : 1545
ANX1       : GGAGGCGACTCTCACACATCAAGTTGGCTTCCT------ATATATGGAAACTCTACCACATCAGGAACCAAATCA----------------------------------ACCATCTCAGGGAAGAGCAACAATGGAAGTCACCTATCCAAT : 1488
ANX2       : GGCAGCGATTCTCACACATCAAGCTGGCTTCCT------ATATATGGAAACTCACACACATCTGCAACAAAATCT----------------------------------ACTATATCGGGTAAGAGCAACAACGGAAGCCACCTATCGAAT : 1500
CVY1       : CAAATGCAGACT---------------------GTTCATTCTAGAGGAGATGATCATCAGATTAAGAAGAATGAAACAGGTGAGAGC------------------------------------------------------TTAATCTTT : 1398
ERU-CAP1   : CAAAAACAGAATAGTTTCTCGTCATGGTTG---CTTCCTTTACACGCAAGTCACTCGAGTTACATCTCGAGCAAAGGCGGGTCAACGTCGAGGCGGATGAGTATTTTCGGGTCAAAGAAGAGTAAGAGCAATGGATTCTCTAGCTTCTTC : 1512
AT2G23200  : AAGCCAGAGGTTGAGGGCACTGTATGGTCTCCTTTGCCATTGCACAGAGGTGGGAGTTCAGACAATAGACCTATCTCTCAATACCACAACTCCCCATTACGCAACCTACAC---------------------------------------- : 1413
AT5G24010  : ACGAGGAGTTCAGAGAGCACGGGATGGACACCTTTG------------------AGGAGGTTTAGAGGCAGTTCCAATAGTAGAACA---------------------ACTGAAAGAACTGTTAGTTCTAGTGGCTACCATACTCTG--- : 1425
AT5G59700  : ---CAAGATGGTAATTCAAAGACTTGGATTCCTTTGTCATCCAATGGA---------------------ACAACTTCATCCTCA---------AATGGAACTACACTTGCAAGTATAGCTTCCAAT------------------------ : 1395
BUPS2      : CAGAAGAGAAACAGTTTCTCTTCTTGGTTA---CTTCCAATACACGCTGGTGACAGTACCTTCATGACAAGCAAAACAGGTTCTCACAAATCCAACTTG--------------------------------------------TACAAT : 1512
BUPS1      : CAGAAGAGAAATAGTTTCTCCTCTTGGTTG---CTACCAATACATGCTGGTGATAGCACTTTCATGACAAGCAAAGGTGGTTCTCAGAAATCCAACTTC--------------------------------------------TACAAC : 1515
```

```
                *      1820         *      1840         *      1860         *      1880         *      1900         *      1920         *      1940         *
MDS1      : ---CCATCAGATCTCTGCCGTCGATTCTCCATCTACGAAATCAAATCCGCCACAAATGATTTCGAGGAAAAACTAATCATAGGAGTAGGCGGGTTTGGTTCTGTCTACAAAGGACGAATAGACGGTGGAGCCACACTTGTTGCGGTTAAA : 1662
MDS2      : ---CCCGCGGATCTCTGTCGTCGATTCTCCATTTCGAAATCAAATCCGCCACAAATGATTTCGAGGACAAGCTAATCATCGGAGTAGGCGGGTTTGGTTCTGTCTACAAAGGACAAATAGACGGTGGAGCCACACTTGTTGCGGTTAAA : 1641
MDS3      : ------------CTACTGAAACAGTACATTTATGCAGAATTGAAGAAGATTACAAAGTCGTTTTCCCAC------ACTGTTGGAAAGGAGGGTTTGGAACTGTATATAGAGGAAACCTTTCTAATGGACGTACG---GTTGCAGTAAAG : 1572
MDS4      : ------------CTACTGAATATGTACACTTATGCAGAACTAAAGAAAATTACAAAGTCATTTTCTTAC------ATAATTGGAAAGGAGGATTTGGAACTGTTTATGGAGGAAACCTTAGTAATGGTCGTAAG---GTTGCAGTAAAA : 1578
THE1      : ------------CTTGGACGTTGCTTTATGTTTCAAGAAATCATGGACGCTACTAATAAGTTCGATGAGAGTTCGTTGCTTGGGGTTGGTGGATTTGGCCGCGTTTATAAAGGAGAACTTTAGAAGACGGGACT---AAAGTCGCGGTTAAA : 1614
HERK1     : ------------GCCAATTACCGTATTCCCTTTGCACCGGTTAAAGACGCTACAAATAACTTCGACGAGAGCCGCAACATCGGTGTAGGCGGTTTCGGTAAAGTCTACAAAGGAGAGCTTAATGACGGTACA---AAGGTAGCTGTGAAA : 1539
HERK2     : ------------ATGGGAAGGAAGTTTACACTAGCCGAGATTCGTGCAGCAACTAAGAACTTTGATGATGGTTTAGCTATTGGAGTTGGAGGGTTTGGTAAGGTTTACAGAGGAGAGCTTGAACATGGAACA---CTCATAGCTATAAAA : 1644
FER       : CTTCCGTCAAATCTTTGCCGTCACTTCTCGTTTGCTGAGATCAAAGCTGCCACTAAAAACTTTGATGAGTCCCGGGTGCTTGGTGTTGGTGGTTTCGGCAAGGTTTACAGAGGAGAGATTGATGGCGGAACTACAAAGGTAGCCATCAAG : 1695
ANX1      : CTCGCAGCAGGTTTATGCCGTAGATTCTCTTTACCAGAAATCAAACACGGAACACAAAACTTCGATGATTCCAACGTCATTGGAGTTGGAGGGTTTGGTAAAGTCTACAAAGGAGTTATAGACGGCACAACT---AAAGTAGCGGTAAAA : 1635
ANX2      : CTTGCAGCGGGTTTATGTCGAAGATTCTCATTGTCCGAAATAAAACACGGAACTCATAACTTCGATGAGTCAAACGTAATCGGAGTAGGAGGGTTTGGTAAGGTTTACAAAGGAGTTATAGATGGAGGCACA---AAAGTGGCAATCAAG : 1647
CVY1      : TCGAGCTCAAAGATCGGTTATCGATACCCTTTGGCTCTAATCAAGGAAGCAACTGATGATTTTGATGAAAGCTTAGTGATTGGGGTTGGTGGGTTTGGTAAAGTTTACAAAGGAGTCTTGAGACACAAAACC---GAAGTAGCTGTGAAA : 1545
ERU-CAP1  : TCAAACCAGGGATTAGGTCGGTACTTTCGCTTTACCGAACTACAGACCGCGACACAAAATTTTGACGAGAATGCTGTGTGTGGAGTCGGAGGATTCGGAAAAGTCTACATAGGAGAAATTGATGGTGGACG---CAGGTCGCGATCAAA : 1659
AT2G23200 : ------------TTAGGCTTAACGATTCCCTTCACAGACATTCTAAGCGCTACAAACAACTTCGACGAGCAGTTGTTGATCGGGAAAGGCGGGTTTGGCTACGTTTACAAAGCTATTCTTCCCCATGGAACC---AAAGCCGCTATCAAA : 1548
AT5G24010 : ---------------------AGAATCTGTTCGCGGAGTTACAATCCGGAACCAATAACTTTGATAGAAGTTTAGTTATCGGAGTTGGCGGATTCGGGATGGTTTTTAGAGGTCTCTTAAAGACAACACC---AAAGTAGCTGTGAAG : 1551
AT5G59700 : ------------TCCAGTTATCGAATTCCTTTGGTAGCAGTTAAAGAGGCCACAAATAGCTTTGATGAGAATCGAGCAATTGGAGTTGGTGGTTTCGGTAAAGTGTACAAAGGAGAGCTTCACGACGGTACA---AAAGTAGCTGTTAAA : 1530
BUPS2     : TCCGCGCTTGGCCTCGGACGCTATTTCTCCCTCTCTGAGCTTCAAGAAGTCACAAAGAACTTCGATGCATCAGAGATCATCGGTGTTGGTGGATTTGGTAACGTTTACATTGGTACCATTGACGATGGAACT---CAAGTTGCTATCAAA : 1659
BUPS1     : TCAACACTTGGTCTCGGCCGCTATTTCTCCCTTTCAGAGCTTCAAGAAGCCACAAAGAACTTTGAAGCATCCCAGATTATCGGTGTTGGAGGATTCGGTAACGTCTACATTGGAACCCTCGACGATGGAACC---AAAGTCGCTGTCAAA : 1662

                   1960         *      1980         *      2000         *      2020         *      2040         *      2060         *      2080         *      2100
MDS1      : CGGCTGGAAATTACATCGAACCAAGGTGCTAAAGAGTTCGATACAGAGCTCGAGATGCTTTCAAAGCTTCGACATGTACACCTCGTCTCTCTAATCGGATATTGCGATGACGACAACGAGATGGTACTTGTCTATGAGTATATGCCACAT : 1812
MDS2      : CGGCTGGAAATTACATCGAACCAAGGTGCTAAAGAGTTCGAAACAGAGCTCGAGATGCTCTCAAAGCTTCGACATGTACATCTCGTCTCTCTAATCGGCTACTGCGACGAGGACAACGAGATGGTACTAGTCTATGAGTATATGCCACAT : 1791
MDS3      : GTCTTGAAG---GATTTGAAGGGAAACGGTGACGATTTCATCAACGAAGTCACCAGCATGAGCCAAACATCTCATGTAAACATTGTTTCTCTGCTAGGTTTTGCTATGAAGGGTCCAAAAGAGCGATTATATCAGAGTTTCTGGAGCAT : 1719
MDS4      : GTTTTGAAA---GATTTGAAGGGAAGTGCTGAAGATTTCATCAATGAAGTTGCAAGCATGAGCCAAACATCTCATGTGAACATTGTTTCTCTTCTTGGTTTCTGCTTCGAAGGGTCTAAGAGAGCCATTGTGTATGAGTTTTGGAGAAT : 1725
THE1      : AGAGGTAACCCGAGATCAGAACAAGGTATGGCTGAGTTCAGAACAGAGATTGAAATGCTGTCAAAACTCAGACATCGACATCTCGTCTCTCTTATCGGTTACTGCGACGAGAGGTCTGAAATCATACTGGTCTATGAGTACATGGCGAAT : 1764
HERK1     : AGAGGGAACCCAAAATCTCAGCAAGGGCTTGCGGAATTCAGGACAGAAATCGAGATGTTATCAGTTTCGTCACCGCCATTTGGTTTCTCTGATCGGTTATTGTGACGAGAACAATGAGATCATACTAATTTACGAGTATATGGAGAAT : 1689
HERK2     : CGAGCCACCCCACATTCTCAGCAAGGGCTTGCTGAATTCGAAACCGAGATCGTGATGCTCTCAAGACTTAGGCATAGGCATCTTGTGTCTCTTGATCGGGTTCTGCGATGAGCACAATGAGATGATCTTGGTTTATGAATACATGGCAAAT : 1794
FER       : AGAGGCAACCCAATGTCCGAGCAAGGTGTACATGAGTTCCAGACTGAGATTGAAATGCTTTCAAAGCTTAGACATCGTCATCTTGTGTCCTTGATTGGATACTGTGAAGAGAACTGCGAAATCATCTTAGTCTATGATTACATGGCTCAT : 1845
ANX1      : AAATCAAACCCAAATTCAGAACAAGGACTCAACGAATTCGAGACAGAAATCGAACTCCTCTCAAGACTAAGACACAAACACTTAGTCTCCTTGATAGGATACTGCGACGAGGGAGGAGAAATGTGTCTCGTATACGATTACATGGCATTT : 1785
ANX2      : AAATCAAACCCTAATTCAGAACAAGGCTCAACGAATTCGAGACAGAAATCGAACTTCTCTCAAGATTAAGACACAAACACTTAGTCTCATTAATTGGATACTGCGATGAAGGTGGAGAAATGTGTCTAATATACGATTACATGTCTCTT : 1797
CVY1      : CGTGGTGCTCCTCAATCTCGACAAGGTCTGGCGGAGTTTAAGACAGAGGTTGAAATGCTTACTCAGTTCAGACACAGACACTTGGTTTCTTTAATCGGATATTGCGATGAAAACAGTGAAATCATCATTGTCTATGAATATATGGAGAAG : 1695
ERU-CAP1  : AGAGGGAGTCAGAGTTCGGAGCAAGGAATCAACGAGTTCAAACAGAGATTCAGATGTTGTCCAAACTCAGACATAGACATTTGGTTTCTCTAATCGGATTTGCGATGAAAACAAAGAGATCATTCTCGTCTACGAATACATGTCAAAC : 1809
AT2G23200 : CGAGGCAAAACCGGCTCAGGACAAGGGATCTTAGAGTTTCAAACCGAGATTCAAGTCCTCTCAAGAATCCGACATAGACACCTCGTCTCGCTAACGGGATACTGCGAAGAAAACTCCGAAATCATCCTCGTCTACGAGTTTATGGAGAAA : 1698
AT5G24010 : AGAGGCTCTCCTGGTTCAAGACAGGGCTTACCAGAGTTTCTCTCTGAGATAACCATTCTCTCTAAGATCCGTCATCGCCATCTAGTTTCACTTGTTGGGTATTGCGAAGAACAGTCTGAGATCATTCTTGTTTATGAGTACATGGATAAA : 1701
AT5G59700 : AGAGCGAATCCTAAGTCTCAGCAAGGACTTGCAGAGTTCAGGACAGAAATCGAAATGTTGTCACAATTCCGTCATCGCCATTTGGTTTCTTTGATCGGTTACTGCGACGAGAATAACGAGATCATTCTGGTTTACGAATATATGGAGAAT : 1680
BUPS2     : CGAGGCAATCCACAGTCAGAGCAAGGGATCACAGAGTTCATACAGAGATTCAGATGCTATCTAAGCTTAGACACAGACACTTAGTCTCATTGATTGGATACTGCGATGAGAACGCAGAGATCATCCTTGTATACGAATACATGTCCAAT : 1809
BUPS1     : AGAGGTAATCCACAGTCTGAACAAGGAATCACTGAGTTTCAGACAGAGATCCAGATGCTTTCTAAGCTCAGACACAGACACTTGGTCTCCTTGATTGGTTACTGTGACGAGAACTCTGAGATCATCTTAGTCTATGAGTTCATGTCCAAT : 1812

                         *      2120         *      2140         *      2160         *      2180         *      2200         *      2220         *      2240         *
MDS1      : GGTACACTTAAAGATCATCTTTTC---------AGGAGAGACAAGGCCTCTGATCCTCCATTGTCATGGAAACGAAGGCTAGAGATTTGCATTGGAGCAGCTCGTGGATTACAGTATCTTCATACT---GGAGCCAAGTACACGATCATA : 1950
MDS2      : GGTACACTTAAAGATCATCTTTTC---------AGGAGGGACAAAACCTCTGATCCTCCATTGTCATGGAAACGAAGGCTAGAGATTTGCATTGGAGCAGCTCGTGGATTACAGTATCTTCATACT---GGAGCCAAGTACACGATCATA : 1929
MDS3      : GGGTCTTTG---GATCAGTTCATC---------TCTAGAAATAAATCATTGACTCGGAACGTGACA------ACACTATATGGAATCGCGCTAGGCATCGCTCGGGGATTGGAGTATTTGCACTAT---GGCTGCAAAACAAGAATTGTG : 1848
MDS4      : GGGTCTCTC---GATCAGTTCATG---------TCTAGAAATAAGTCATTGACACAGGATGTAACA------ACACTATATGGAATCGCGCTAGGCATCGCTCGGGGATTGGAGTATTTGCATTAT---GGCTGCAAAACAAGAATTGTG : 1854
THE1      : GGACCGTTGAGGAGTCATCTATATGGAGCTGAT------------------CTTCCTCCATTGTCTTGGAAACAAAGACTCGAGATTTGCATCGGTGCAGGAGAGGATTACATTATCTACACACC---GGTGCATCGCAGAGCATTATA : 1893
HERK1     : GGAACGGTAAAGAGTCATCTTTATGGCTCAGGT------------------CTACCTAGCTTGACTTGGAAACAACGGCTTGAGATCTGCATTGGTGCAGCTAGAGGCTTGCATTACCTTCACACG---GGTGACTCGAAACCGGTCATT : 1818
HERK2     : GGAACTCTCAGGAGTCATCTCTTTGGAAGCAAC------------------CTTCCGCCATTATCATGGAAGCAACGGCTTGAAGCTTGTATAGGCTCTGGGAGAGGATTGCATTACCTTCACACA---GGGTCAGAGAGAGGAATCATT : 1923
FER       : GGTACAATGAGGGAGCATCTCTACAAAACCCAGAAT------------------CCTTCTCTTCCATGGAAGCAACGGCTTGAGATATGCATTGGAGCAGCCCGAGGTTTACACTATCTACACACT---GGTGCAAAACACACAATCATC : 1974
ANX1      : GGAACACTCCGTGAGCATCTATACAACACAAAGAAA------------------CCACAATTAACTTGGAAACGAAGGCTAGAGATAGCTATTGGAGCAGCAAGAGGATTACATTACCTTCACACA---GGAGCAAAGTACACGATTATA : 1914
ANX2      : GGAACACTCAGAGAACATCTTTACAACACAAAGAGA------------------CCTCAGTTAACTTGGAAACGAAGACTCGAAATCGCCATTGGAGCTGCAAGAGGATTACATTACCTACACACA---GGAGCAAAGTACACTATCATT : 1926
CVY1      : GGAACGCTTAAGGACCATTTGTATGACTTGGATGATAAG---------------CCGAGGTTGAGTTGGAGACAGAGGCTTGAGATATGCGTTGGTGCAGCTAGAGGGCTTCATTATCTCCACACA---GGTTCCACAAGAGCAATCATA : 1827
ERU-CAP1  : GGTCCTCTGCGTGACCATCTCTACGGCTCCAAAGAAAATGACCCTAACCCTATTCCTACCTTGTCCTGGAAGCAACGTCTAGAGATCTGCATTGGATCAGCGCGTGGACTCCATCATCTCCACACG---GGTGCAGCGCAGGGGATCATC : 1956
AT2G23200 : GGTACACTCAAAGAACATCTATACGGCTCGAAT------------------CTACCTCATTAACATGGAAACAAAGACTAGAGATATGCATTGGAGCAGCTAGAGGTTTAGATTATCTCCACAGTAGTGGCTCAGAAGGAGCAATCATT : 1830
AT5G24010 : GGACCACTCAAAAGTCACTTATACGGATCCACCAAC------------------CGGCCTTTATCTTGGAAACAACGGCTTGAGGTCTGCATTGGTGCAGCCAGAGGACTTCATTACCTTCACACC---GGTTCTTCGCAAGGAATCATC : 1830
AT5G59700 : GGAACATTGAAGAGTCACCTTTACGGTTCAGGT------------------CTTCTTAGCTTGAGTTGGAAGCAACGTCTTGAGATATGCATTGGATCAGCAAGAGGATTGCATTATCTCCACACC---GGTGACGCGAAACCCGTGATT : 1809
BUPS2     : GGTCCTTTTAGAGATCATCTTTATGGGAAGAAT------------------CTTTCTCCTTTGACTTGGAAACAAAGACTGGAGATATCTGCATTGGAGCTGGTCGTGGACTTCACTATTTACACACA---GGAACAGCTCAAGGCATTATC : 1938
BUPS1     : GGTCCATTTCAGGGACCACTTGTATGGAAAGAAC------------------CTTGCTCCTCTAACCTGGAAACAAAGACTCGAGATATGTATCGGATCTGCACGTGGCTTCACTACTTACACACA---GGAACAGCTCAGGGGATTATC : 1941
```

```
                         2260         *        2280         *        2300         *        2320         *        2340         *        2360         *        2380         *        2400
MDS1      : CATAGAGACATCAAAACCACAAACATACTTCTCGATGAGAACTTCGTCGCTAAAGTATCTGACTTTGGTTTATCAAGAGTTGGTCCTACTAGTGCTTCTCAAACGCATGTCTCCACCGTCGTTAAAGGAACGTTTGGTTACTTGGATCCC : 2100
MDS2      : CATAGAGACATCAAAACCACAAACATACTTCTCGATGAGAACTTCGTCACTAAAGTATCTGACTTTGGTTTATCAAGAGTTGGTCCTACTAGTGCTTCTCAAACGCATGTCTCCACCGTCGTTAAAGGAACGTTTGGTTACTTGGACCCC : 2079
MDS3      : CATTTCGATATTAAACCTCAAAATATTCTGCTCGATGACAATTTTTGTCCTAAAGTCGCCGACTTTGGCCTTGCTAAGCTTTGTGAGAAAAGAGAAAGCATTTTGTCATTAATAGACACA---AGAGGAACTATAGGTTACATTGCACCA : 1995
MDS4      : CATTTCGACATTAAACCTCAGAATATTCTGTTAGATGGCAATCTTTGTCCTAAAGTCTCAGACTTTGGCCTTGCTAAGCTTTGTGAGAAAAGAGAAAGTGTATTGTCATTGATGGACACA---AGAGGAACTATAGGTTACATTGCACCA : 2001
THE1      : CACCGTGATGTTAAAACCACGAATATCTTACTCGACGAGAATCTAGTCGCCAAAGTTGCAGACTTTGGACTATCCAAAACCGGCCCTTCGCTCGAT---CAAACACACGTGAGCACGGCGGTTAAAGGAAGCTTTGGTTATCTAGACCCG : 2040
HERK1     : CACAGAGACGTGAAATCTGCAAACATATTGCTTGACGAGAACTTCATGGCTAAAGTTGCAGACTTTGGACTGTCCAAGACTGGACCCGAGCTTGAT---CAGACTCATGTAAGTACTGCTGTCAAAGGAAGTTTTGGTTATCTTGACCCC : 1965
HERK2     : CACAGAGATGTCAAAACAACAAACATACTATTAGACGAGAACTTTGTGGCAAAGATGTCTGATTTTGGGCTGTCGAAAGCTGGACCGTCCATGGAC---CATACTCATGTGAGTACAGCTGTGAAAGGAAGTTTTGGTTATCTTGATCCT : 2070
FER       : CATAGAGATGTGAAGACAACAAACATTCTATTGGATGAGAAATGGGTGGCCAAGGTCTCTGATTTTGGTCTATCGAAGACTGGTCCTACACTAGAC---CACACACACGTAAGCACAGTTGTGAAAGGAAGTTTCGGTTATCTTGACCCA : 2121
ANX1      : CACAGAGACGTTAAAACAACTAACATCCTCGTAGATGAGAACTGGGTAGCCAAAGTTTCAGACTTTGGGTTATCCAAAACTGGACCTAACATGAAT---GGAGGCCATGTAACAACCGTCGTCAAAGGAAGCTTCGGTTATTTAGATCCT : 2061
ANX2      : CACCGTGACGTCAAAACAACTAACATCTTACTAGATGAGAATTGGGTTGCTAAAGTTTCAGATTTCGGATTGTCCAAAACCGGACCAAACATGAAT---GGTGGTCATGTCACAACCGTTGTGAAAGGAAGTTTCGGTTACTTGGATCCA : 2073
CVY1      : CACCGCGATGTGAAATCTGCTAACATTCTATTGGATGATAATTTTATGGCTAAAGTTGCAGATTTTGGATTGTCTAAGACAGGTCCTGATCTTGAT---CAAACACATGTGAGTACCGCGGTCAAAGGAAGCTTTGGATATCTTGATCCA : 1974
ERU-CAP1  : CACCGCGACGTCAAGACCACCAACATCCTCTTGGACGAGAACCTAGTCGCTAAAGTTTCTGATTTTGGACTTTCAAAAGATGCGCCT---ATGGAT---GAAGGGCATGTAAGTACCGCGGTAAAGGTAGTTTCGGGTATTTAGACCCC : 2100
AT2G23200 : CACAGAGACGTCAAATCAACAAACATATTACTAGACGAGCACAATATAGCAAAGTCGCAGATTTCGGATTATCGAAAATT---CATAATCAAGAT---GAAAGCAATATCAGCATAAACATCAAAGGAACATTCGGTTACTTGGATCCA : 1974
AT5G24010 : CACCGGGATATCAAATCCACCAATATTCTTACTAGATAACAACTATGTAGCCAAAGTTGCAGACTTTGGACTCTCAAGATCCGGTCCTTGTATTGAT---GAGACACATGTGAGCACCGCGTGTAAAGGAAGCTTTGGATATCTTGATCCT : 1977
AT5G59700 : CACAGAGACGTCAAATCCGCCAACATATTGCTTGACGAGAATCTCATGGCTAAAGTTGCAGACTTTGGACTGTCTAAGACCGGACCGGAGATCGAT---CAGACTCATGTGAGTACCGCGGTCAAAGGAAGTTTCGGATATCTTGATCCA : 1956
BUPS2     : CATCGTGACGTCAAATCCACAAATATTCTTCTTGATGAAGCTTTAGTTGCAAAAGTTGCTGACTTTGGTCTCTCCAAAGACGTTGCC---TTTGGA---CAAAACCATGTTAGTACGGCTGTGAAAGGTAGTTTTGGGTATTTAGACCCG : 2082
BUPS1     : CACCGTGATGTCAAATCCACCAACATTCTTCTCGATGAAGCTTTAGTTGCAAAAGTCGCTGACTTTGGTCTTTCTAAAGATGTAGCT---TTTGGA---CAAAACCATGTCAGCACGGCCGTGAAAGGAAGTTTCGGGTACTTAGACCCG : 2085

                        *        2420         *        2440         *        2460         *        2480         *        2500         *        2520         *        2540         *
MDS1      : GAGTACTATCGCCGTCAAATC------TTGACCGAAAAATCCGACGTGTACTCTTTTGGAGTTGTTCTGTTGGAGGTTTTGTGCTGTAGACCGATCAGAATGCAAAGTGTTCCACCGGAACAAGCAGAT---------TTGATCCGATGG : 2235
MDS2      : GAGTACTATCGCCGTCAAGTC------TTGACCGAAAATCTGACGTGTACTCTTTTGGAGTTGTTCTGTTGGAGGTTTTGTGTTGTAGACCGATTAGAATGCAAAGTGTTCCACCGTGGAACAACCAGAT---------TTGATCCGATGG : 2214
MDS3      : GAGGTGGTCTCAAGAATGTACGGTGGAATCTCCCATAAATCTGATGTGTATAGCTATGGAATGTTGGTCCTCGACATGATAGGACCAAGAAATAAA------GTTGAAACCACAACTTGCAATGGTAGTACAGCCTATTTTCCTGATTGG : 2139
MDS4      : GAGGTCTTCTCAAGAATGTATGGCAGAGTCTCACATAAGTCTGATGTGTATAGCTTTGGAATGCTTGTCATCGACATGATTGGACCAAGGAGCAAAGAAATAGTAGAAACTGTTGATTCCGCCGCTAGTTCAACTTACTTTCCAGATTGG : 2151
THE1      : GAATACTTCAGGAGACAG------CAGTTAACAGAGAAATCAGACGTTTATTCGTTTGGTGTTGTACTAATGGAAGTACTCTGTTGTAGACCGGCTTTAAACCCGGTATTACCTAGAGAACAACTGAAC---------ATAGCGGAATGG : 2175
HERK1     : GAGTACTTCAGAAGGCAA------CAGCTCACAGATAAATCCGATGTTTATTCCTTCGGAGTTGTTCTCTTCGAGGTTTTATGTGCTAGACCTGTTATAGACCCAACACTCCCAAGAGAGATGGTGAAT---------CTTGCAGAATGG : 2100
HERK2     : GAATACTTTAGAAGGCAA------CAGTTAACAGAGAAGTCAGATGTTTACTCTTTTGGTGTTGTGTTGTTCGAAGCTGTTTGTGCTCGAGCTGTTATAAACCCAACTTTGCCTAAAGACCAGATCAAC---------CTTGCGGAATGG : 2205
FER       : GAGTATTTCAGACGGCAG------CAACTGACTGAGAAATCCGATGTCTACTCCTTTGGCGTTGTTCTATTCGAAGCTCTATGCGCTCGTCCAGCCTTGAACCCAACACTTGCAAAGGAACAAGTGAGC---------TTAGCTGAGTGG : 2256
ANX1      : GAATACTTCAGAAGACAA------CAACTGACTGAGAAATCAGACGTTTACTCATTCGGAGTTGTTCTGTTCGAAATCTTATGCGCAAGACCAGCGTTAAACCCGAGTTTGCCTAAGGAACAAGTGAGT---------CTCGGAGACTGG : 2196
ANX2      : GAGTATTTCAGAAGACAA------CAGTTAACTGAGAAGTCAGATGTGTACTCATTCGGAGTTGTTCTATTCGAGGTTTTATGCGCTAGGCCAGCGTTAAACCCTAGTTTATCTAAAGAACAAGTTAGT---------CTTGGAGATTGG : 2208
CVY1      : GAGTATCTAACAAGACAA------CAATTAACTGAGAAATCTGATGTTTACTCTTTTGGTGTGGTGATGCTTGAGGTAGTATGTGCTAGACCAGTCATTGATCCATCACTACCGAGAGAGAAAGTGAAT---------TTGATCGAATGG : 2109
ERU-CAP1  : GAATACTTCCGCAGACAA------CAACTCACTGATAAATCCGATGTTTACTCATTCGGTGTGGTGTTATTCGAGGTTCTTTGCGCGAGGCCAGTGATAAACCCACAGTTACCTAGAGAACAAGTTAAC---------TTAGCAGAATAT : 2235
AT2G23200 : GAATATCTCCAAACACAT------AAATTAACAGAGAAATCAGATGTTTACGCATTCGGAGTCGTCCTCCTCGAGGTACTGTTCCCGAGACCAGCTATAGATCCTTATCTTCCTCACGAAGAAGTGAAT---------CTATCGGAATGG : 2109
AT5G24010 : GAATACTTCAGAAGACAA------CAGCTCACGGATAAGTCCGATGTTTATTCATTCGGTGTTGTTCTTTTTGAAGTTCTTTGCGCTAGACCCGCTGTTGATCCGTTGCTTGTTAGGGAGCAAGTAAAT---------TTAGCAGAATGG : 2112
AT5G59700 : GAATACTTCAGAAGACAA------CAGCTCACAGAGAAATCAGATGTTTACTCTTTTGGAGTGGTTATGTTCGAGGTTCTCTGCGCGAGACCTGTTATAGACCCGACACTTACTAGAGAAATGGTGAAT---------CTAGCGGAATGG : 2091
BUPS2     : GAATACTTCAGGAGACAG------CAACTTACAGACAAATCCGATGTTTATTCCTTTGGTGTTGTTCTACTAGAAGCTCTATGCGCAAGACCAGCCATTAATCCTCAGCTTCCAAGGAACAACTTAAC---------TTAGCCGAATGG : 2217
BUPS1     : GAGTACTTTAGAAGACAA------CAACTTACCGACAAATCCGATGTTTATTCCTTTGGTGTCGTTCTTCTAGAAGCTCTGTGCGCAAGACCAGCCATCAATCCTCAGCTTACCAAGAGAGCAGGTTAAC---------TTAGCTGAATGG : 2220

                         2560         *        2580         *        2600         *        2620         *        2640         *        2660         *        2680         *        2700
MDS1      : GTGAAGTCAAATTTCAATAAAGAACCGTTGATCAGATCATTGACTCAGATTTAACCGCTGATATCACTTCGACCTCGATGGAGAAGTTTTGTGAGATAGCCATTAGATGTGTTCAAGACCGTGGTATGGAACGGCCACCGATGAACGAC : 2385
MDS2      : GTGAAGTCAAATTACAGAAGAGGAACCGTTGATCAGATCATTGACTCAGATTTATCGGCTGATATCACTTCGACATCGTTGGAAAAGTTTTGTGAGATAGCCGTTAGATGCGTTCAGGACCGTGGTATGGAACGGCCACCGATGAACGAC : 2364
MDS3      : ATCTATAAAGATCTTGAAAATGGAGATCAAACGTGGATAATTGGAGATGAAATAAATGAAGAG---GACAACAAGATTGTGAAGAAGATGATATTGGTGAGTCTTTGGTGTATTCGACCATGCCCATCAGATCGTCCACCGATGAACAAA : 2286
MDS4      : ATTTATAAGGATCTTGAGGATGGAGAACAAACATGGATATTTGGTGATGAAATAACCAAAGAA---GAAAAAGAGATTGCTAAGAAGATGATTGTGGTAGGACTCTGGTGTATCCAACCATGCCCATCAGATCGTCCATCGATGAATAGA : 2298
THE1      : GCAATGGCGTGGCAGAAAAAGGGTCTGCTAGATCAAATCATGGACAGTAACTTAACCGGGAAGGTGAACCCTGCCTCGTTGAAGAAATTTGGAGAAACCGCAGAGAAATGTTTAGCGGAATACGGTGTGGACCGGCCTTCTATGGGAGAT : 2325
HERK1     : GCTATGAAATGGCAGAAGAAAGGGCAACTGGATCAGATCATCGACCAGTCGCTTCGCGGAAATATCAGACCCGATTCGTTAAGGAAATTTGCGGAAACAGGTGAGAAATGTTTAGCGGATTATGGAGTTGATAGGCCATCTATGGGAGAT : 2250
HERK2     : GCTTTAAGCTGGCAAAAACAGAGAAACCTCGAGTCCATCATCGACTCAAATCTGAGGGGAAACTACAGTCCTGAATCGTTGGAGAAGTATGGGGAGATAGCAGAGAAGTGCTTAGCGGATGAAGGGAAGAACAGGCCAATGATGGGAGAA : 2355
FER       : GCACCATACTGCTACAAGAAAGGCATGCTAGATCAAATCGTTGATCCCTACCTCAAGGGCAAGATCACACCAGAATGCTTCAAAAAGTTTGCTGAAACCGCGATGAAGTGTGTTCTAGACCAGGCATTGAGAGACCATCAATGGGAGAT : 2406
ANX1      : GCAATGAATTGCAAACGAAAAGGGAATTTAGAGGATATAATCGATCCGAATCTGAAAGGGAAGATCAATGCAGAGTGTTTGAAGAAATTTGCAGACACGGCGGAGAAGTGTCTTAACGATAGTGGATTAGAACGGCCGACAATGGGAGAT : 2346
ANX2      : GCTATGAACTGTAAACGTAAAGGGACATTAGAAGACATCATTGATCCTAATCTTAAAGGAAAGATTAATCCAGAGTGTTTGAAGAAATTTGCGGATACGGCGGAGAAGTGTTTATCTGATAGTGGCTTAGATCGACCAACGATGGGAGAT : 2358
CVY1      : GCGATGAAGTTGGTGAAGAAAGGGAAATTGGAAGATATCATAGACCCATTTCTTGTAGGAAAAGTAAAGCTTGAAGAGGTGAAAAAGTACTGTGAGGTAACAGAGAAGTGTTTGTCCCAAAACGGAATTGAGAGGCCAGCAATGGGAGAT : 2259
ERU-CAP1  : GCTATGAATTTGCATAGAAAAGGGATATGTTGGAGAAATCATCGACCCGAAAATCGTGGGAACGATTAGTAAAGGATCGTTAAGGAAATTCGTGGAGGCTGCAGAAAAATGTTTGGCAGAGTACGGTGTTGATCGACCGGGAATGGGAGAT : 2385
AT2G23200 : GCTGATGTTCTGCAAATCAAAAGGTACAATCGATGAGATCTTAGATCCGAGTTTGATAGGTCAAATTGAGACGAATTCGTTGAAGAAATTCATGGAAATTGCTGAGAAATGTTTGAAAGAGTATGGAGATGAGAGACCGAGTATGAGGGAT : 2259
AT5G24010 : GCCAATAGAGTGGCAAAGGAAAGGGATGCTTGATCAGATCGTTGATCCGAATATCGCAGATGAAATCAAACCGTGCTCGTTGAAGAAATTCGCTGAAACCGCAGAGAAATGCTGTGCGGATTACGGTGTAGATAGACCGACAATGGCGAT : 2262
AT5G59700 : GCAATGAAATGGCAGAAGAAAGGACAGCTTGAACACATCATCGACCCGTCACTGCGGGGTAAGATCAGACCTGACTCGCTTAGAAAGTTCGGTGAAACAGGGGAGAAATGTTTAGCTGACTATGGAGTCGATAGACCATCAATGGGAGAT : 2241
BUPS2     : GCTATGCTATGGAAACAAAAAGGTTTACTCGAAAGATCATCGACCCTCATCTCGTTGGTGCTGTGAATCCTGAATCAATGAAAAAGTTTGCAGAAGCTGCAGAGAAGTGTTTAGCAGATTATGGTGTTGATAGACCAACAATGGGAGAT : 2367
BUPS1     : GCGATGCAATGGAAACGAAAGGACTTCTTGAAAAGATCATTGATCCTCATTTGGCTGGAACGATTAATCCTGAATCGATGAAGAAGTTTGCTGAAGCTGCAGAGAAGTGTTTAGAAGACTACGGTGTCGATAGGCCAACAATGGGAGAT : 2370
```

```
                          *       2720        *       2740        *       2760        *       2780        *       2800        *       2820        *       2840        *
MDS1      : GTTGTTTGGGCGCTTGAGTTTGCTCTTCAGCTTCACGAGACTGCTAAGAAGAAGAATGACAACGTGGAGTCT------------------------CTGGATCTAATGCCAAGTGGTGAAGTTGGTACGACCACGGACGGAGAAGATGAC : 2511
MDS2      : GTTGTTTGGGCGCTTGAGTTTGCTCTTCAGCTTCACGAGACTGCTAAGAAGAAGAATGACAACGTGGAGTCT------------------------CTGGATCTAATGCCAAGTGGTGAGGTTGGTACGACCACGGACGGAGAAGATGAC : 2490
MDS3      : GTTGTC---------------------------------------GAGATGATAGAGGGCAGTTTGGATGCT------------------------CTTGAACTCCCCCCT--------------------------------------- : 2334
MDS4      : GTCGTT---------------------------------------GAAATGATGGAAGGAAGTTTAGATGCT------------------------CTTGAAATTCCTCCA--------------------------------------- : 2346
THE1      : GTATTGTGGAATTTGGAGTACGCGTTACAGCTAGAAGAAACATCTTCGGCTTTGATGGAGCCTGATGACAATAGTACAAACCACATT------------CCAGGGATTCCAATGGCGCCAATGGAACCGTTTGATAACAGT--------- : 2454
HERK1     : GTGTTGTGGAATCTTGAATACGCTCTGCAGCTTCAAGAAGCAGTCATTGAT---GGTGAACCAGAAGATAATAGCACGAATATGATT------------GGTGAATTACCTCCG---CAGATCAATAATTTCAGTCAGGGAGACACTAGT : 2382
HERK2     : GTGTTATGGAGCTTGGAGTATGTTTTGCAGATTCATGAAGCTTGGCTTCGCAAACAGAATGGAGAAAACTCGTTTTCG---------------------------------------------------------------------AGC : 2436
FER       : GTTCTGTGGAACTTAGAATTTGCGTTGCAGCTCCAGGAAAGCGCAGAAGAGAACGGAAAAGGAGTATGCGGTGACATGGACATGGATGAGATTAAGTACGATGATGGAAACTGTAAAGGAAAGAACGACAAGAGTTCTGATGTGTATGAA : 2556
ANX1      : GTTTTGTGGAATCTCGAATTCGCACTTCAGCTTCAAGAGACTGCTGACGGGACT------------------------------------------------------------CGTCATCGGACTCCGAATAATGGAGGAAGCTCTGAG : 2436
ANX2      : GTCTTGTGGAATCTTGAATTTGCACTTCAGTTACAAGAAACTGCTGATGGATCA------------------------------------------------------------CGTCACCGAACGCCGAGCAATGGTGGTGGTTCTGTG : 2448
CVY1      : TTGTTGTGGAACCTAGAGTTCATGCTTCAGGTGCAA------------------------GCAAAAGATGAGAAAGCAGCAATGGTG------------GATGATAAGCCAGAGGCGAGTGTC--------------------------- : 2346
ERU-CAP1  : GTGTTGTGGAATCTTGAATATGCTTTGCAGCTTCAAGAAGCATCGGCTCAA---GTTGATTTATCGGAGGATAAGACTACAATG------------------------------------------------------------------ : 2466
AT2G23200 : GTGATTTGGGATCTTGAGTATGTTCTTCAGCTACAGATGATGACGAATCGTCGAGAGGCTCATGAAGAAGATAGTACGGCGATTAAT------------------------------------TCCGGTGGTTCGTTGGTTGCTCCGAGG : 2373
AT5G24010 : GTTCTATGGAACTTAGAACATGTGCTTCAGCTTCAAGAATCC---GGACCATTGAACATTCCTGAAGAAGATTATGGAGATGTTACC------------GAC------CCGAGAACGGCTCGGCAAGGTCTGTCTAATGGCTCGAACATA : 2391
AT5G59700 : GTGTTGTGGAATCTTGAATACGCTTTGCAGCTACAAGAAGCTGTCGTTGAT---GGTGATCCAGAAGAC---AGCACAAACATGATC------------GGTGAGTTACCTCTA---CGGTTCAATGATTACAACCATGGAGACACGAGT : 2370
BUPS2     : GTATTGTGGAACTTAGAATATGCTCTTCAGCTTCAAGAGGCTTTTTCTCAG---GGTAAAGCTGAGGCTGAGGAG---------------------------GTTGAGACTCCCAAGCCTGTGGCTGTCCCTGCTGCTGCGCCGACTTCA : 2487
BUPS1     : GTTCTGTGGAACTTGGAGTATGCTCTTCAGCTTCAAGAGGCATTCACTCAG---GGCAAAGCAGAAGAGACCGAGAACGCTAAGCCT------------GATGTGGTGACACCTGGCTCGGTGCCTGTGTCTGATCCATCTCCGATCACT : 2505

                  2860        *       2880        *       2900        *       2920        *       2940        *       2960        *       2980
MDS1      : TTGTTTAGTAGGACTACAGGACACGTTGGGAAATCGACCACGACCGATGACTCTGTTCTAGTTGTTGGTGATGAGAGGAGTGGTTCGAGTTGGGGAGTATTTTCGGAGATCAATGAACCTAAAGCACGGTAG : 2643
MDS2      : TTGTTTAGTAGGACTACAGGACACGTGGGGAAATCGACCACGACCGATGACTCTGTTCTAGTTGTTGGTGATGAGAGGAGTGGTTCGAGTTGGGGAGTATTTTCGGAGATCAATGAACCTAAAGCACGGTAG : 2622
MDS3      : ---------AAGCCTTCTAGGCATATTTCCACCGAGTTAGTTCTTGAATCTTCTTCGCTTTCTGATGGCCAAGAAGCTGAAAAACAAACT---------------CAAACCTTAGATTCTACAATTATTTAG : 2442
MDS4      : ---------AAACCTTCTATGCATATCTCCACTGAGGTTATTACCGAATCTTCTTCACTCTCTGATGGCGGGGAAGAT------------------------------------------------GTATAA : 2421
THE1      : ------ATGAGTATAATCGATAGAGGAGGAGTA------------AATTCGGGGACCGGGACTGATGATGATGCGGAAGACGCGACTACTAGTGCGGTGTTTTCGCAGCTTGTTCATCCTCGTGGAAGGTAG : 2568
HERK1     : GTTAACGTTCCG---GGCACAGCGGGGCGATTC------------------GAGGAATCTAGTATTGATGATCTCTCTGGCGTTTCCATGAGTAAAGTATTCTCACAACTGGTGAAATCTGAAGGAAGATAG : 2493
HERK2     : AGCCAAGCGGTAGAAGAAGCACCAGAGAGCTTTACTCTTCCAGCTTGTTCCAATCAAGATTCCTCAGAAACTGAGCAGAGCCAAACAGGATCTGCTCTT------------------CACAATTCGGCTTAG : 2550
FER       : GGGAATGTGACGGACTCGAGGAGCAGTGGAATAGATATGAGCATCGGTGGTAGGAGTTTGGCCAGCGAAGATTCAGATGGACTCACTCCAAGTGCTGTGTTTTCTCAGATCATGAATCCAAAGGACGTTAG : 2688
ANX1      : GATCTCGGTAGAGGAGGCATGGCGGTTAACGTC---------GCCGGTAGAGATGATGTG---AGTGAT---CTCTCCTCAGAGGACAATACCGAAATATTCTCTCAGATTGTAAATCCCAAAGGACGATAA : 2553
ANX2      : GATTTAGGTGGAGGAGGAGGAGGTGTGACGGTGAACATCAGCGCCGGAGAAAGTGACTTG---GGTGATGACTTGTCATCTGAAGAAAACAGTGGAATATTTTCTCAGATTGTAAACCCTAAAGGACGATAA : 2577
CVY1      : ------------GTTGGCTCGACCATGCAGTTTAGTGTG------------------AATGGAGTGGAGATATTGCAGGGGTCTCAATGAGCAAAGTTTCGCACAAATGGTTAGAGAAGAAACGAGATAA : 2448
ERU-CAP1  : ---------------------------------------------------------------AATATTGAAATGGACCTTATTCCCGGCGAGGAAATG------CAATCTCCGTCGCATTCAATACCGTGA : 2529
AT2G23200 : TTGATGGTGAGTGATTCGTTTAGTACGAACTCGATATTTCAGAATGGTGATGAATCGAAGAACAGATTTGGATTTACAGATTCATCGGAGACTCGAGTTTTTTCGCAGTTGAAGATCTCTGATGCAAGATGA : 2505
AT5G24010 : ------------------------------------------------GAGAGAGACTATGGAGATGGAACTTCAGGCATAATCAGTAGCACCCAAGTGTTCTCTCAGCTAATGACCAACGCTGGGAGATAA : 2475
AT5G59700 : GTTAACTTTTCTGTAGCTAAAGAGGGACGGTTT------------GATGAAGAAGAATCGAGTGTTGATGATAGTTCAGGTGTTTCCATGAGTAAAGTTTTCTCGCAGCTGATCAAATCTGAGGGACGTTAA : 2490
BUPS2     : CCTGCTGCCACCACTGCTGCAGCAAGTGAACGTCCAGTTTCT---CAGACTGAAGAGAAGGATGACTCAACGGTGGACCAACACTCTGGAACCACCATGTTTACTCAGTTTGCTAGCCTTAACGGAAGATAA : 2616
BUPS1     : CCCTCAGTCACCACCAACGAAGCGGCTACTGTTCCGGTTCCTGCTAAGGTGGAAGAGAACAGTGGCACGGCCGTGGATGAACACTCTGGAACAGCCATGTTTACTCAGTTTGCTAACCTTAACGGAAGATAA : 2637
```

(b)

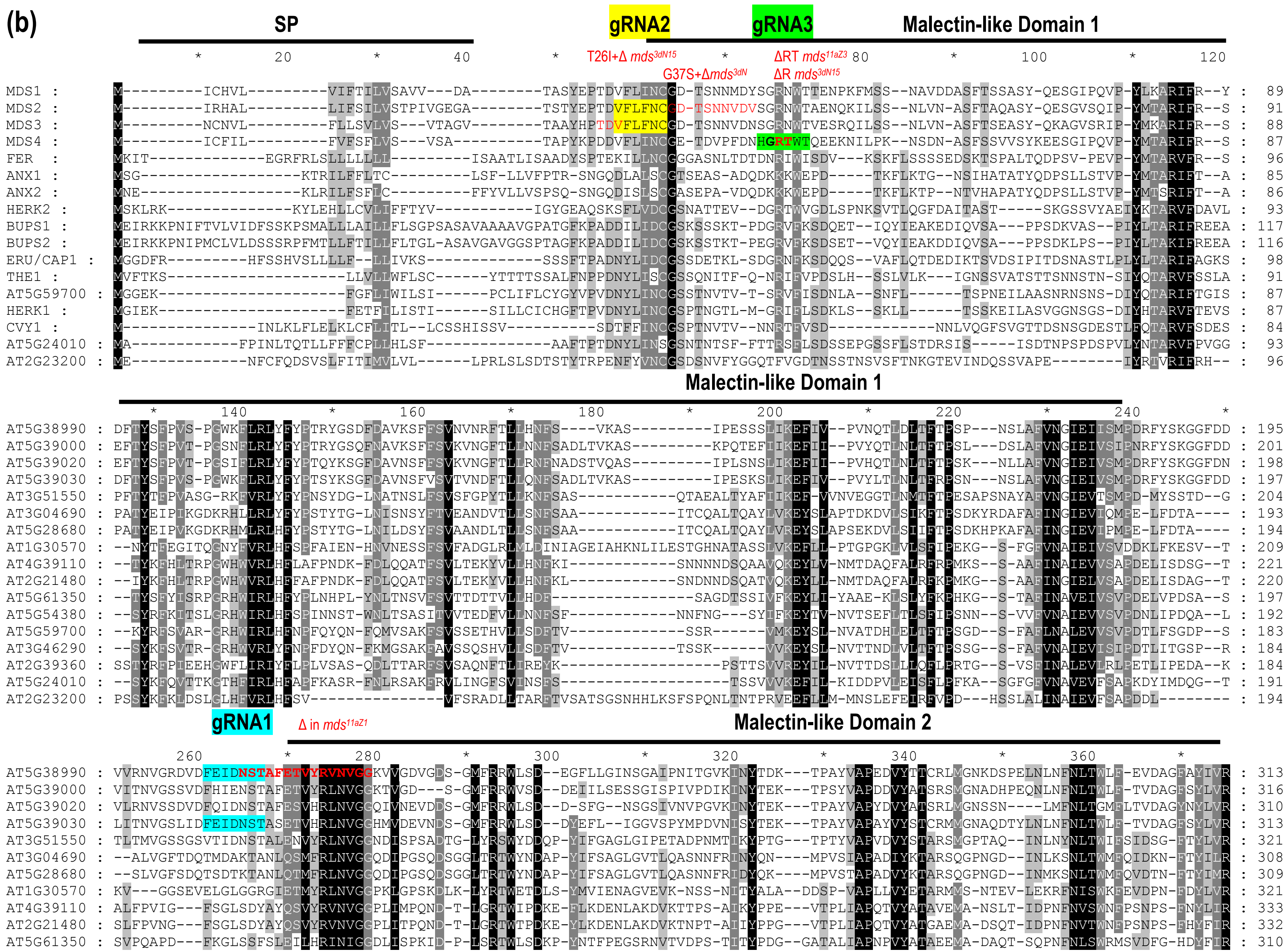

SP
gRNA2
gRNA3
Malectin-like Domain 1
T26I+Δ mds3dN15
G37S+Δmds3dN
ΔRT mds11aZ3
ΔR mds3dN15
MDS1 : M---------ICHVL---------VIFTILVSAVV---DA--------TASYEPTDVFLINCGD-TSNNMDYSGRNWTTENPKFMSS--NAVDDASFTSSASY-QESGIPQVP-YLKARIFR--Y : 89
MDS2 : M---------IRHAL---------LIFSILVSTPIVGEGA--------TSTYEPTDVFLFNCGD-TSNNVDVSGRNWTAENQKILSS--NLVN-ASFTAQASY-QESGVSQIP-YMTARIFR--S : 91
MDS3 : M---------NCNVL---------FLLSVLVS---VTAGV--------TAAYHPTDVFLFNCGD-TSNNVDNSGRNWTVESRQILSS--NLVN-ASFTSEASY-QKAGVSRIP-YMKARIFR--S : 88
MDS4 : M---------ICFIL---------FVFSFLVS---VSA----------TAPYKPDDVFLINCGE-TDVPFDNHGRTWTQEEKNILPK--NSDN-ASFSSVVSYKEESGIPQVP-YMTARIFR--S : 87
FER : MKIT-------------EGRFRLSLLLLLL----------ISAATLISAADYSPTEKILLNCGGGASNLTDTDNRIWISDV---KSKFLSSSSEDSKTSPALTQDPSV-PEVP-YMTARVFR--S : 96
ANX1 : MSG------------------KTRILFFLTC---------LSF-LLVFPTR-SNGQDLALSCGTSEAS-ADQDKKKWEPD-----TKFLKTG--NSIHATATYQDPSLLSTVP-YMTARIFT--A : 85
ANX2 : MNE------------------KLRILFSFLC---------FFYVLLVSPSQ-SNGQDISLSCGASEPA-VDQDKKKWEPD-----TKFLKTP--NTVHAPATYQDPSLLSTVP-YMTSRIFT--A : 86
HERK2 : MSKLRK---------------KYLEHLLCVLIFFTYV------------IGYGEAQSKSFLVDCGSNATTEV--DGRTWVGDLSPNKSVTLQGFDAITAST-----SKGSSVYAEIYKTARVFDAVL : 93
BUPS1 : MEIRKKPNIFTVLVIDFSSKPSMALLLAILLFLSGPSASAVAAAAVGPATGFKPADDILIDCGSKSSSKT-PDGRVFKSDQET--IQYIEAKEDIQVSA---PPSDKVAS--PIYLTARIFREEA : 117
BUPS2 : MEIRKKPNIPMCLVLDSSSRPFMTLLFTILLFLTGL-ASAVGAVGGSPTAGFKPADDILIDCGSKSSTKT-PEGRVFKSDSET--VQYIEAKDDIQVSA---PPSDKLPS--PIYLTAKIFREEA : 116
ERU/CAP1 : MGGDFR---------HFSSHVSLLLLF--LLIVKS-------------SSSFTPADNYLIDCGSSDETKL-SDGRNFKSDQQS--VAFLQTDEDIKTSVDSIPITDSNASTLPLYLTARIFAGKS : 98
THE1 : MVFTKS---------------------LLVLLWFLSC------YTTTTSSALFNPPDNYLISCGSSQNITF-Q-NRIFVPDSLH--SSLVLK---IGNSSVATSTTSNNSTN-SIYQTARVFSSLA : 91
AT5G59700 : MGGEK---------------------FGFLIWILSI------PCLIFLCYGYVPVDNYLINCGSSTNVTV-T-SRVFISDNLA--SNFL------TSPNEILAASNRNSNS-DIYQTARIFTGIS : 87
HERK1 : MGIEK---------------------FETFLISTI------SILLCICHGFTPVDNYLINCGSPTNGTL-M-GRIFLSDKLS--SKLL------TSSKEILASVGGNSGS-DIYHTARVFTEVS : 87
CVY1 : M--------------INLKLFLELKLCFLITL--LCSSHISSV----------SDTFFINCGSPTNVTV--NNRTFVSD-----------------NNLVQGFSVGTTDSNSGDESTLFQTARVFSDES : 84
AT5G24010 : MA------------FPINLTQTLLFFFCPLLHLSF-------------AAFTPTDNYLINSGSNTNTSF-FTTRSFLSDSSEPGSSFLSTDRSIS-----ISDTNPSPDSPVLYNTARVFPVGG : 93
AT2G23200 : ME-------------NFCFQDSVSLFITIMVLVL------LPRLSLSDTSTYTRPENFYVNCGSDSNVFYGGQTFVGDTNSSTNSVSFTNKGTEVINDQSSVAPE--------IYRTVRIFRH-- : 96
Malectin-like Domain 1
AT5G38990 : DFTYSFPVS-PGWKFLRLYFYPTRYGSDFDAVKSFFSVNVNRFTLLHNFS----VKAS---------IPESSSLIKEFIV--PVNQTLDLTFTPSP---NSLAFVNGIEIISMPDRFYSKGGFDD : 195
AT5G39000 : EFTYSFPVT-PGSNFLRLYFYPTRYGSQFNAVKSFFSVKVNGFTLLNNFSADLTVKAS---------KPQTEFIIKEFII--PVYQTLNLTFTPSL---DSLAFVNGIEIVSIPNRFYSKGGFDD : 201
AT5G39020 : EFTYSFPVT-PGSIFLRLYFYPTQYKSGFDAVNSFFSVKVNGFTLLNNFNADSTVQAS---------IPLSNSLIKEFII--PVHQTLNLTFTPSK---NLLAFVNGIEIVSMPDRFYSKGGFDN : 198
AT5G39030 : DFTYSFPVS-PGWKFLRLYFYPTSYKSGFDAVNSFVSVTVNDFTLLQNFSADLTVKAS---------IPESKSLIKEFIV--PVYLTLNLTFRPSN---NSLAFVNGIEIVSMPDRFYSKGGFDD : 197
AT3G51550 : PFTYTFPVASG-RKFVRLYFYPNSYDG-LNATNSLFSVSFGPYTLLKNFSAS-----------QTAEALTYAFIIKEF-VVNVEGGTLNMTFTPESAPSNAYAFVNGIEVTSMPD-MYSSTD--G : 204
AT3G04690 : PATYEIPIKGDKRHLLRLYFYPSTYTG-LNISNSYFTVEANDVTLLSNFSAA-----------ITCQALTQAYLVKEYSLAPTDKDVLSIKFTPSDKYRDAFAFINGIEVIQMPE-LFDTA---- : 193
AT5G28680 : PATYEIPVKGDKRHMLRLHFYPSTYTG-LNILDSYFSVAANDLTLLSNFSAA-----------ITCQALTQAYLVREYSLAPSEKDVLSIIFTPSDKHPKAFAFINGIEVIPMPE-LFDTA---- : 194
AT1G30570 : --NYTFEGITQGNYFVRLHFSPFAIEN-HNVNESSFSVFADGLRLMLDINIAGEIAHKNLILESTGHNATASSLVKEFLL-PTGPGKLVLSFIPEKG--S-FGFVNAIEIVSVDDKLFKESV--T : 209
AT4G39110 : --TYKFHLTRPGWHWVRLHFLAFPNDK-FDLQQATFSVLTEKYVLLHNFKI------------SNNNNDSQAAVQKEYLV-NMTDAQFALRFRPMKS--S-AAFINAIEVVSAPDELISDSG--T : 221
AT2G21480 : --IYKFHLTRPGWHWVRLHFFAFPNDK-FDLQQATFSVLTEKYVLLHNFKL------------SNDNNDSQATVQKEYLL-NMTDAQFALRFKPMKG--S-AAFINGIELVSAPDELISDAG--T : 220
AT5G61350 : --TYSFYISRPGRHWIRLHFYPLNHPL-YNLTNSVFSVTTDTTVLLHDF--------------SAGDTSSIVFKEYLI-YAAE-KLSLYFKPHKG--S-TAFINAVEIVSVPDELVPDSA--S : 197
AT5G54380 : --SYRFKITSLGRHWIRLHFSPINNST-WNLTSASITVVTEDFVLLNNFSF------------NNFNG--SYIFKEYTV-NVTSEFLTLSFIPSNN--S-VVFVNAIEVVSVPDNLIPDQA--L : 192
AT5G59700 : --KYRFSVAR-GRHWIRLHFNPFQYQN-FQMVSAKFSVSSETHVLLSDFTV-------------SSR------VMKEYSL-NVATDHLELTFTPSGD--S-FAFLNALEVVSVPDTLFSGDP--S : 183
AT3G46290 : --SYKFSVTR-GRHWVRLYFNPFDYQN-FKMGSAKFAVSSQSHVLLSDFTV-------------TSSK------VVKEYSL-NVTTNDLVLTFTPSSG--S-FAFVNAIEVISIPDTLITGSP--R : 184
AT2G39360 : SSTYRFPIEEHGWFLIRIYFLPLVSAS-QDLTTARFSVSAQNFTLIREYK-----------------PSTTSVVREYIL-NVTTDSLLLQFLPRTG--S-VSFINALEVLRLPETLIPEDA--K : 184
AT5G24010 : --SYKFQVTTKGTHFIRLHFAPFKASR-FNLRSAKFRVLINGFSVINSFS-----------------TSSVVVKEFIL-KIDDPVLEISFLPFKA--SGFGFVNAVEVFSAPKDYIMDQG--T : 191
AT2G23200 : PSSYKFKLDSLGLHFVRLHFSV--------------VFSRADLLTARFTVSATSGSNHHLKSFSPQNLTNTPRVEEFLLM-MNSLEFEIRFVPD---HSSLALINAIEVFSAPDDL-------- : 194
gRNA1
Δ in mds11aZ1
Malectin-like Domain 2
AT5G38990 : VVRNVGRDVDFEIDNSTAFETVYRVNVGGKVVGDVGDS-GMFRRWLSD--EGFLLGINSGAIPNITGVKINYTDK---TPAYVAPEDVYTTCRLMGNKDSPELNLNFNLTWLF-EVDAGFAYIVR : 313
AT5G39000 : VITNVGSSVDFHIENSTAFETVYRLNVGGKTVGD---S-GMFRRWVSD--DEIILSESSGISPIVPDIKINYTEK---TPSYVAPDDVYATSRSMGNADHPEQNLNFNLTWLF-TVDAGFSYLVR : 316
AT5G39020 : VLRNVSSDVDFQIDNSTAFESVHRLNVGGQIVNEVDDS-GMFRRWLSD--D-SFG--NSGSIVNVPGVKINYTEK---TPAYVAPYDVYATSRLMGNSSN----LMFNLTGMFLTVDAGYNYLVR : 310
AT5G39030 : LITNVGSLIDFEIDNSTASETVHRLNVGGHMVDEVNDS-GMFRRWLSD--DYEFL--IGGVSPYMPDVNISYTEK---TPAYVAPAYVYSTCRMMGNAQDTYLNLNFNLTWLF-TVDAGFSYLVR : 313
AT3G51550 : TLTMVGSSGSVTIDNSTALENVYRLNVGGNDISPSADTG-LYRSWYDDQP-YIFGAGLGIPETADPNMTIKYPTG---TPTYVAPVDVYSTARSMGPTAQ--INLNYNLTWIFSIDSG-FTYLVR : 321
AT3G04690 : --ALVGFTDQTMDAKTANLQSMFRLNVGGQDIPGSQDSGGLTRTWYNDAP-YIFSAGLGVTLQASNNFRINYQN----MPVSIAPADIYKTARSQGPNGD--INLKSNLTWMFQIDKN-FTYILR : 308
AT5G28680 : --SLVGFSDQTSDTKTANLQTMFRLNVGGQDIPGSQDSGGLTRTWYNDAP-YIFSAGLGVTLQASNNFRIDYQK----MPVSTAPADVYKTARSQGPNGD--INMKSNLTWMFQVDTN-FTYIMR : 309
AT1G30570 : KV---GGSEVELGLGGRGIETMYRLNVGGPKLGPSKDLK-LYRTWETDLS-YMVIENAGVEVK-NSS-NITYALA--DDSP-VAPLLVYETARMMS-NTEV-LEKRFNISWKFEVDPN-FDYLVR : 321
AT4G39110 : ALFPVIG---FSGLSDYAYQSVYRVNVGGPLIMPQND-T-LGRTWIPDKE-FLKDENLAKDVKTTPS-AIKYPPE---VTPLIAPQTVYATAVEMA-NSLT-IDPNFNVSWNFPSNPS-FNYLIR : 333
AT2G21480 : SLFPVNG---FSGLSDYAYQSVYRVNVGGPLITPQND-T-LGRTWTPDKE-YLKDENLAKDVKTNPT-AIIYPPG---VTPLIAPQTVYATGAEMA-DSQT-IDPNFNVTWNFPSNPS-FHYFIR : 332
AT5G61350 : SVPQAPD---FKGLSSFSLEILHRINIGGDLISPKID-P-LSRTWLSDKP-YNTFPEGSRNVTVDPS-TITYPDG--GATALIAPNPVYATAEEMA-DAQT-SQPNFNLSWRMSVDFG-HDYFIR : 310

```
AT5G54380 : ALNPSTP---FSGLSLLAFETVYRLNMGGPLLTSQND-T-IGRQWDNDAE-YLHVNSSVLVVTANPS-SIKYSPS---VTQETAPNMVYATADTMG-DANV-ASPSFNVTWVLPVDPD-FRYFVR : 304
AT5G59700 : FAGSPGK---FQGLSWQALETVYRVNMGGPRVTPSND-T-LSRIWEPDSE-FL-VEKNLVKSVSKIA-SVDYVPG--FATEETAPRTVYGTCTEMN-SADN-PSSNFNVTWDFDVDPG-FQYFLR : 295
AT3G46290 : FVGNPAQ---FPDMSMQGLETIHRVNMGGPLVASNND-T-LTRTWVPDSE-FL-LEKNLAKSMSKFS-TVNFVPG--YATEDSAPRTVYGSCTEMN-SADN-PNSIFNVTWEFDVDPG-FQYYFR : 296
AT2G39360 : LIGTQKDLK----LSSHAMETVSRVNMGNLSVSRDQD-K-LWRQWDSDSA-Y---KAHFGTPVMNLK-AVNFSAG--GITDDIAPVYVYGTATRLNSD-LD-PNTNANLTWTFKVEPG-FDYFVR : 293
AT5G24010 : KLVIPNSAQIFSNLSSQVLETVHRINVGGSKLTPFND-T-LWRTWVVDDN-YLLLRAAARRAWTTHS-P-NYQNG--GATREIAPDNVYMTAQEMDRDNQE-LQARFNISWGFQVDEKRVLHLVR : 308
AT2G23200 : ---------EIPSASDKNLHTIYRLNVGGEKITPDNDT--LGRTWLPDDDDFLYRKDSARNINSTQ--TPNYVGGLSSATDSTAPDFVYKTAKAMNRSSNEQVGMLMNVTWSFKVKSNH-RHFIR : 305
```

Malectin-like Domain 2

```
              380        *       400        *       420        *       440        *       460        *       480        *       500
AT5G38990 : LHFCETQPEVNKTGDRV-FSIFFGYQLAMREMDVFRL---SGGFRLPMYLDFKVLVDADGTSQRPSLRVDLTPYKEDYPTYYDAILSGVEILKLSNSDGNLA-----GLNPIP--QLSPP--PQS : 425
AT5G39000 : LHFCETLSEVNKEGQRV-FSIFIENQTATLEMDVFRM---SGGSWIPMYLDYTV-IAGSGSGRRHDLRLDLHPLVSINPKYYDAILNGVEILKMNDPDGNLA-----GPNPDP--LVSPDLIPNR : 429
AT5G39020 : LHFCETLPQVTKAGQRV-FSIFVEDKMAKKETDVIRL---SGGPRIPMYLDFSVYVGFESGMIQPELRLDLVPLKDTNQTYYDAILSGVEILKLNDSDGNLA-------RPNPELLVSTDSTPDD : 424
AT5G39030 : LHFFEKY--LNKANQRV-FSIFLGNQMAREEMDVIRL---SGGPRIPIYLDFRIYVGSESGP-RPDLRLDLHPLVKDNPEYYEAILNGVEILKLNNS-GNLAIIQDNELKPNPPL--SSNLTPN- : 427
AT3G51550 : LHFCEVSSNITKINQRV-FTIYLNNQTAEPE---ADVIAWTSSNGVPFHKDYV--VNPPEGNGQQDLWLALHPNPVNKPEYYDSLLNGVEIFKMNTSDGNLA-----GTNPIPGPQVTADPSKVL : 435
AT3G04690 : LHFCEF--QLSKINQKV-FNIYINNRTAQADTTPADIIGWTGEKGIPMYKDYAIYVDANNGG--EEITLQMTPSTFGQPEYYDSSLNGLEIFKMDTMK-NLA-----GPNPEPSPM---QAEEEV : 419
AT5G28680 : LHFCEF--QLAKINQKV-FNIFINNRTAQGDTNPADILGWTGGKGIPTYKDYAIYVDANTGGGGEEISLQMTPSTFGQPEYYDSQLNGLEIFKIDTMK-NLA-----GPNPKPSPM---QANEDV : 422
AT1G30570 : LHFCELLVDKQN---QRIFRIYINNQTAAGNFDIFAHAG--GKNKGIY-QDY---LDPVSS-KNDVLWIQLGPDSSVGAS-GDALLSGLEIFKLSKN-GNLA-----HLIRFDSTGHSVSDSK-- : 427
AT4G39110 : LHFCDIVSKSLN---DLYFNVYINGKTAISGLDLST-VA--GNLAAPYYKDI---VVNA-TLMGPELQVQIGPMGE-DTGTKNAILNGVEVLKMSNSVNSLD-----GEFGVDGR----TTGM-- : 436
AT2G21480 : LHFCDIISKSLN---DLYFNVYINGKTAISGLDLST-VA--GDLSAPYYKDI---VVNS-TLMTSELQVQIGPMGE-DTGKKNAILNGVEVLKMSNSVNSLD-----GEFGVDGQ----RASM-- : 435
AT5G61350 : LHFCDIVSKSLN---DLIFNVFINKLSAISALDLSS-LT--SALGTAYYADF---VLNASTITNGSILVQVGPTPNLQSGKPNAILNGLEIMKLNNAAGSLD-----GLFGVDGKYKGPIGGM-- : 419
AT5G54380 : VHFCDIVSQALN---TLVFNLYVNDDLALGSLDLST-LT--NGLKVPYFKDF---ISNGSVESSGVLTVSVGP-DS-QADITNATMNGLEVLKISNEAKSLS-----GVSSVKSLLPGGSGSK-- : 411
AT5G59700 : FHFCDIVSKALN---QLYFNLYVDSMDVVENLDLSSYLS--NTLSGAYAMDF---VTGSAKLTKRI-RVSIGP-SSVHTDYPTAILNGLEIMKMNNSKSQLS---------IGTFLPSGSSST-- : 399
AT3G46290 : FHFCDIVSLSLN---QLYFNLYVDSMVAATDIDLSTLVD--NTLAGAYSMDF---VTQTPKGSNKV-RVSIGP-STVHTDYPNAIVNGLEIMKMNNSKGQLS---------TGTFVP-GSSSS-- : 399
AT2G39360 : FHFCNIIVDPFGFERQIRFDIFVNSEK-VRTIDMTEVLN--GTFGAPFFVDA---VMRKAKSREGFLNLSIGLVMDV-SSYPVSFINGFEISKLSNDKRSLD-----AFDAI--LPDGSSSNK-- : 402
AT5G24010 : LHFCDIVSSSLN---QLYFNVFINEYLAFKDVDLSTLTF--HVLASPLYIDF---VAESDR--SGMLRISVGPSDLSNPARVNALLNGVEIMRI--------------LSPVSSEVVSGKRNV-- : 407
AT2G23200 : IHFSDILSNLSNSDSD--FYLFVNGYWRV-DVKPSE----QPRLASPFFKDVV-----NVSDGSGLLNISIGTK---EANKDAGFLNGLEMMEV--------------------LSKSGSDYSN : 394
```

TM

```
                     *       520        *       540        *       560        *       580        *       600        *       620
AT5G38990 : IT-PLKGKGKSSHVLPIIIAVVGSAVALAFFVLVVVLVV--MKRKKKSNESSVDTTNKPSTNSSWGPLLHGTGSTNTKSASSLPS---------------DLCRRFSIYEIKSATNDFEEKLIIG : 532
AT5G39000 : AT-P-RIRKNKSHILPITLAVVGSLVVLAMFV-VGVLVI--MKKKKKS---------KPSTNSSWCPLPHGTDSTNTKPAKSLPA---------------DLCRRFSIFEIKSATNDFEDKLIIG : 525
AT5G39020 : SNVTPPIKGKP-HVLVIILIVVGSVIGLATFIVIIMLLIRQMKRKKNKKENSVIMFKL------------------------------------------LLKQYIYAELKKITKSFS--HTVG : 503
AT5G39030 : -HVTQQIKGKSSHLLVKIFIAVGPGTGLATFVVVLMLWMRQMKRK-NRKEERVVMFKK------------------------------------------LLNMYTYAELKKITKSFS--YIIG : 505
AT3G51550 : RPTTRKSKSNTAIIAGAASGAVVLALIIGFCVF--GAYRRRKRGDYQPASDATSGWLPLSLYGNSHS---------AGSAKT----NTTGSYASSL--PSNLCRHFSFAEIKAATKNFDESRVLG : 543
AT3G04690 : KKEFKNEKR-HAFIIGSAGGVLAV-LIGALC-F--TAYKKKQ--GYQGGDSHTSSWLPI--YGNSTT---------SGTKSTISGKSNNGSHLSNL--AAGLCRRFSLPEIKHGTQNFDDSNVIG : 524
AT5G28680 : KKDFQGDKRITAFVIGSAGGVAAV-LFCALC-F--TMYQRKR--KFSGSDSHTSSWLPI--YGNSHT---------SATKSTISGKSNNGSHLSNL--AAGLCRRFSLSEIKHGTHNFDESNVIG : 528
AT1G30570 : -----MRIIWISVGAGIAIIFFVFLGILVVCLC-----KKRRSKSDESKNNPPGWRPLFLHVNNST-ANAKATGGSLRLNTLAAST---------------GRKFTLAEIRAATKNFDDGLAIG : 527
AT4G39110 : -----GKHG-M-VATAGFVMMFGAFIGLGA-----MVYKWKKRPQDWQKRNSFSSWL-LPIHAGDSTFMTSKGGSQKSNF---------------YNSTLGLGRYFSLSELQEATKNFEASQIIG : 533
AT2G21480 : -----GKQG-M-VATAGFVMMFGAFVGLGA-----MVYKWKKRPQDWQKRNSFSSWL-LPIHAGDSTFMTSKTGSHKSNL---------------YNSALGLGRYFSLSELQEVTKNFDASEIIG : 532
AT5G61350 : -----SSKK-LAIAGIGFVMALTAFLGVVV-----LLVRWQRRPKDWQKQNSFSSWL-LPLHASHSSYISSKGGSTSRRMSIFGSKKSKSNGFSSFFSNQGLGRYFPFTELQTATQNFDENAVCG : 532
AT5G54380 : -----SKKK--AVIIGSLVGAVTLILLIAVCCYCCLVASRKQRSTSPQEGGNGHPWLPLPLYGLSQTLTKSTASHKSATASCISLASTH------------LGRCFMFQEIMDATNKFDESSLLG : 517
AT5G59700 : -----TKKN-VGMIIGLTIGSL-LALVVLGGFF--VLYKKRGRD----QDGNSKTWIPLSSNG-------TTSS---SNGTTLASIASN------------SSYRIPLVAVKEATNSFDENRAIG : 489
AT3G46290 : -----SKSN-LGLIVGSAIGSL-LAVVFLGSCF--VLYKKRKRG----QDGHSKTWMPFSING-------TSMGSKYSNGTTLTSITTN------------ANYRIPFAAVKDATNNFDESRNIG : 492
AT2G39360 : -----SSNTSVGLIAGLSAALCVALVFGVVVSWWCI---RKRRRRNRQMQT-------VHSRGDDHQIKKNETGESL-------------------IFSSSKIGYRYPLALIKEATDDFDESLVIG : 494
AT5G24010 : -----VWIV-VGSVLGGFVFLSLFFLSVL--CLC-------RKNNKTRSSESTGWTPL------RRFRGSSNSRT-------TERTVSSSGYHTL---------RISFAELQSGTNNFDRSLVIG : 496
AT2G23200 : RSSSRVHIITGCAVAAAAASALVFSLLF-------MVFLKRRRSKKTKPEVEGTVWSPLPLHRGGS------------------SDNRPISQYHNSPLRNLHLGLTIPFTDILSATNNFDEQLLIG : 495
```

Serine/Threonine Kinase Domain

```
                  *       640        *       660        *       680        *       700        *       720        *       740        *
AT5G38990 : VGGFGSVYKGRIDGGATLVAVKRLEITSNQGAKEFDTELEMLSKLRHVHLVSLIGYCDDDNEMVLVYEYMPHGTLKDHLF---RRDKASDPPLSWKRRLEICIGAARGLQYLHT-GAKYTIIHRD : 653
AT5G39000 : VGGFGSVYKGQIDGGATLVAVKRLEITSNQGAKEFETELEMLSKLRHVHLVSLIGYCDEDNEMVLVYEYMPHGTLKDHLF---RRDKTSDPPLSWKRRLEICIGAARGLQYLHT-GAKYTIIHRD : 646
AT5G39020 : KGGFGTVYRGNLSNGRT-VAVKVLK-DLKGNGDDFINEVTSMSQTSHVNIVSLLGFCYEGSKRAIISEFLEHGSL-DQFI---SRNKSLTPNVT--TLYGIALGIARGLEYLHY-GCKTRIVHFD : 619
AT5G39030 : KGGFGTVYGGNLSNGRK-VAVKVLK-DLKGSAEDFINEVASMSQTSHVNIVSLLGFCFEGSKRAIVYEFLENGSL-DQFM---SRNKSLTQDVT--TLYGIALGIARGLEYLHY-GCKTRIVHFD : 621
AT3G51550 : VGGFGKVYRGEIDGGTTKVAIKRGNPMSEQGVHEFQTEIEMLSKLRHRHLVSLIGYCEENCEMILVYDYMAHGTMREHLYKTQN------PSLPWKQRLEICIGAARGLHYLHT-GAKHTIIHRD : 661
AT3G04690 : VGGFGKVYKGVIDGTT-KVAVKKSNPNSEQGLNEFETEIELLSRLRHKHLVSLIGYCDEGGEMCLVYDYMAFGTLREHLYNTKK------PQLTWKRRLEIAIGAARGLHYLHT-GAKYTIIHRD : 641
AT5G28680 : VGGFGKVYKGVIDGGT-KVAIKKSNPNSEQGLNEFETEIELLSRLRHKHLVSLIGYCDEGGEMCLIYDYMSLGTLREHLYNTKR------PQLTWKRRLEIAIGAARGLHYLHT-GAKYTIIHRD : 645
```

```
AT1G30570 : VGGFGKVYRGELEDGT-LIAIKRATPHSQQGLAEFETEIVMLSRLRHRHLVSLIGFCDEHNEMILVYEYMANGTLRSHLFGSN------LPPLSWKQRLEACIGSARGLHYLHT-GSERGIIHRD : 644
AT4G39110 : VGGFGNVYIGTLDDGT-KVAVKRGNPQSEQGITEFQTEIQMLSKLRHRHLVSLIGYCDENSEMILVYEFMSNGPFRDHLYGKN------LAPLTWKQRLEICIGSARGLHYLHT-GTAQGIIHRD : 650
AT2G21480 : VGGFGNVYIGTIDDGT-QVAIKRGNPQSEQGITEFHTEIQMLSKLRHRHLVSLIGYCDENAEMILVYEYMSNGPFRDHLYGKN------LSPLTWKQRLEICIGAARGLHYLHT-GTAQGIIHRD : 649
AT5G61350 : VGGFGKVYIGEIDGGT-QVAIKRGSQSSEQGINEFQTEIQMLSKLRHRHLVSLIGFCDENKEMILVYEYMSNGPLRDHLYGSKENDPNPIPTLSWKQRLEICIGSARGLHYLHT-GAAQGIIHRD : 655
AT5G54380 : VGGFGRVYKGTLEDGT-KVAVKRGNPRSEQGMAEFRTEIEMLSKLRHRHLVSLIGYCDERSEMILVYEYMANGPLRSHLYGAD------LPPLSWKQRLEICIGAARGLHYLHT-GASQSIIHRD : 634
AT5G59700 : VGGFGKVYKGELHDGT-KVAVKRANPKSQQGLAEFRTEIEMLSQFRHRHLVSLIGYCDENNEMILVYEYMENGTLKSHLYGSG------LLSLSWKQRLEICIGSARGLHYLHT-GDAKPVIHRD : 606
AT3G46290 : VGGFGKVYKGELNDGT-KVAVKRGNPKSQQGLAEFRTEIEMLSQFRHRHLVSLIGYCDENNEMILIYEYMENGTVKSHLYGSG------LPSLTWKQRLEICIGAARGLHYLHT-GDSKPVIHRD : 609
AT2G39360 : VGGFGKVYKGVLRDKT-EVAVKRGAPQSRQGLAEFKTEVEMLTQFRHRHLVSLIGYCDENSEMIIVYEYMEKGTLKDHLYDLDDK-----PRLSWRQRLEICVGAARGLHYLHT-GSTRAIIHRD : 612
AT5G24010 : VGGFGMVFRGSLKDNT-KVAVKRGSPGSRQGLPEFLSEITILSKIRHRHLVSLVGYCEEQSEMILVYEYMDKGPLKSHLYGSTN------PPLSWKQRLEVCIGAARGLHYLHT-GSSQGIIHRD : 613
AT2G23200 : KGGFGYVYKAILPDGT-KAAIKRGKTGSGQGILEFQTEIQVLSRIRHRHLVSLTGYCEENSEMILVYEFMEKGTLKEHLYGSN------LPSLTWKQRLEICIGAARGLDYLHSSGSEGAIIHRD : 613
```

Serine/Threonine Kinase Domain

```
                   760         *       780         *       800         *       820         *       840         *       860         *
AT5G38990 : IKTTNILLDENFVAKVSDFGLSRVGPTSASQTHVSTVVKGTFGYLDPEYYRRQI--LTEKSDVYSFGVVLLEVLCCRPIRMQSVPPEQAD---LIRWVKSNFNKRTVDQIIDSDLTADITSTSME : 773
AT5G39000 : IKTTNILLDENFVTKVSDFGLSRVGPTSASQTHVSTVVKGTFGYLDPEYYRRQV--LTEKSDVYSFGVVLLEVLCCRPIRMQSVPPEQAD---LIRWVKSNYRRGTVDQIIDSDLSADITSTSLE : 766
AT5G39020 : IKPQNILLDDNFCPKVADFGLAKLCEKRESILSLIDT-RGTIGYIAPEVVSRMYGGISHKSDVYSYGMLVLDMIGARNK--VETTTCNGSTAYFPDWIYKDLENGDQTWIIGDEINEE-DNKIVK : 740
AT5G39030 : IKPQNILLDGNLCPKVSDFGLAKLCEKRESVLSLMDT-RGTIGYIAPEVFSRMYGRVSHKSDVYSFGMLVIDMIGARSKEIVETVDSAASSTYFPDWIYKDLEDGEQTWIFGDEITKE-EKEIAK : 744
AT3G51550 : VKTTNILLDEKWVAKVSDFGLSKTGPTLD-HTHVSTVVKGSFGYLDPEYFRRQ--QLTEKSDVYSFGVVLFEALCARPALNPTLAKEQVS---LAEWAPYCYKKGMLDQIVDPYLKGKITPECFK : 780
AT3G04690 : VKTTNILVDENWVAKVSDFGLSKTGPNMN-GGHVTTVVKGSFGYLDPEYFRRQ--QLTEKSDVYSFGVVLFEILCARPALNPSLPKEQVS---LGDWAMNCKRKGNLEDIIDPNLKGKINAECLK : 760
AT5G28680 : VKTTNILLDENWVAKVSDFGLSKTGPNMN-GGHVTTVVKGSFGYLDPEYFRRQ--QLTEKSDVYSFGVVLFEVLCARPALNPSLSKEQVS---LGDWAMNCKRKGTLEDIIDPNLKGKINPECLK : 764
AT1G30570 : VKTTNILLDENFVAKMSDFGLSKAGPSMD-HTHVSTAVKGSFGYLDPEYFRRQ--QLTEKSDVYSFGVVLFEAVCARAVINPTLPKDQIN---LAEWALSWQKQRNLESIIDSNLRGNYSPESLE : 763
AT4G39110 : VKSTNILLDEALVAKVADFGLSKDVA-FG-QNHVSTAVKGSFGYLDPEYFRRQ--QLTDKSDVYSFGVVLLEALCARPAINPQLPREQVN---LAEWAMQWKRKGLLEKIIDPHLAGTINPESMK : 768
AT2G21480 : VKSTNILLDEALVAKVADFGLSKDVA-FG-QNHVSTAVKGSFGYLDPEYFRRQ--QLTDKSDVYSFGVVLLEALCARPAINPQLPREQVN---LAEWAMLWKQKGLLEKIIDPHLVGAVNPESMK : 767
AT5G61350 : VKTTNILLDENLVAKVSDFGLSKDAP-MD-EGHVSTAVKGSFGYLDPEYFRRQ--QLTDKSDVYSFGVVLFEVLCARPVINPQLPREQVN---LAEYAMNLHRKGMLEKIIDPKIVGTISKGSLR : 773
AT5G54380 : VKTTNILLDENLVAKVADFGLSKTGPSLD-QTHVSTAVKGSFGYLDPEYFRRQ--QLTEKSDVYSFGVVLMEVLCCRPALNPVLPREQVN---IAEWAMAWQKKGLLDQIMDSNLTGKVNPASLK : 753
AT5G59700 : VKSANILLDENLMAKVADFGLSKTGPEID-QTHVSTAVKGSFGYLDPEYFRRQ--QLTEKSDVYSFGVVMFEVLCARPVIDPTLTREMVN---LAEWAMKWQKKGQLEHIIDPSLRGKIRPDSLR : 725
AT3G46290 : VKSANILLDENFMAKVADFGLSKTGPELD-QTHVSTAVKGSFGYLDPEYFRRQ--QLTDKSDVYSFGVVLFEVLCARPVIDPTLPREMVN---LAEWAMKWQKKGQLDQIIDQSLRGNIRPDSLR : 728
AT2G39360 : VKSANILLDDNFMAKVADFGLSKTGPDLD-QTHVSTAVKGSFGYLDPEYLTRQ--QLTEKSDVYSFGVVMLEVVCGRPVIDPSLPREKVN---LIEWAMKLVKKGKLEDIIDPFLVGKVKLEEVK : 731
AT5G24010 : IKSTNILLDNNYVAKVADFGLSRSGPCID-ETHVSTGVKGSFGYLDPEYFRRQ--QLTDKSDVYSFGVVLFEVLCARPAVDPLLVREQVN---LAEWAIEWQRKGMLDQIVDPNIADEIKPCSLK : 732
AT2G23200 : VKSTNILLDEHNIAKVADFGLSKI-HNQD-ESNISINIKGTFGYLDPEYLQTH--KLTEKSDVYAFGVVLLEVLFARPAIDPYLPHEEVN---LSEWVMFCKSKGTIDEILDPSLIGQIETNSLK : 731

                   880         *       900         *       920         *       940         *       960         *       980         *
AT5G38990 : KFCEIAIRCVQDRGMERPPMNDVVWALEFALQLHETAKKKN----DNVESLDLMPSGEVGTTTDGEDDLFSRT----TGHVGKSTTTDDSVLVVGDERSGSSWGVFSEINEPKAR : 880
AT5G39000 : KFCEIAVRCVQDRGMERPPMNDVVWALEFALQLHETAKKKN----DNVESLDLMPSGEVGTTTDGEDDLFSRT----TGHVGKSTTTDDSVLVVGDERSGSSWGVFSEINEPKAR : 873
AT5G39020 : KMILVSLWCIRPCPSDRPPMNKVV-------------EMIE----GSLDALELPP----------------KP----SRHISTELVLESSSLSDGQEAEKQT-----QTLDSTII : 813
AT5G39030 : KMIVVGLWCIQPCPSDRPSMNRVV-------------EMME----GSLDALEIPP----------------KP----SMHISTEVITESSSLSDGGED----------------V : 806
AT3G51550 : KFAETAMKCVLDQGIERPSMGDVLWNLEFALQLQESAEENGKGVCGDMDMDEIKYDDGNCKGKNDKSSDVYEGNVTDSRSSGIDMSIGGRSLASEDSDGLTPSAVFSQIMNPKGR : 895
AT3G04690 : KFADTAEKCLNDSGLERPTMGDVLWNLEFALQLQETADGT--------------------RHRTPNNGGSSEDLGRGGMAVNV---AGRDDV-SD-LSSEDNTEIFSQIVNPKGR : 850
AT5G28680 : KFADTAEKCLSDSGLDRPTMGDVLWNLEFALQLQETADGS--------------------RHRTPSNGGGSVDLGGGGGGVTVNISAGESDL-GDDLSSEENSGIFSQIVNPKGR : 858
AT1G30570 : KYGEIAEKCLADEGKNRPMMGEVLWSLEYVLQIHEAWLRKQNGE-NSFSSSQAV----------------EEAPESFTLPACSN---QDSSETEQSQTGSAL---------HNSA : 849
AT4G39110 : KFAEAAEKCLEDYGVDRPTMGDVLWNLEYALQLQEAFTQGKAEE-TENAKPDVVTPGSVPVSDPSPITPSVTTNEAATVPVPAKVE-ENSGTAVDEHSGTAM---FTQFANLNGR : 878
AT2G21480 : KFAEAAEKCLADYGVDRPTMGDVLWNLEYALQLQEAFSQGKAEA-EE-----VETPKPVAVPAAAPTSPAATTAAASERPVS-QTE-EKDDSTVDQHSGTTM---FTQFASLNGR : 871
AT5G61350 : KFVEAAEKCLAEYGVDRPGMGDVLWNLEYALQLQEASAQVDLSE-DKTTM----------------------------------------NIEMDLIPGEEM-----QSPSHSIP : 842
AT5G54380 : KFGETAEKCLAEYGVDRPSMGDVLWNLEYALQLEETSS--------ALMEPDDNSTNHIPGIPMAPMEPFDNS-----MSIIDRGGVNSGTGTDDDAEDATTSAVFSQLVHPRGR : 855
AT5G59700 : KFGETGEKCLADYGVDRPSMGDVLWNLEYALQLQEAVV--------D-GDPED-STNMIGELPL-RFNDYNHGDTSVNFSVAKEGRFDEEESSVDDSSGVSMSKVFSQLIKSEGR : 829
AT3G46290 : KFAETGEKCLADYGVDRPSMGDVLWNLEYALQLQEAVI--------D-GEPEDNSTNMIGELPP-QINNFSQGDTSVNVP-GTAGRF--EESSIDDLSGVSMSKVFSQLVKSEGR : 830
AT2G39360 : KYCEVTEKCLSQNGIERPAMGDLLWNLEFMLQVQ----------------AKDEKAAMVDDKPEASV-------------VGSTMQFSVN--GVGDIAGVSMSKVFAQMVREETR : 815
AT5G24010 : KFAETAEKCCADYGVDRPTIGDVLWNLEHVLQLQES-GPLNIPE-EDYGDVTDPRTARQGLSNGSNIER----------------DYGDGTSGI-----ISSTQVFSQLMTNAGR : 824
AT2G23200 : KFMEIAEKCLKEYGDERPSMRDVIWDLEYVLQLQMMTNRREAHE------EDSTAINSGGSLVAPRL------MVSDSFSTNSIFQNGDESKNRFGFTDSSETRVFSQLKISDAR : 834
```

homology of *At5g39024* to *MDS3* and *MDS4*

(c) Phylogenetic analysis using the full length CrRLK1L proteins

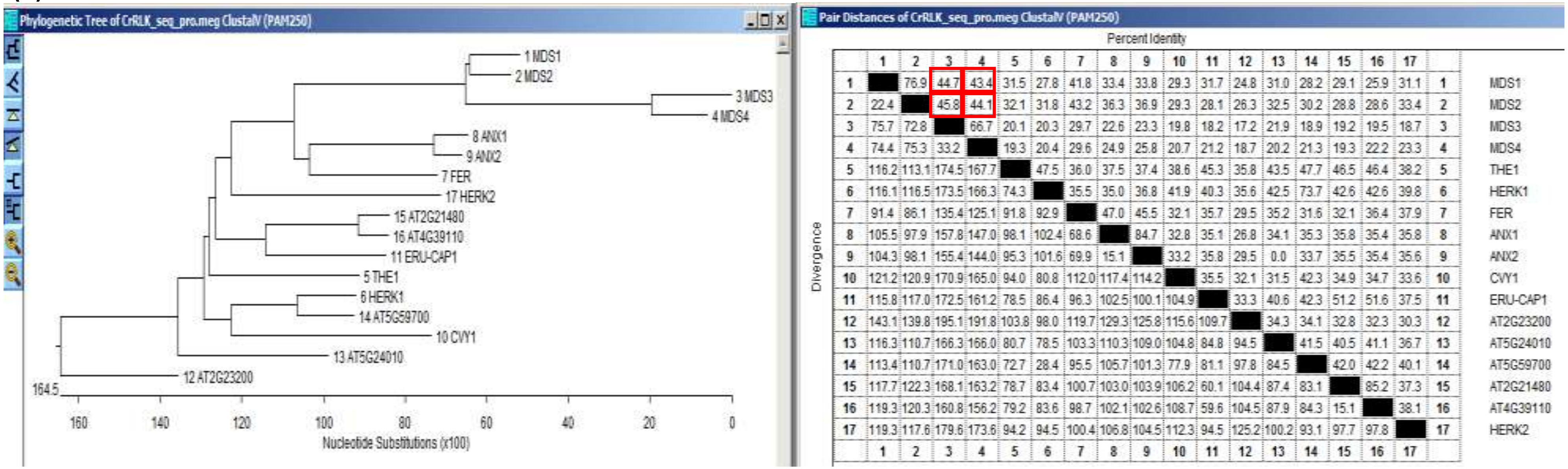

(d) Phylogenetic analysis using the extracellular domain of CrRLK1L proteins

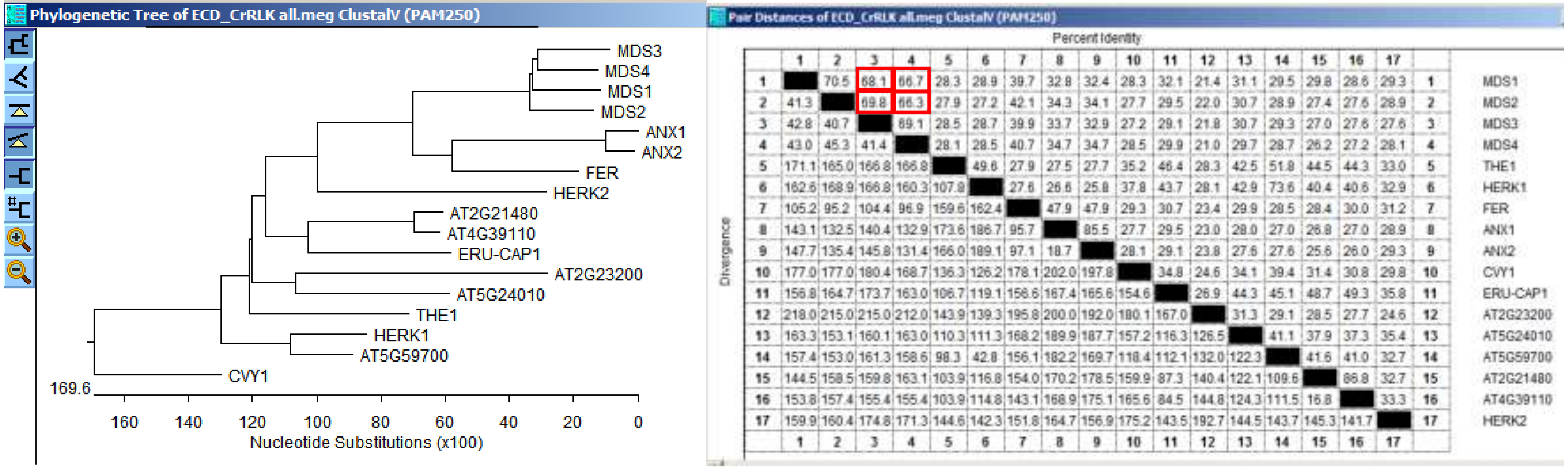

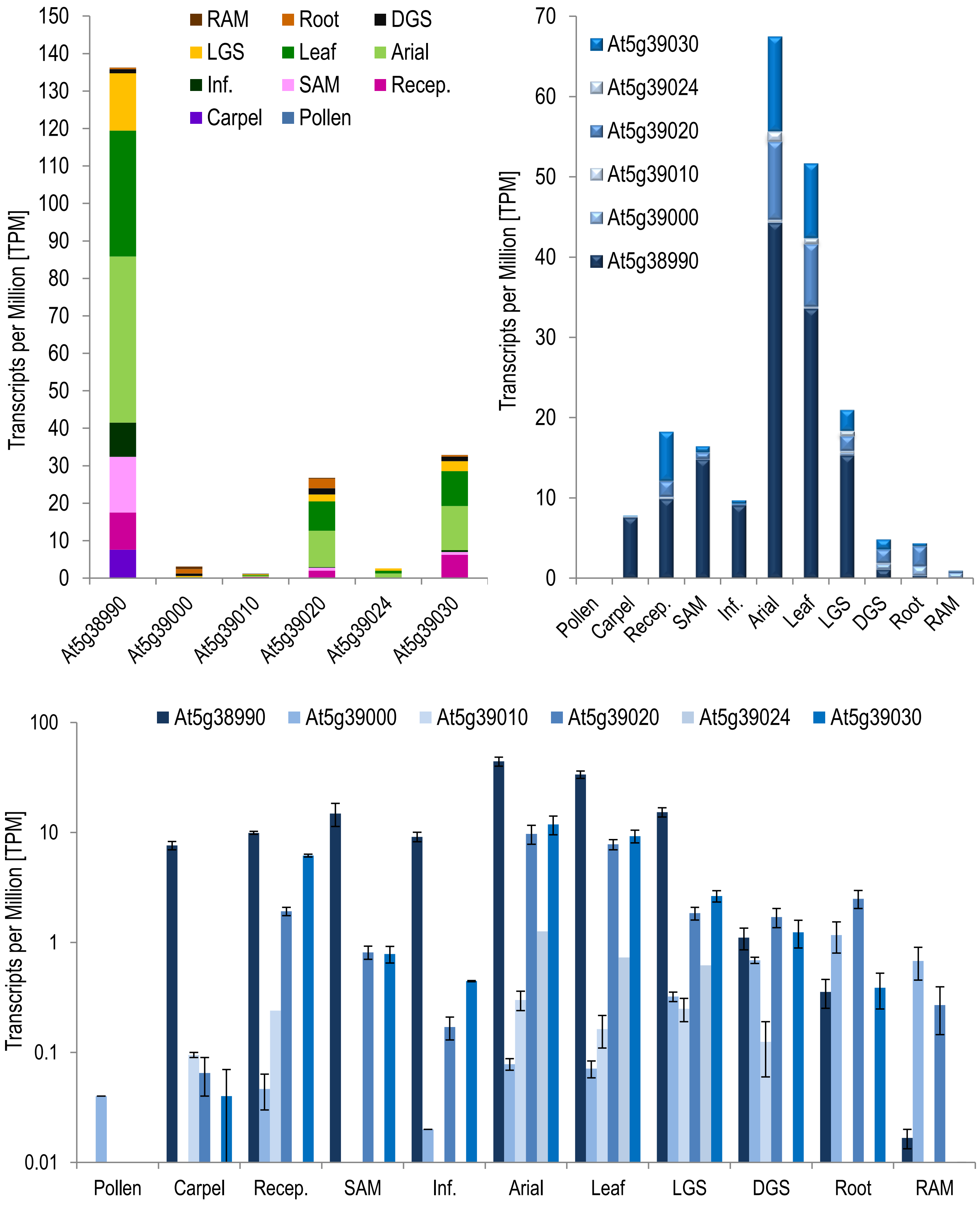

Figure S2. RNA-Seq data of 113 anatomical parts and growth condition of Arabidopsis Col-0 from the Araport database via the ThaleMine data warehouse. Pollen, Mature pollen; Carpel from stage 8-13 flowers; Recept., Receptacle from base of stage 15 flower of long-day-grown plants; SAM, Shoot apical meristem/young leaf from 14 days old plant grown in simulated sun condition; Infl., stage-12 floral buds from long-day grown plant; Aerial part of long-day-grown 4-leaf-stage seedlings; Leaf of 3-week-old long-day-grown plants; LGS, 7 days old plate-grown long-day seedlings; DGS, 7 days old dark-grown seedlings; Root from 4-leaf-stage seedlings; RAM, Root tip from 3 days old seedlings.

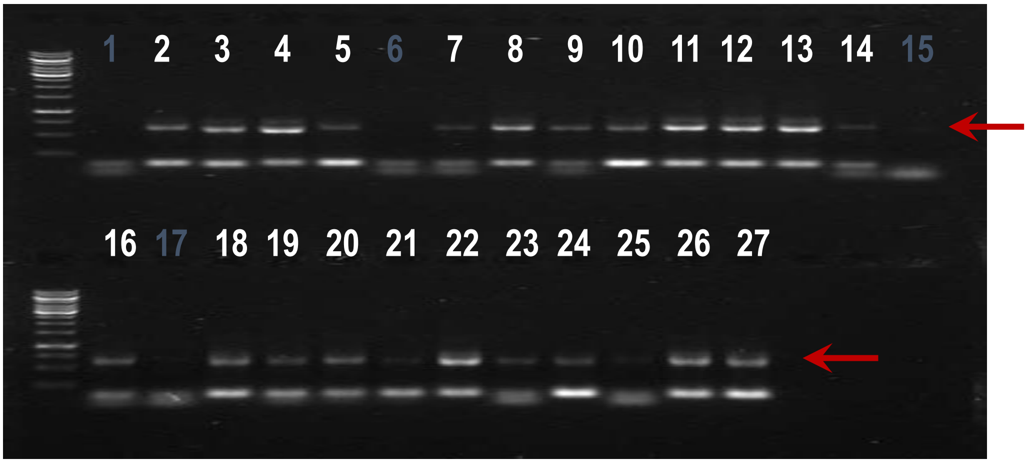

Figure S3. Test PCR of 27 transgenic plants of the T1 generation for a deletion between *MDS1* and *MDS4* with primers At5g38990 F-VS and At5g39030 R-VS. The gel shows that, in 23 out of the 27 plants a 300 bp to 500 bp fragment was amplified which is indicative of the roughly 11 kb deletion from *MDS1* to *MDS4*.

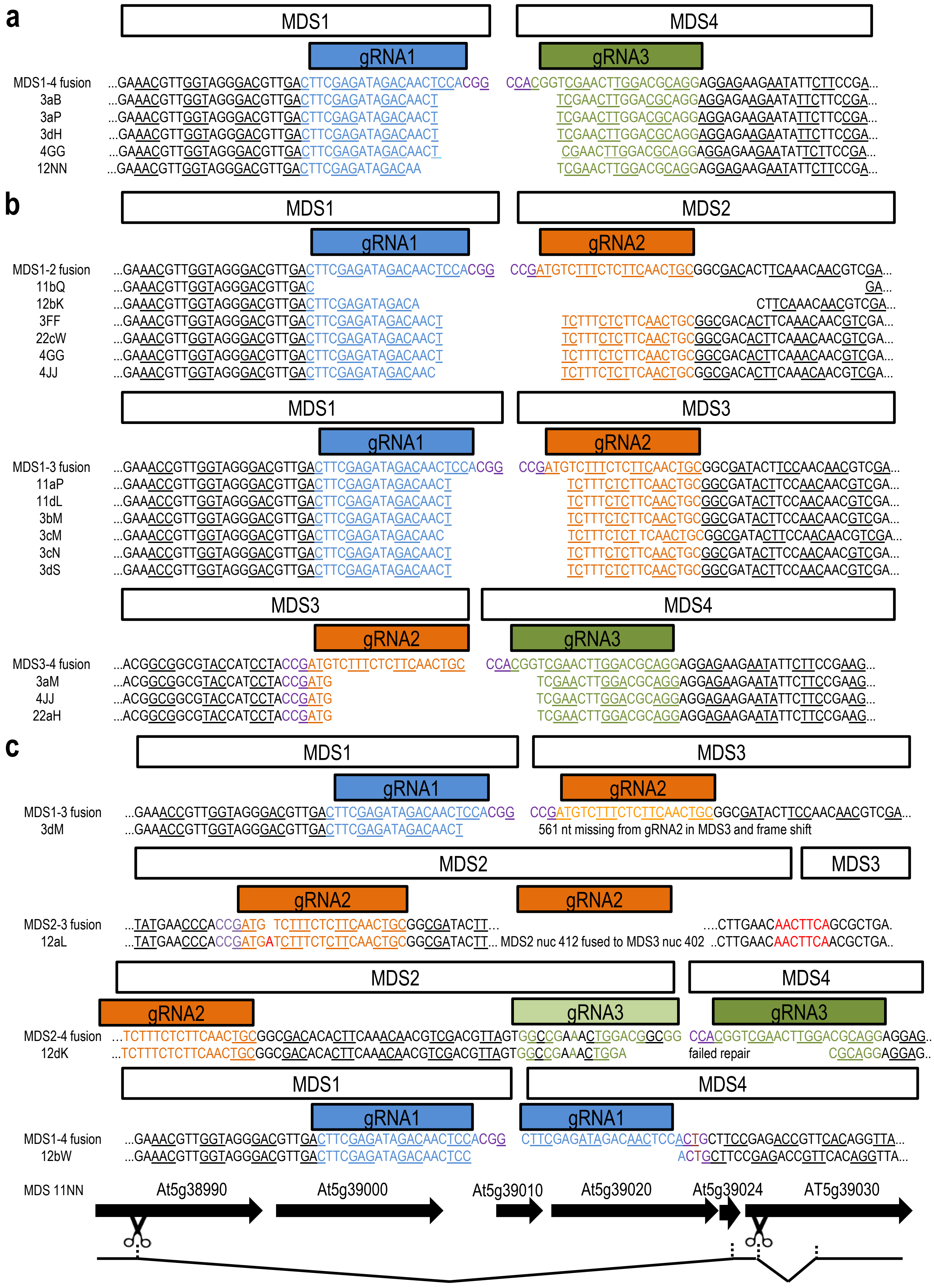

a
MDS1
MDS4
gRNA1
gRNA3
MDS1-4 fusion
3aB
3aP
3dH
4GG
12NN
b
MDS1
MDS2
gRNA1
gRNA2
MDS1-2 fusion
11bQ
12bK
3FF
22cW
4GG
4JJ
MDS1
MDS3
MDS1-3 fusion
11aP
11dL
3bM
3cM
3cN
3dS
MDS3
MDS4
MDS3-4 fusion
3aM
4JJ
22aH
c
MDS1
MDS3
MDS1-3 fusion
3dM
561 nt missing from gRNA2 in MDS3 and frame shift
MDS2
MDS3
MDS2-3 fusion
12aL
MDS2 nuc 412 fused to MDS3 nuc 402
MDS2
MDS4
MDS2-4 fusion
12dK
failed repair
MDS1
MDS4
MDS1-4 fusion
12bW
MDS 11NN
At5g38990
At5g39000
At5g39010
At5g39020
At5g39024
AT5g39030

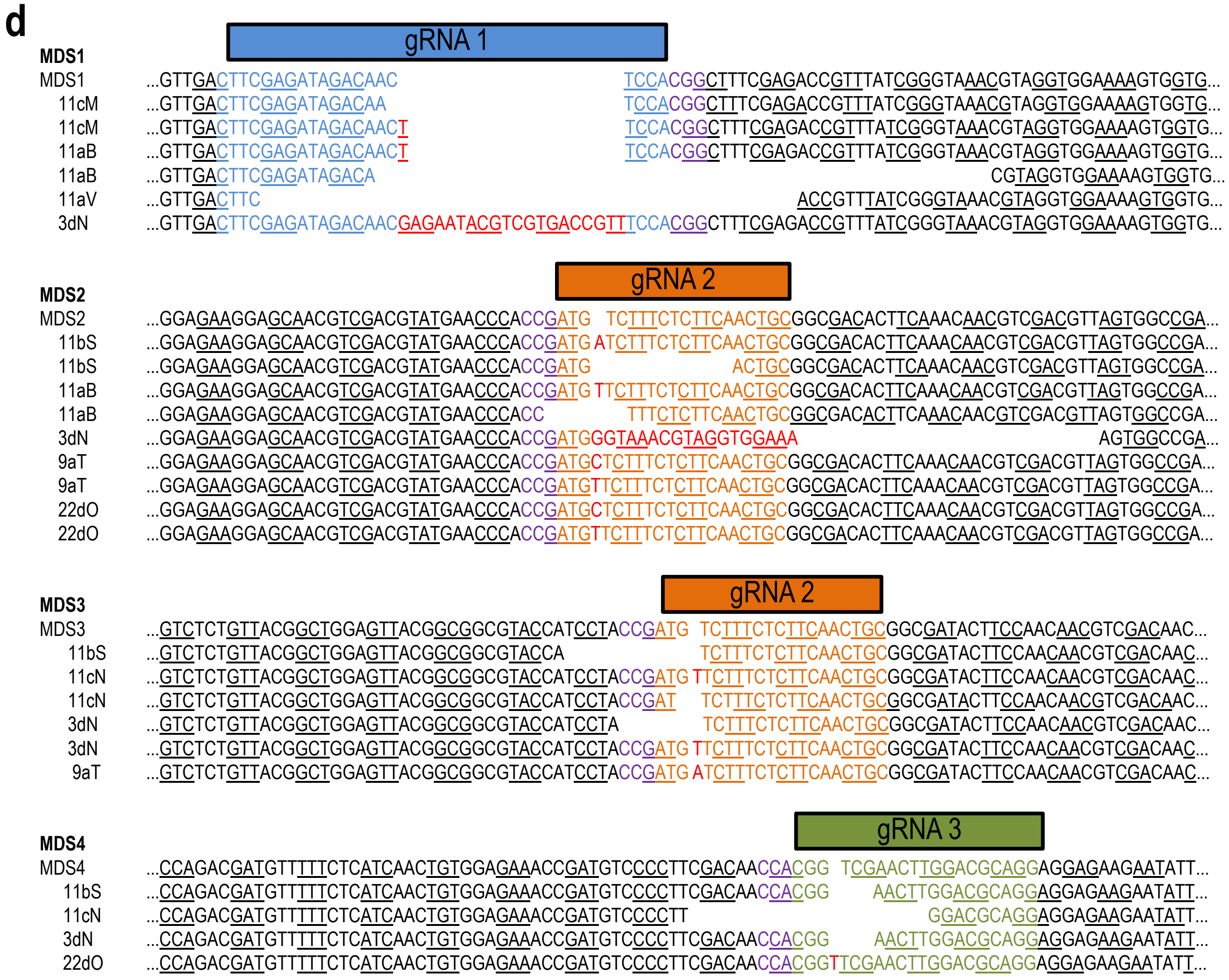

Figure S4. Sequences of the CRISPR/Cas9 edited *MDS* cluster. **(a)** Deletions between *MDS1* and *4*, **(b)** smaller gene deletions, **(c)** unexpected sites of deletions and **(d)** mutations within *MDS* genes. gRNAs are marked by colored boxes and sequences, PAM motifs are indicated in purple. Underlined letters indicate the reading frames.

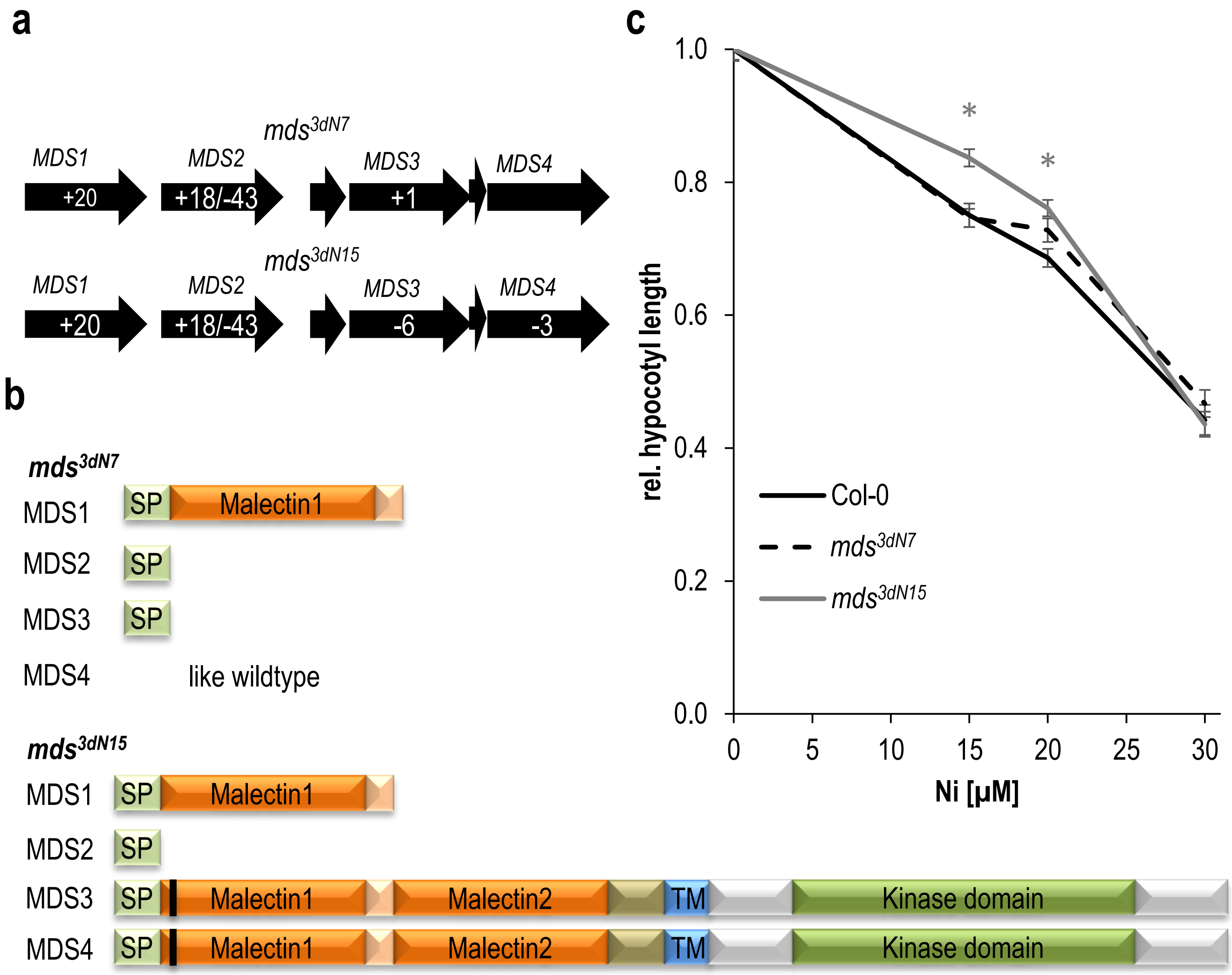

Figure S5. Characterization of the CRISPR/Cas9 edited mutants *mds*^{3dN7} and *mds*^{3dN15}. **(a)** Schematic representation of the mutations. Numbers indicate deleted or inserted bases.**(b)** Domain structure of the putative truncated proteins. Black bars represent deletions. **(c)** Etiolated hypocotyl length on media supplemented with different concentrations of Ni^{2+}. Represented are means +/- SEM of up to 60 seedlings from three independent experiments. Stars indicate significant difference according to Student's t-test with (*) $p < 0.05$ to wildtype.

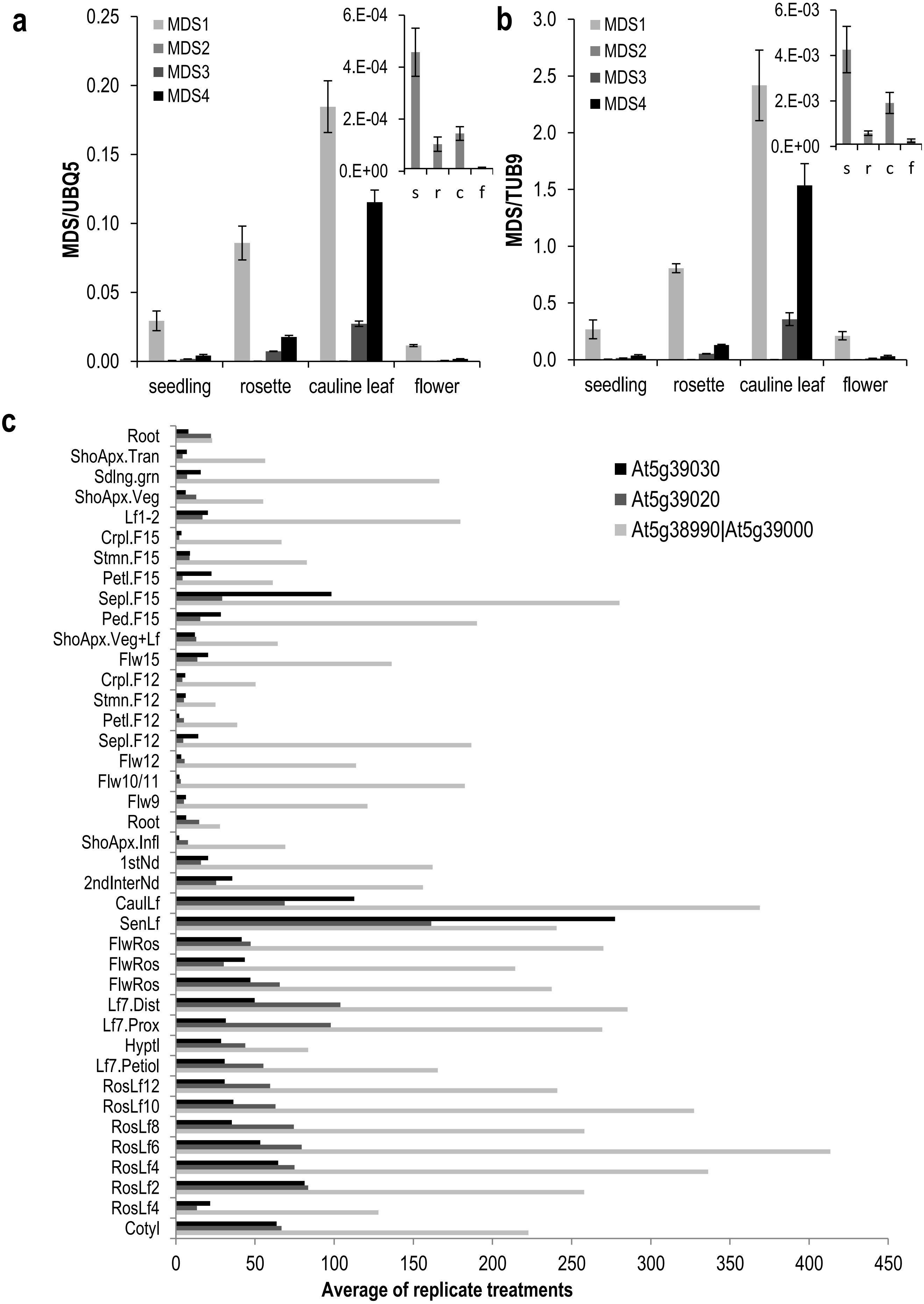

Figure S6. Expression of the four *MDS* genes during development. Shown are the means and standard errors (SE) of RT-qPCR data of at least two biological replicas and three technical repeats normalized to the reference gene *UBQ5* **(a)** and *TUB9* **(b)**. **(c)** Microarray data of the development series deposited in the BAR database..

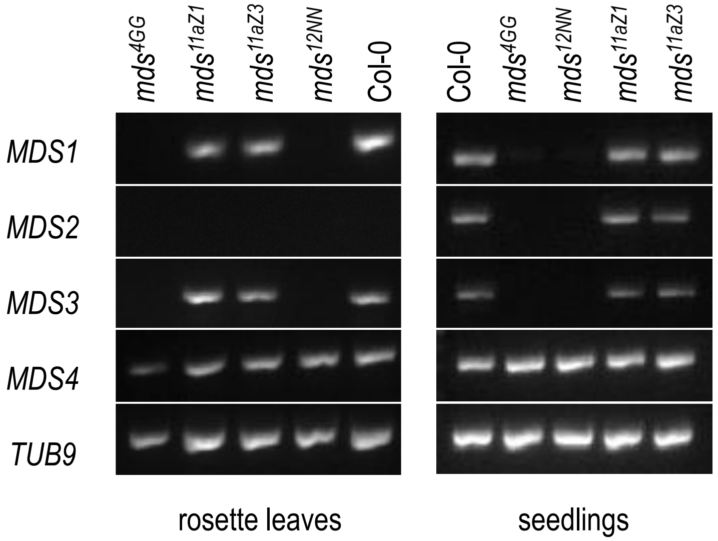

Figure S7. Expression analysis of the putatively truncated or chimeric proteins. RT-PCR results of cDNA from rosette leaves and whole seedlings grown for eight days on MS2.5. cDNA was reverse transcribed from 2 to 3 µg of RNA and diluted 1:10 and 1:5 prior to RT-PCR, respectively. PCR was performed using Phusion Taq (Thermo Fisher Scientific) for 40 cycles

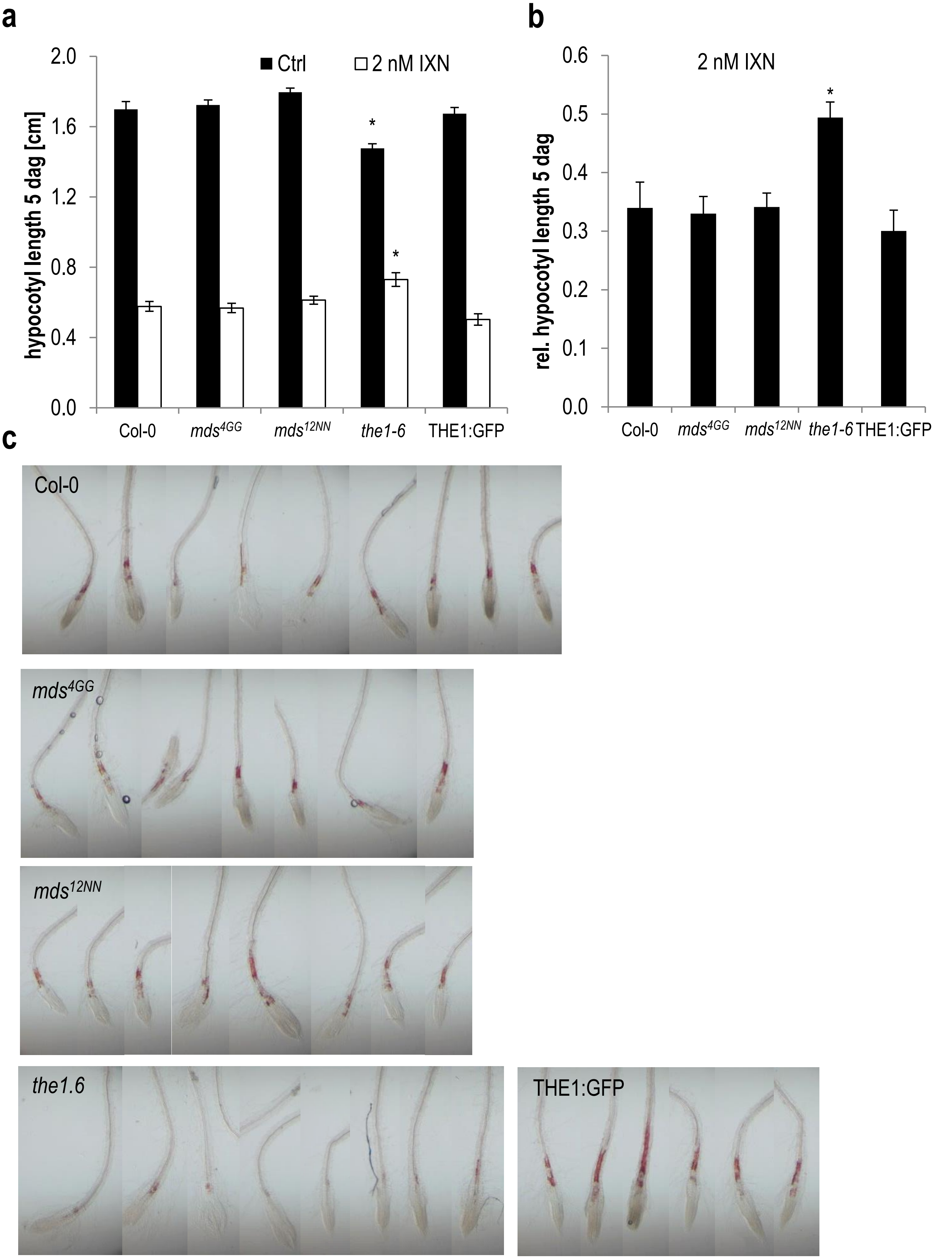

Figure S8. Phenotypes upon isoxaben treatment**. (a)** Etiolated hypocotyl length, mean of 18-20 seedlings +/- SEM. Stars indicate significant difference according to Student's t-test with (*) $p < 0.05$ to wildtype. **(b)** Hypocotyl length in relation to control medium. **(d)** Lignin staining of seedling roots exposed overnight to 600 nM isoxaben.

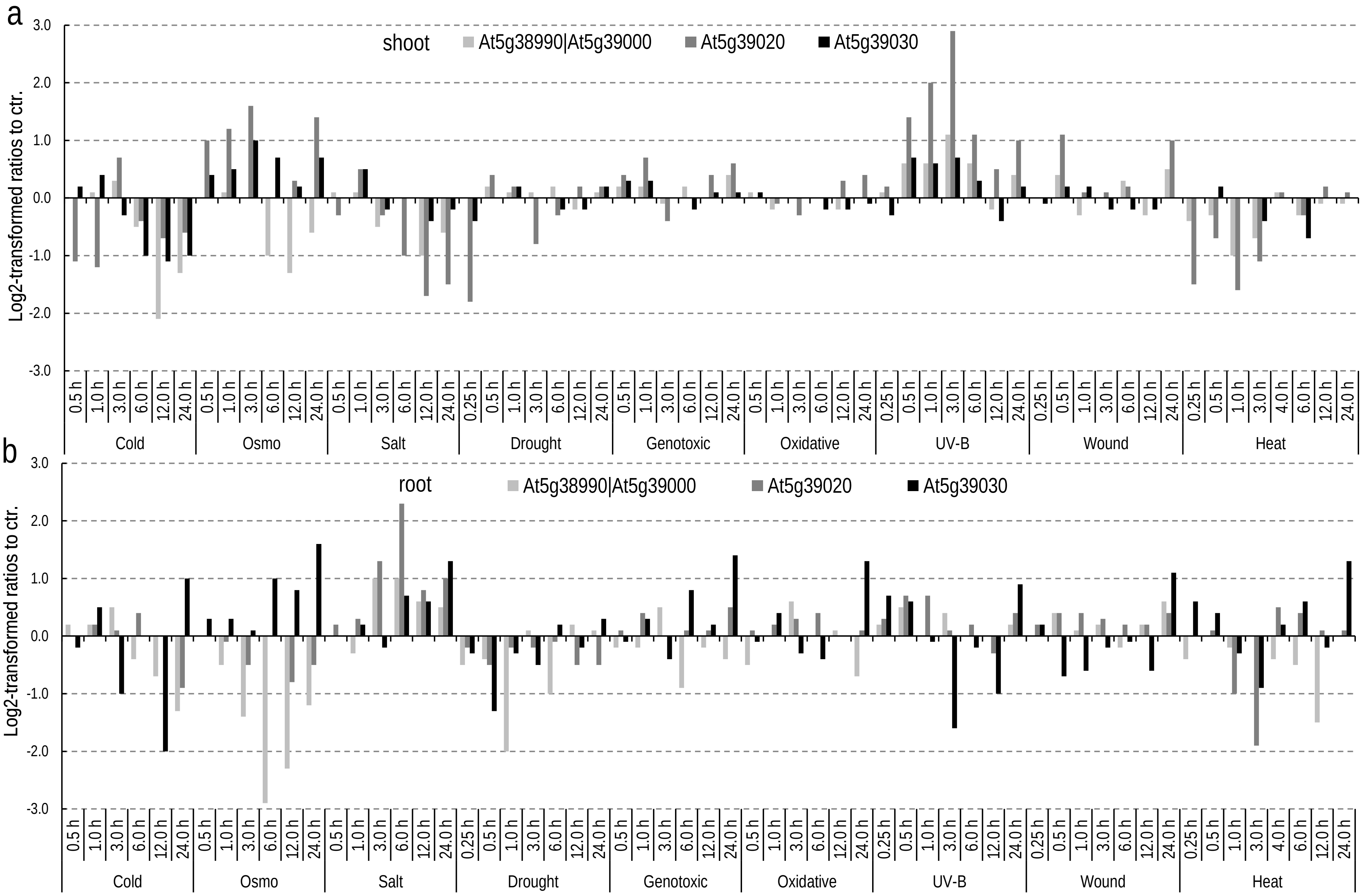

Figure S9. Expression of the *MDS* genes upon abiotic stressors. Shown are the microarray data of the abiotic stress series deposited in the BAR database for (a) shoot and (b) root tissues.

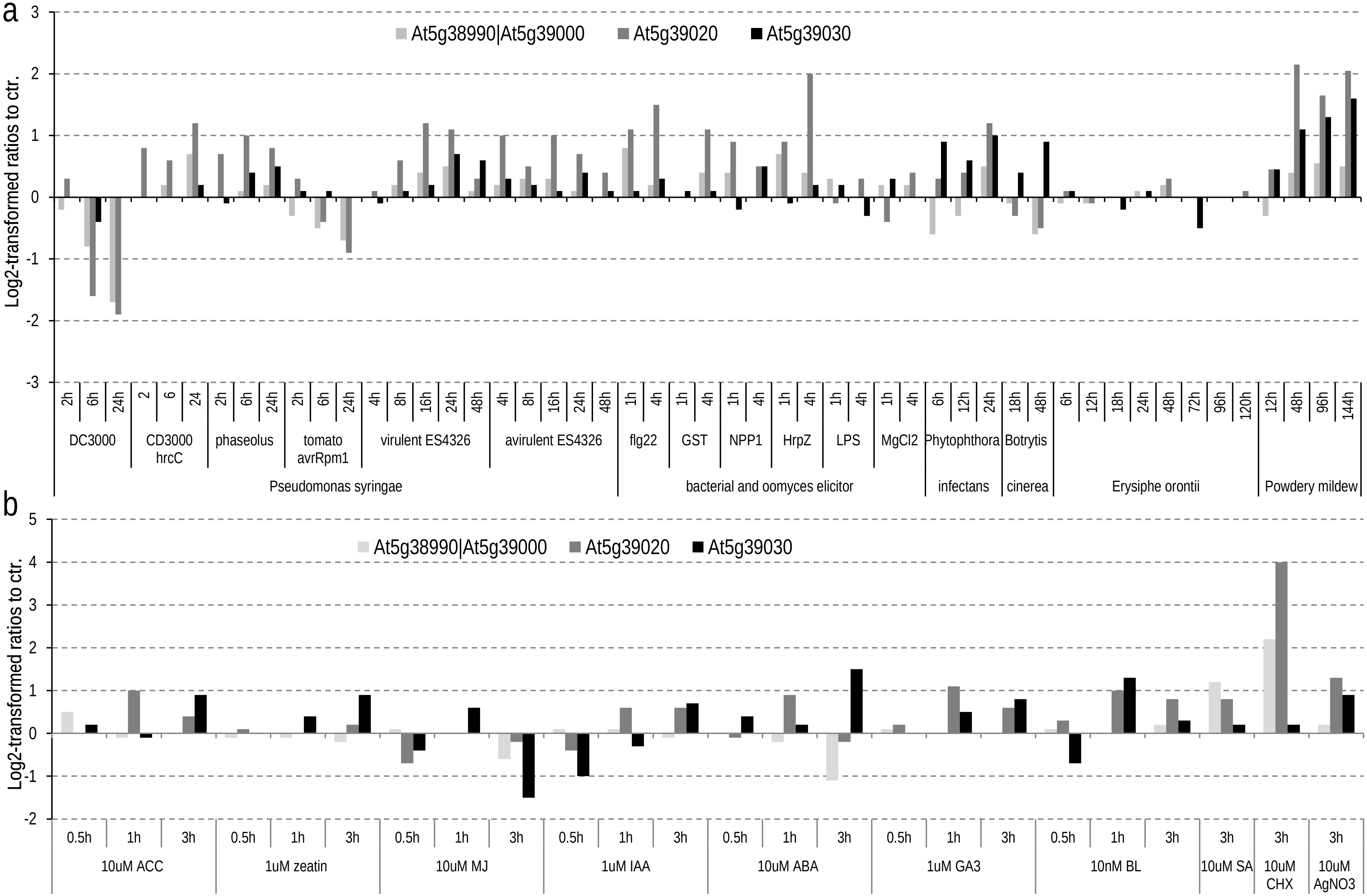

Figure S10. Expression of the *MDS* genes upon biotic stressors and hormone treatments. Shown are the microarray data of the (a) biotic stress series and the (b) hormone experiments deposited in the BAR database.
